# Design, Synthesis, and Anti-Bacterial Evaluation of Triazolyl-Pterostilbene Derivatives

**DOI:** 10.3390/ijms20184564

**Published:** 2019-09-14

**Authors:** Kai-Wei Tang, Shih-Chun Yang, Chih-Hua Tseng

**Affiliations:** 1 School of Pharmacy, College of Pharmacy, Kaohsiung Medical University, Kaohsiung 807, Taiwan; dadaking1107@gmail.com; 2 Department of Cosmetic Science, Providence University, Taichung 433, Taiwan; 3 Drug Development and Value Creation Research Center, Kaohsiung Medical University, Kaohsiung 807, Taiwan; 4 Department of Fragrance and Cosmetic Science, College of Pharmacy, Kaohsiung Medical University, Kaohsiung 807, Taiwan; 5 Department of Medical Research, Kaohsiung Medical University Hospital, Kaohsiung 807, Taiwan; 6 Department of Pharmacy, Kaohsiung Municipal Ta-Tung Hospital, Kaohsiung 801, Taiwan

**Keywords:** pterostilbene, triazole, MRSA, antibacterial

## Abstract

*Staphylococcus aureus* resistance to current antibiotics has become the greatest global challenge facing public health. The development of new antimicrobial agents is urgent and important and is needed to provide additional therapeutic options. In our previous study, we found out that pterostilbene exhibited potent antibacterial activity, especially against methicillin-resistant *Staphylococcus aureus* (MRSA). According to previous studies, 1,2,3-triazole, with the characteristic of increasing the interaction with the target readily and enhancing water solubility, were widely used in the approved anti-bacterial drugs. Therefore, these results attract our interest to use the structure of pterostilbene as a scaffold for the hybrid 1,2,3-triazole moiety to develop a novel anti-MRSA infection agent. In this study, we demonstrated the design and synthesis of a series of triazolylpterostilbene derivatives. Among these compounds, compound **4d** exhibited the most potent anti-MRSA activity with a minimum inhibitory concentration (MIC) value of 1.2–2.4 μg/mL and a minimum bactericidal concentration (MBC) value of 19.5–39 μg/mL. The structure–activity relationship and antibacterial mechanism were investigated in this study. Molecular docking studies were carried out to verify and rationalize the biological results. In this study, the results confirmed that our design could successfully increase the inhibitory activity and specificity against MRSA. Compound **4d** could be used as a candidate for anti-bacterial agents and in depth vivo studies should be further investigated.

## 1. Introduction

The global dissemination and rising populations of antimicrobial resistance (AMR) bacteria are the greatest global challenges facing public health. More than 2 million people are infected by drug resistance pathogens in the world and cause over 700,000 deaths each year [1,2]. In Europe, it costs about 1.5 billion euros per year to solve the infection problem of drug resistant bacteria in which methicillin-resistant *Staphylococcus aureus* (MRSA) is the most common multi-drug resistant strain [3]. In the United States, approximately 23,000 people die from drug-resistant infection each year [4,5].

*Staphylococcus aureus* (*S. aureus*) is an opportunistic pathogen that is found in the human skin and mucosa. Once the immunity of a patient, who needs a catheter or ventilator or must undergo surgery, is reduced, *S. aureus* can enter the body and cause infection. Currently, the main treatment for *S. aureus* infection is antibiotic treatment [6,7]. However, when *S. aureus* becomes resistant to methicillin, vancomycin, or other antibiotics, there are few therapeutic options available. Therefore, the development of new antimicrobial agents is very urgent and important. Modern antibacterial drug discovery is associated with a variety of targets such as DNA polymerases and topoisomerases, or those that affect cell-wall synthesis [8,9].

Pterostilbene, found primarily in blueberries and *Pterocarpus marsupium* heartwood, may have numerous preventive properties and therapeutic targets, including cardiovascular, neurological, metabolic, carcinogenic, and bacterial disorders [10]. Pterostilbene, with high similarity to the structure of resveratrol, a compound harvested in red wine, possesses comparable anti-inflammatory, anti-oxidant, and anti-carcinogenic properties, and exhibits increased bioavailability compared to resveratrol. Research showed that pterostilbene has 80% bioavailability compared to 20% for resveratrol in animal models due to the presence of two methoxy groups causing an increase of lipophilic and oral absorption [11]. Our previous study also showed that pterostilbene exhibited more efficient bacterial eradication than resveratrol and also showed a six-fold higher cutaneous absorption compared to resveratrol [12]. Therefore, these results attract our interest to use the structure of pterostilbene as a scaffold to develop the anti-MRSA infection agent.

**Figure 1:**
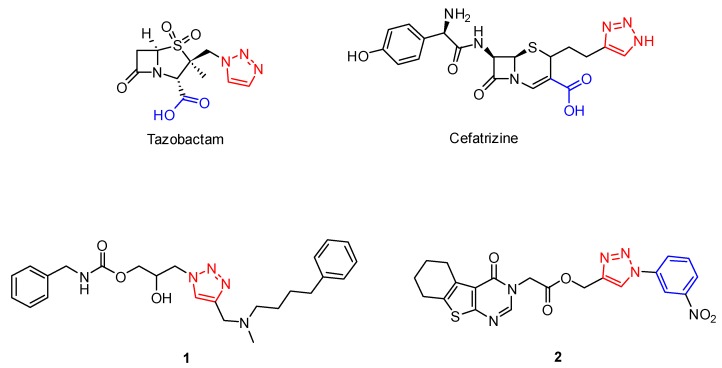
The structure of anti-bacterial agents containing the 1,2,3-triazole moiety based on FDA approved drugs and previous studies.

The compound 1,2,3-triazole, an important pharmacophore in modern medicinal chemistry research, with the characterization of structural stability and metabolic resistance, is widely used in drug discovery. Many therapeutic drugs approved by the United States Food and Drug Administration already use the 1,2,3-triazole moiety as an important pharmacophore, such as the anti-epileptic agent rufinamide, and anti-bacterial agents tazobactam and cefatrizine [13,14]. Moreover, 1,2,3-triazole possess several advantages. For example, it increases the interaction with targets by forming hydrogen bonds readily and enhances the water solubility by raising the polarity of the compound [15,16,17]. It also exhibits a wide range of pharmacological properties including anti-cancer, anti-viral, anti-tubercular, and anti-bacterial activities [18]. Previous research in which compounds **1** and **2** contained the 1,2,3-triazole moiety showed comparable anti-bacterial activity (Figure 1) [19,20]. Hence, we believe the insertion of 1,2,3-triazole in the scaffold may lead to the potential active compounds.

**Figure 2:**
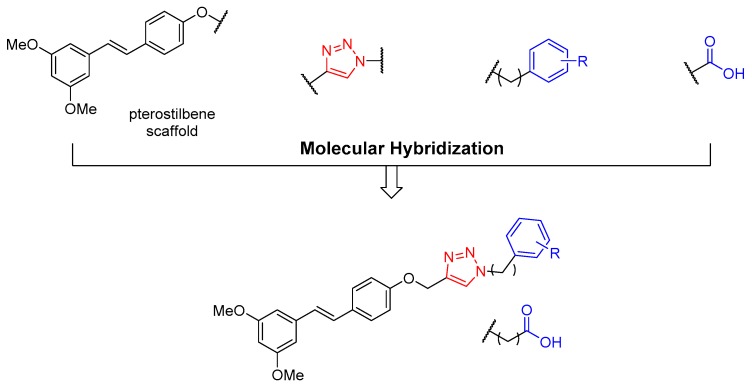
Design strategy of novel hybrid pterostilbene derivatives.

With the promising anti-bacterial properties of pterostilbene and 1,2,3-triazole, in this study we sought to develop potent anti-MRSA agents by using pterostilbene as a scaffold. Thus, 1,2,3-triazole was used as a bridge to connect the substituted aryl group or carboxylic group (Figure 2). A novel hybrid series of derivatives, compounds **4**–**7**, were carried out for antibacterial biological evaluation to identify the most potent compound. Molecular docking studies were performed to validate the biocidal action between the crystallographic structure of the target proteins and the lead compounds to elucidate the active binding sites on enzymes.

## 2. Results and Discussion

### 2.1. Chemistry

**Scheme 1:**
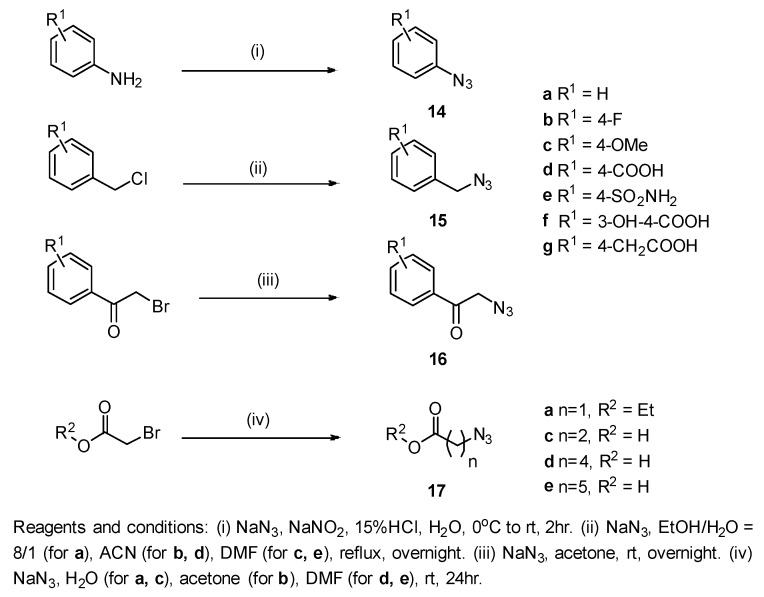
Synthesis of azide derivatives **14**–**17** series.

**Scheme 2:**
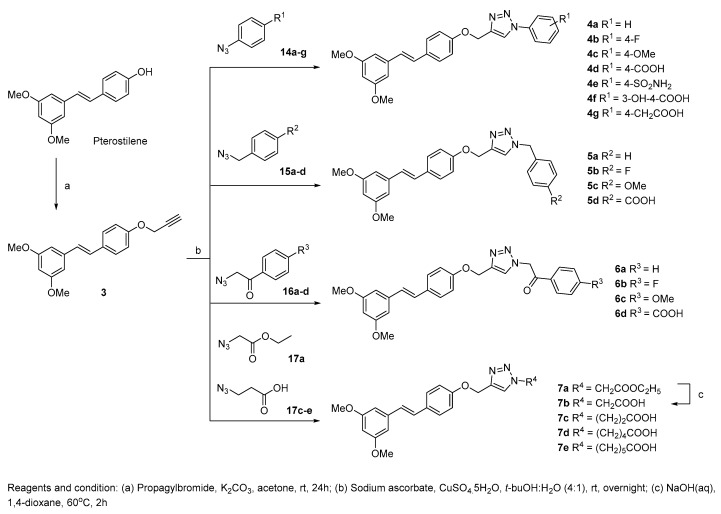
Synthesis of triazolyl pterostilbene derivatives **4**–**7** series via Click reaction.

The preparation of selected azide is shown in Scheme 1, and the synthesized approach was carried out according to previous studies [21,22,23,24,25]. Pterostilbene was used as starting material to react with propargyl bromide in the condition of potassium carbonate and acetone to yield compound **3**. After the selected azide was obtained, the click reaction was carried out with compound **3** in the presence of sodium ascorbate, copper sulfate pentahydrate, and *tert*-butanol at room temperature for 8 h. The synthetic routes for compounds **4**–**7** series are depicted in Scheme 2. The structure of the tested compounds was examined by NMR spectrometer (spectra data can be found in Supplementary Materials) and mass spectrometry. The purity of the tested compounds was determined by HPLC analysis. All of the detailed information is provided in Section 4.

### 2.2. Biological Evaluation

#### 2.2.1. Anti-MRSA and VISA Activity of **4d**, **5d**, **7c**, **7d**, and **7e**

Agar diffusion assay was first used to screen the anti-MRSA and anti- vancomycin-intermediate *Staphylococcus aureus* (VISA) activity of all 20 synthesized compounds. Among them, five of the compounds, **4d**, **5d**, **7c**, **7d**, and **7e**, exhibited the antimicrobial activity against MRSA and VISA. At 5000 μg/mL, compounds **7d** and **7e** provided the largest inhibition zone to inhibit MRSA growth with diameters of 21.43 and 20.88 mm, respectively. In similar results against VISA, compounds **7d** and **7e** had the largest inhibition zones with diameters of 16.94 and 17.19 mm, respectively (Table 1). Compounds **4d**, **5d**, and **7c** only had mild inhibition zones in the anti-MRSA and anti-VISA agar diffusion tests. The results could infer poor water solubility in the surface of agar, which may have limited the inhibitory activity.

Among the results of preliminary antimicrobial evaluation by agar diffusion assay, compounds **4d**, **5d**, **7c**, **7d**, and **7e** exhibited inhibition zones that were chosen to test minimum inhibitory concentration values and minimum bactericidal concentration values against MRSA and VISA. Pterostilbene was used as a positive control. Minimum inhibitory concentration (MIC) values and minimum bactericidal concentration (MBC) values are given in Table 2. Compound **4d** exhibited strong anti-MRSA activity and showed lower values of MIC (1.2–2.4 μg/mL) than **7d** (78.1 μg/mL), **7e** (78.1 μg/mL), **5d** (312.5–625 μg/mL), and **7c** (625 μg/mL). Compound **4d** also had the lowest value of MBC (19.5–39 μg/mL) against MRSA than pterostilbene and other four compounds. Both compounds **7d** and **7e** showed comparable MIC values (78.1 μg/mL) against MRSA. However, the MBC value of these two compounds was over 1250 μg/mL. Therefore, it was speculated that the antibacterial activity of compounds **7d** and **7e** was to inhibit the growth of MRSA rather than bactericidal action. The other two compounds, **5d** and **7c**, had poor anti-MRSA activity with MBC values exceeding 625 μg/mL. On the other hand, in the anti-VISA activity test, all of the compounds had high values of MIC (≥156 μg/mL) and MBC (≥1250 μg/mL) (Table 2), indicating that our synthesized compounds would specifically act on MRSA.

**Figure 3:**
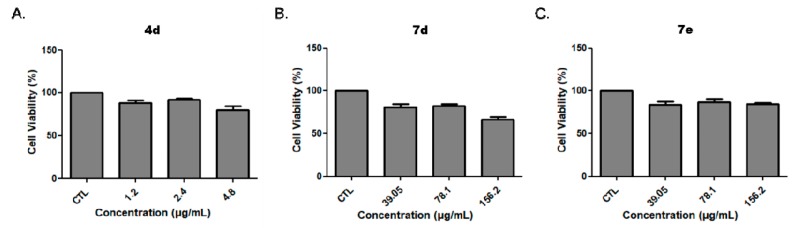
Viability assay of HaCaT cells with different doses of compounds. (**A**) Compound **4d**; (**B**) compound **7d**; (**C**) compound **7e**. Cell viability is measured by CCK-8 assay compared to the control (CTL) group. Results are expressed as the mean ± SEM (*n* = 3).

The effects of compounds **4d**, **7d**, and **7e** on the cytotoxicity of human HaCaT keratinocyte cells were determined by a CCK8 assay (Figure 3). Different concentration of compounds **4d**, **7d**, and **7e** (0.5×, 1×, and 2× MIC) were used to challenge HaCaT keratinocyte cells. Compound **4d** exhibited mild cytotoxicity at concentrations of 1.2 and 2.4 μg/mL with 88.2% and 91.9% cell viability, respectively. HaCaT cell viability was reduced to 80.1% when the compound **4d** concentration was increased to 4.8 μg/mL (Figure 3A). Similar results are shown in Figure 3B, indicating that compound **7d** exhibited mild cytotoxicity at concentrations of 39.05 and 78.1 μg/mL with >80.4% cell viability. However, the cell viability was reduced to 66.7% with a 156.2 μg/mL concentration of compound **7d**. Compound **7e** also showed mild cytotoxicity at concentration 39.05–156.2 μg/mL with >83.4% cell viability (Figure 3C). This indicates a mild toxicity of compounds **4d**, **7d**, and **7e** to HaCaT cells at 2X MIC concentrations.

#### 2.2.2. Structure-Activity Relationship

From the results of MIC values, we determined the structure-activity relationship. Five of the tested compounds, **4d**, **5d**, **7c**, **7d**, and **7e**, with anti-MRSA activity had some structural features in common: all of these compounds contained a carboxylic acid moiety at the terminal site. The other tested compounds with halogen or electron-donating substituent showed no activity against MRSA, indicating the carboxylic acid must be the essential moiety in the antibacterial activity. The space between triazole and the carboxylic acid group was also an important point. Compounds **7d** and **7e** with a spacer of four to five carbons exhibited comparable anti-MRSA activity. The anti-MRSA activity decreased in the order of **7d** = **7e** (78.1 μg/mL) > **7c** (625 μg/mL) > **7b**, indicating longer a spacer with four to five carbons was more active than the two carbons. Compound **7b**, with only one carbon as a spacer, lost the inhibitory activity dramatically. On the other hand, compound **4d**, with a phenyl group as the spacer, had the most potent anti-MRSA activity. The anti-MRSA activity decreased in the order of **4d** (1.2–2.4 μg/mL) > **5d** (312.5–625 μg/mL) > **6d**, indicating the derivatives with the phenyl group as the spacer between triazole and carboxylic acid was more active than the benzyl group, which in turn was more active than the phenacyl group with no anti-bacterial activity. Therefore, from the results among these derivatives, we could speculate that the spacer between triazole and carboxylic acid plays an important role in the inhibition of MRSA. Phenyl groups with the characteristics of planarity and resonance would be the most favorable spacer.

#### 2.2.3. Compounds **4d**, **7d**, and **7e** Treatment did not Affect Bacterial Cell Wall and Cell Membrane

**Figure 4:**
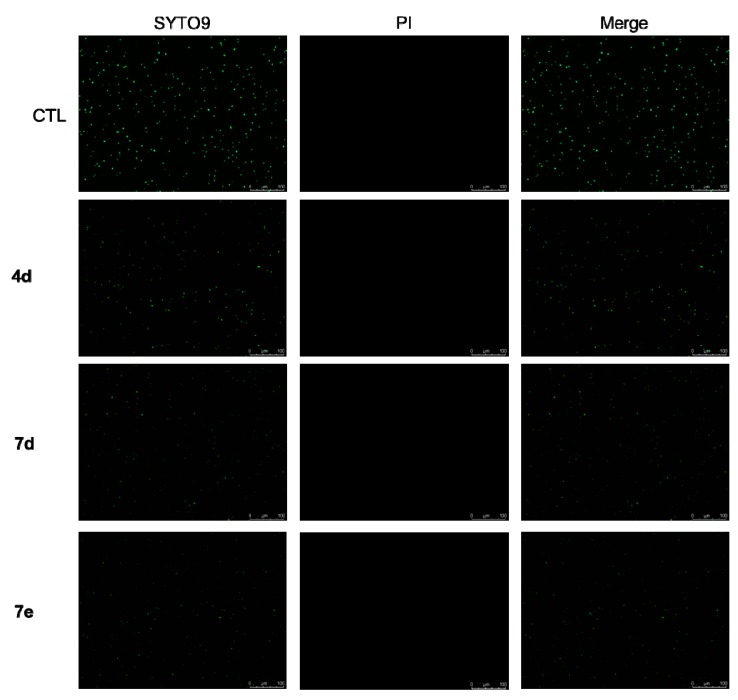
The live/dead imaging by treatment with 10 μg/mL of different compounds against planktonic MRSA. Scale bar: 100 μm

Figure 4 shows the anti-MRSA activity of compounds **4d**, **7d**, and **7e** under fluorescence microscopy. Green (SYTO9) and red (PI) signal meant live and dead MRSA, respectively. There was no/weak red signal in the MRSA, indicating that these three compounds did not affect the structure of the cell wall and cell membrane. On the other hand, the green signal was decreased after treatment of compounds **4d**, **7d**, and **7e** compared to healthy MRSA. As a result, we found that the cell wall/cell membrane of MRSA was not damaged after treatment with the compounds, but the internal DNA content was reduced. Given that these three compounds were not DNase or co-factors of DNase, we believe that the possible antibacterial activity of these three compounds was to inhibit the replication of bacterial DNA.

#### 2.2.4. Antibacterial Mechanism of Compounds **4d**, **7d**, and **7e** as DNA Polymerase Inhibitor

**Figure 5:**
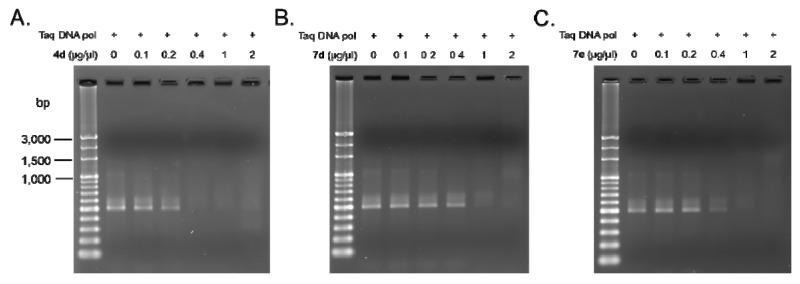
Antibacterial mechanism of compounds 4d (**A**), 7d (**B**), and 7e (**C**).

Based on the results shown in Figure 4, we considered that the antibacterial mechanism of compounds **4d**, **7d**, and **7e** might be to inhibit the synthesis of bacterial DNA. DNA polymerase is an important protein for replicating DNA in bacteria. Locatelli et al. reported that resveratrol and pterostilbene can inhibit DNA polymerases [26]. Therefore, we explored the antibacterial mechanisms of compounds **4d**, **7d**, and **7e** by DNA replication test (Figure 5). We examined the ability of compounds **4d**, **7d**, and **7e** to inhibit DNA polymerase. The PCR products were reduced by these three compounds in a dose-dependent manner, confirming the antibacterial role as a DNA polymerase inhibitor. Compound **4d** had the best DNA polymerase inhibitory activity compared to compounds **7d** and **7e**. Compound **4d** could completely inhibit the activity of DNA polymerase at a concentration of 0.4 μg/μL (Figure 5A).

### 2.3. Molecular Docking Study

**Figure 6:**
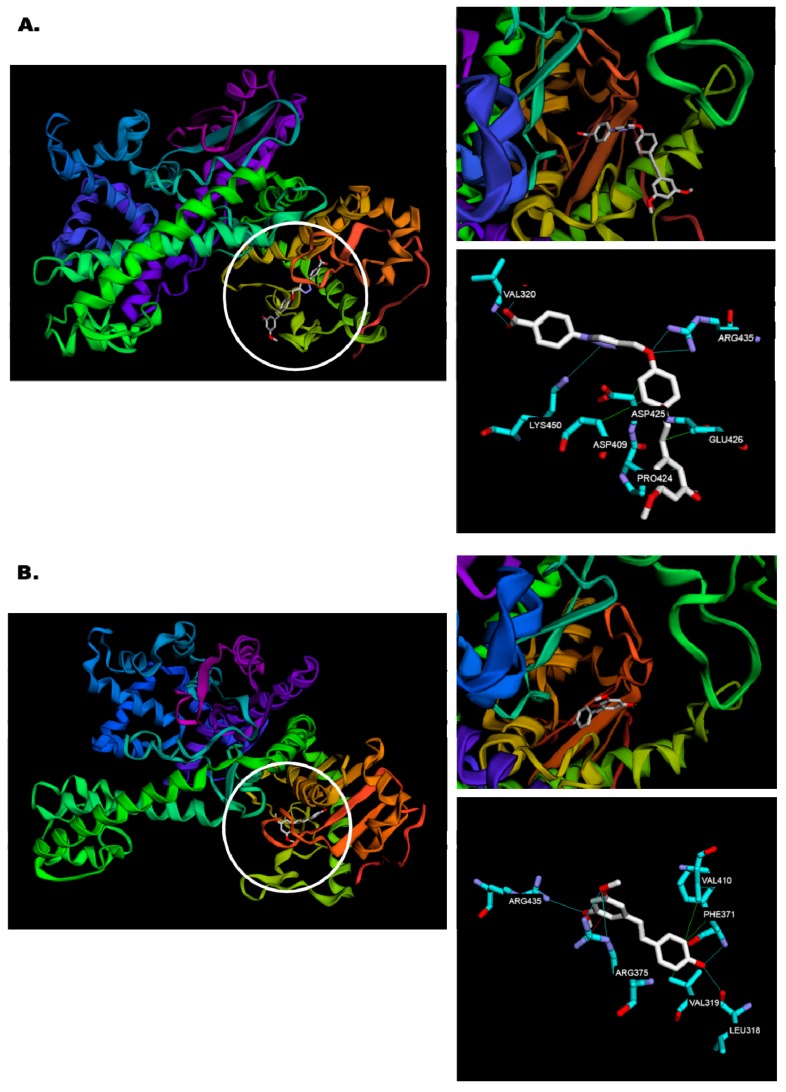
Docking pose of (**A**) compound **4d** and (**B**) pterostilbene at DNA polymerase (PDB code 4b9t). The white circle illustrated the possible active binding site of (**A**) compound **4d** and (**B**) pterostilbene on DNA polymerase. The interaction illustrated by green lines represent hydrophobic interaction, blue lines represent hydrogen bonding, and red lines represent PI–cation interaction.

In order to gain a better understanding of the binding mode and interaction [27], a molecular docking analysis of compound **4d** with DNA polymerase (PDB: 4b9t) was carried out to rationalize the biological results and compare them to pterostilbene. The docking poses of compound **4d** and pterostilbene with the lowest binding energy are shown in Figure 6. Comparing the docking poses of compound **4d** (Figure 6A) and pterostilbene (Figure 6B) with DNA polymerase, the triazolylbenzoic acid moiety of compound **4d** inserted into the same pocket as pterostilbene of DNA polymerase to form hydrogen bonds between carboxylic acid and Val320. The results could explain the structure-activity relation we inferred in that only the compounds substituted with the carboxylic acid at the terminal site showed inhibitory activity against MRSA. The triazole group and the oxygen connecting to the pterostilbene scaffold also formed hydrogen bonds with Lys450 and Arg435 individually, confirming the concept we mentioned in the structure-activity relationship that the appropriate space between triazole and carboxylic acid was needed. The pterostilbene scaffold stretched outside the pocket to form a hydrophobic interaction with Asp409, Pro424, Asp425, and Glu426 to stabilize the position between compound **4d** and DNA polymerase. The docking score of compound **4d** (−8.70 kcal/mol) was better than pterostilbene (−7.40 kcal/mol), giving good evidence that our design inserting 1,2,3-triazole into the scaffold with an appropriate spacer could successfully enhance the anti-MRSA activity.

## 3. Conclusions

We synthesized a series of novel pterostilbene derivatives using triazole as a bridge to connect a substituted aryl group or carboxylic acid. Among these compounds, compounds **4d**, **5d**, **7c**, **7d**, and **7e** exhibited moderate to potent anti-MRSA activity. Compound **4d** possessed the most potent inhibitory activity against MRSA with MIC value 1.2–2.4 μg/mL and MBC value 19.5–39 μg/mL. We also determined the structure-activity relationship that carboxylic acid should be the essential moiety at the terminal site and the appropriate spacer was needed between the triazole and carboxylic acid. Anti-MRSA mechanism studies indicated that our active compounds may inhibit MRSA by acting on DNA polymerase instead of the bacterial cell wall and cell membrane. Molecular docking studies confirmed our inference of structure-activity relationship and rationalize the biological results. In this study, the results verified that our design could successfully increase the inhibitory activity and specificity against MRSA. Compound **4d** could be used as a candidate for the development of antibacterial agents and an in depth in vivo study should be further investigated.

## 4. Materials and Methods

### 4.1. Chemistry Section

General Information. Commercial reagents were used without additional purification. Melting points were tested with an Electrothermal IA9100 (Electrothermal, Staffordshire, UK) micro-melting point apparatus and were uncorrected. NMR spectra were performed with a Varian Unity 400 MHz spectrometer using tetramethylsilane (TMS, Merck, Darmstadt, Germany) as an internal standard and DMSO-*d_6_* as solvent. Chemical shifts were presented as (ppm). Splitting patterns are described as follows: s = singlet; d = doublet; t = triplet; q = quartet; dd = double doublet; m = multiplet. Analytical thin layer chromatography (TLC) was carried out by an Art. 5554 Kieselgel 60 GF254 (Merck, Darmstadt, Germany) made by E. Merck. The spots of compounds were examined with a UV light indicator irradiated at 254 and 366 nm. Art. 7734 Kieselgel 60 GF254 (70–400 mesh, Merck, Darmstadt, Germany) produced by E. Merck was used for preparing column chromatography. The purity of compounds was determined with high performance liquid chromatography (HPLC). HPLC analysis was performed using a HITACHI Chromaster 5110 HPLC (Hitachi High-Technologies, Tokyo, Japan), fitted with a UV detector and an auto sampler. The compounds were tested on a Mightysil RP-18 GP 250-4.6 (5 mm) (Kanto Chemical, Tokyo, Japan) with mobile phase acetonitrile (for compound **4a**–**c**, **5a**–**c**, **6a**–**c**, **7a**) or methanol:0.1% formic acid = 90:10 (for compound **4d**–**g**, **5d**, **6d**, **7b**–**e**). The flow rate was 1 mL/min and the sample injection was 100 mL (1.00 mg dissolved in 0.1 mL DMSO, then diluted with methanol to 2 mL). The wavelength was set at 345 nm. Waters ZQ-4000 (Waters, Milford, MA, USA) liquid chromatography electrospray ionization mass spectrometry was used to record mass spectra.

#### 4.1.1. (E)-1,3-Dimethoxy-5-(4-(prop-2-yn-1-yloxy)styryl)benzene (**3**)

Pterostilbene (1.28 g, 5 mmol) was first reacted with propargyl bromide 80 wt.% in toluene (1.00 g, 6.72 mmol) in the condition of potassium carbonate (3.45 g, 25 mmol) and 20 mL acetone stirring rigorously in room temperature for 24 h (monitored by TLC). The mixture was first concentrated in vacuum then extracted twice by dichloromethane/water (1/1) 100 mL and the organic layer was collected, dried with magnesium sulfate, and concentrated in under reduced pressure. The crude product was purified with silica gel column chromatography, using hexane/dichloromethane as the mobile phase, to yield compound **3** as a white solid in 92%. Melting Point: 69.3–70.9 °C. Purity: 99.2%. ^1^H NMR (400 Hz, DMSO-*d_6_*): δ 7.56–7.54 (m, 2H, Ar-H), 7.23 (d, 1H, *J* = 16 Hz, CH_a_CH_b_), 7.05 (d, 1H, *J* = 16 Hz, CH_a_CH_b_), 7.01–6.99 (m, 2H, Ar-H), 6.75 (d, 2H, *J* = 2.4 Hz, Ar-H), 6.40–6.39 (m, 1H, Ar-H), 4.83 (d, 2H, *J* = 2.4 Hz, CH_2_), 3.78 (s, 6H, OCH_3_), 3.60 (s, 1H, CCH). ^13^C NMR (100 Hz, DMSO-*d_6_*): δ 160.64, 156.93, 139.36, 130.27, 128.41 (2C), 127.76 (2C), 126.58, 115.09 (2C), 104.21 (2C), 99.56, 72.23, 78.33, 55.43, 55.18 (2C). MS (ESI): *m*/*z* [M + H]^+^ 295.01.

#### 4.1.2. The Synthesis of Azide Derivatives **14**–**17** Series

The synthesis method was done according to cited references [21,22,23,24,25].

#### 4.1.3. General Procedure for the Synthesis of Compound **4**–**7** Series

Compound **3** (0.29 g, 1 mmol) and the selected azide (1.2 mmol) were dissolved in 8 mL *tert*-butanol. Sodium ascorbate (0.10 g, 0.5 mmol) and copper sulfate pentahydrate (0.13 g, 0.5 mmol) were firstly dissolved in 1 mL water, respectively, and then added dropwise into the mixture of compound **3** and the selected azide. The reaction was stirred in room temperature for 8 h (monitored by TLC). The mixture was first concentrated in vacuum then extracted twice by dichloromethane/water (1/1) 100 mL, and the organic layer was collected, dried with magnesium sulfate and concentrated in under reduced pressure. The crude product was purified with silica gel column chromatography, using dichloromethane/methanol (100/1) as the mobile phase, to obtain the target compound in 44–98%.

*(E)-4-((4-(3,5-Dimethoxystyryl)phenoxy)methyl)-1-phenyl-1H-1,2,3-triazole* (**4a**). Compound **4a** was obtained in 65% yield as a white solid. Melting Point: 146.8–148.2 °C. Purity: 97.6%. ^1^H NMR (400 Hz, DMSO-*d_6_*): δ 8.97 (s, 1H, triazole-H), 7.94–7.91 (m, 2H, Ar-H), 7.64–7.60 (m, 2H, Ar-H), 7.58–7.56 (m, 2H, Ar-H), 7.53–7.49 (m, 1H, Ar-H), 7.23 (d, 1H, *J* = 16.4 Hz, CH_a_CH_b_), 7.12–7.09 (m, 2H, Ar-H), 7.05 (d, 1H, *J* = 16.4 Hz, CH_a_CH_b_), 6.76 (d, 2H, *J* = 2.4 Hz, Ar-H), 6.40–6.39 (m, 1H, Ar-H), 5.27 (s, 2H, CH_2_), 3.78 (s, 6H, OCH_3_). ^13^C NMR (100 Hz, DMSO-*d_6_*): δ 160.64, 157.74, 143.81, 139.38, 136.55, 130.01 (2C), 129.92, 128.77, 128.46 (2C), 127.87 (2C), 126.44, 122.91, 120.16 (2C), 115.01 (2C), 104.21 (2C), 99.51, 61.06, 55.18 (2C). MS (ESI): *m*/*z* [M + H]^+^ 414.02.

*(E)-4-((4-(3,5-Dimethoxystyryl)phenoxy)methyl)-1-(4-fluorophenyl)-1H-1,2,3-triazole* (**4b**). Compound **4b** was obtained in 82% yield as a white solid. Melting Point: 144.9–146.8 °C. Purity: 97.8%. ^1^H NMR (400 Hz, DMSO-*d_6_*): δ 8.96 (s, 1H, triazole-H), 7.99–7.96 (m, 2H, Ar-H), 7.58–7.56 (m, 2H, Ar-H), 7.50–7.45 (m, 2H, Ar-H), 7.23 (d, 1H, *J* = 16.4 Hz, CH_a_CH_b_), 7.11–7.08 (m, 2H, Ar-H), 7.05 (d, 1H, *J* = 16.4 Hz, CH_a_CH_b_), 6.75 (d, 2H, *J* = 2.4 Hz, Ar-H), 6.40–6.39 (m, 1H, Ar-H), 5.27 (s, 2H, CH_2_), 3.78 (s, 6H, OCH_3_). ^13^C NMR (100 Hz, DMSO-*d_6_*): δ 160.70 (^1^*J*_CF_ = 244.1 Hz), 160.64, 157.73, 143.84, 139.38, 133.11, 130.03, 128.46 (2C), 127.87 (2C), 126.44, 123.16, 122.55 (2C, ^3^*J*_CF_ = 8.4 Hz), 116.76 (2C, ^2^*J*_CF_ = 22.7 Hz), 115.01 (2C), 104.21 (2C), 99.51, 61.04, 55.18 (2C). MS (ESI): *m*/*z* [M + H]^+^ 432.00.

*(E)-4-((4-(3,5-Dimethoxystyryl)phenoxy)methyl)-1-(4-methoxyphenyl)-1H-1,2,3-triazole* (**4c**). Compound **4c** was obtained in 92% yield as an orange solid. Melting Point: 157.9–159.0 °C. Purity: 96.7%. ^1^H NMR (400 Hz, DMSO-*d_6_*): δ 8.86 (s, 1H, triazole-H), 7.84–7.80 (m, 2H, Ar-H), 7.58–7.55 (m, 2H, Ar-H), 7.23 (d, 1H, *J* = 16.4 Hz, CH_a_CH_b_), 7.17–7.13 (m, 2H, Ar-H), 7.12–7.08 (m, 2H, Ar-H), 7.05 (d, 1H, *J* = 16.4 Hz, CH_a_CH_b_), 6.75 (d, 2H, *J* = 2 Hz, Ar-H), 6.40–6.39 (m, 1H, Ar-H), 5.25 (s, 2H, CH_2_), 3.84 (s, 3H, OCH_3_), 3.78 (s, 6H, OCH_3_). ^13^C NMR (100 Hz, DMSO-*d_6_*): δ 160.65, 159.34, 157.78, 143.57, 139.41, 130.00 (2C), 128.49 (2C), 127.89 (2C), 126.43, 122.90, 121.84, 115.02 (2C), 114.91 (2C), 104.22 (2C), 99.52, 61.09, 55.58, 55.19 (2C). MS (ESI): *m*/*z* [M + H]^+^ 444.02.

*(E)-4-(4-((4-(3,5-Dimethoxystyryl)phenoxy)methyl(-1H-1,2,3-triazol-1-yl)benzoic acid* (**4d**). Compound **4d** was obtained in 82% yield as a purple solid. Melting Point: 235.4–241.7 °C. Purity: 97.5%. ^1^H NMR (400 Hz, DMSO-*d_6_*): δ 9.08 (s, 1H, triazole-H), 8.16–8.13 (m, 2H, Ar-H), 8.07–8.05 (m, 2H, Ar-H), 7.58–7.56 (m, 2H, Ar-H), 7.23 (d, 1H, *J* = 16.4 Hz, CH_a_CH_b_), 7.12–7.10 (m, 2H, Ar-H), 7.05 (d, 1H, *J* = 16.4 Hz, CH_a_CH_b_), 6.75 (d, 2H, *J* = 2 Hz, Ar-H), 6.40–6.39 (m, 1H, Ar-H), 5.29 (s, 2H, CH_2_), 3.78 (s, 6H, OCH_3_). ^13^C NMR (100 Hz, DMSO-*d_6_*): δ 166.54, 160.64, 157.72, 144.13, 139.39, 139.16, 131.03 (2C), 130.06, 128.46 (2C), 127.88 (2C), 126.47, 123.02, 119.80 (2C), 119.13, 115.03 (2C), 104.21 (2C), 99.53, 61.02, 55.18 (2C). MS (ESI): *m*/*z* [M + H]^+^ 457.98.

*(E)-4-(4-((4-(3,5-Dimethoxystyryl)phenoxy)methyl(-1H-1,2,3-triazol-1-yl)benzenesulfonamide* (**4e**). Compound **4e** was obtained in 91% yield as a light yellow solid. Melting Point: 228.2–230.1 °C. Purity: 96.1%. ^1^H NMR (400 Hz, DMSO-*d_6_*): δ 9.09 (s, 1H, triazole-H), 8.17–8.15 (m, 2H, Ar-H), 8.05–8.03 (m, 2H, Ar-H), 7.58–7.56 (m, 4H, Ar-H, NH_2_), 7.23 (d, 1H, *J* = 16.4 Hz, CH_a_CH_b_), 7.12–7.10 (m, 2H, Ar-H), 7.07–7.03 (d, 1H, *J* = 16.4 Hz, CH_a_CH_b_), 6.75 (d, 2H, *J* = 1.6 Hz, Ar-H), 6.40 (brs, 1H, Ar-H), 5.29 (s, 2H, CH_2_), 3.78 (s, 6H, OCH_3_). ^13^C NMR (100 Hz, DMSO-*d_6_*): δ 160.64, 157.71, 144.22, 143.92, 139.38, 138.54, 130.08, 128.46 (2C), 127.89 (2C), 127.52 (2C), 126.48, 123.14, 120.42 (2C), 115.02 (2C), 104.22 (2C), 99.52, 61.01, 55.18 (2C). MS (ESI): *m*/*z* [M + H]^+^ 492.99.

*(E)-2-(4-(4-((4-(3,5-Dimethoxystyryl)phenoxy)methyl)-1H-1,2,3-triazol-1-yl(phenyl)acetic acid* (**4f**). Compound **4f** was obtained in 70% yield as a white solid. Melting Point: 147.0–148.7 °C. Purity: 98.7%. ^1^H NMR (400 Hz, DMSO-*d_6_*): δ 8.94 (s, 1H, triazole-H), 7.86–7.84 (m, 2H, Ar-H), 7.58–7.55 (m, 2H, Ar-H), 7.50–7.48 (m, 2H, Ar-H), 7.23 (d, 1H, *J* = 16.4 Hz, CH_a_CH_b_), 7.11–7.09 (m, 2H, Ar-H), 7.05 (d, 1H, *J* = 16.4 Hz, CH_a_CH_b_), 6.75 (d, 2H, *J* = 2.4 Hz, Ar-H), 6.40–6.39 (m, 1H, Ar-H), 5.26 (s, 2H, CH_2_), 3.78 (s, 6H, OCH_3_), 3.67 (s, 2H, CH_2_COOH). ^13^C NMR (100 Hz, DMSO-*d_6_*): δ 160.66, 157.78, 143.77, 139.41, 136.33, 135.12, 130.90 (2C), 130.03, 128.49 (2C), 127.90 (2C), 126.46, 122.91, 120.01 (2C), 115.03 (2C), 104.23 (2C), 99.54, 61.08, 55.20 (2C). MS (ESI): *m*/*z* [M + H]^+^ 472.01.

*(E)-4-(4-((4-(3,5-Dimethoxystyryl)phenoxy)methyl(-1H-1,2,3-triazol-1-yl)-2-hydroxybenzoic acid* (**4g**). Compound **4g** was obtained in 59% yield as a white solid. Melting Point: 214.4–216.2 °C. Purity: 98.6%. ^1^H NMR (400 Hz, DMSO-*d_6_*): δ 10.07 (s, 1H, OH), 8.91 (s, 1H, triazole-H), 7.57–7.55 (m, 2H, Ar-H), 7.41–7.37 (m, 1H, Ar-H), 7.32–7.30 (m, 1H, Ar-H), 7.23 (d, 1H, *J* = 16.4 Hz, CH_a_CH_b_), 7.11–7.09 (m, 2H, Ar-H), 7.05 (d, 1H, *J* = 16.4 Hz, CH_a_CH_b_), 6.91–6.88 (m, 1H, Ar-H), 6.75 (d, 2H, *J* = 2.4 Hz, Ar-H), 6.40–6.39 (m, 1H, Ar-H), 5.25 (s, 2H, CH_2_), 3.78 (s, 6H, OCH_3_). ^13^C NMR (100 Hz, DMSO-*d_6_*): δ 160.63, 158.47, 157.73, 143.65, 139.38, 137.53, 130.79 (2C), 130.00, 128.47 (2C), 127.86 (2C), 126.43, 122.87, 115.71, 115.01 (2C), 110.49, 107.04, 104.20 (2C), 99.51, 61.01, 55.17 (2C). MS (ESI): *m*/*z* [M + H]^+^ 474.03.

*(E)-1-benzyl-4-((4-(3,5-Dimethoxystyryl)phenoxy(methyl)-1H-1,2,3-triazole* (**5a**). Compound **5a** was obtained in 94% yield as a white solid. Melting Point: 127.8–129.0 °C. Purity: 99.8%. ^1^H NMR (400 Hz, DMSO-*d_6_*): δ 8.03 (s, 1H, triazole-H), 7.56–7.52 (m, 2H, Ar-H), 7.41–7.31 (m, 5H, Ar-H), 7.22 (d, 1H, *J* = 16.4 Hz, CH_a_CH_b_), 7.06–7.02 (m, 3H, Ar-H, CH_a_CH_b_), 6.74 (d, 2H, *J* = 2.4 Hz, Ar-H), 6.40–6.39 (m, 1H, Ar-H), 5.62 (s, 2H, CH_2_Ar), 5.16 (s, 2H, CH_2_), 3.77 (s, 6H, OCH_3_). ^13^C NMR (100 Hz, DMSO-*d_6_*): δ 160.64, 157.79, 142.94, 139.41, 136.03, 129.89, 128.80 (2C), 128.49, 128.19 (2C), 127.98 (2C), 127.83 (2C), 126.36, 124.75, 114.97 (2C), 104.19 (2C), 99.50, 61.10, 55.19 (2C), 52.84. MS (ESI): *m*/*z* [M + H]^+^ 428.04.

*(E)-4-((4-(3,5-Dimethoxystyryl)phenoxy(methyl)-1-(4-fluorobenzyl)-1H-1,2,3-triazole* (**5b**). Compound **5b** was obtained in 83% yield as a white solid. Melting Point: 141.4–143.0 °C. Purity: 99.9%. ^1^H NMR (400 Hz, DMSO-*d_6_*): δ 8.03 (s, 1H, triazole-H), 7.55–7.53 (m, 2H, Ar-H), 7.43–7.39 (m, 2H, Ar-H), 7.24–7.20 (m, 3H, Ar-H, CH_a_CH_b_), 7.06–7.02 (m, 3H, Ar-H, CH_a_CH_b_), 6.74 (d, 2H, *J* = 2 Hz, Ar-H), 6.40–6.39 (m, 1H, Ar-H), 5.61 (s, 2H, CH_2_Ar), 5.16 (s, 2H, CH_2_), 3.78 (s, 6H, OCH_3_). ^13^C NMR (100 Hz, DMSO-*d_6_*): δ 161.91(^1^*J*_CF_ = 242.7 Hz), 160.64, 157.79, 142.97, 139.41, 132.29 (^4^*J*_CF_ = 3.4 Hz), 130.34 (2C, ^3^*J*_CF_ = 8.4 Hz), 129.90, 128.49 (2C), 127.83 (2C), 126.38, 124.67, 115.64 (2C, ^2^*J*_CF_ = 21.4 Hz), 114.97 (2C), 104.20 (2C), 99.50, 61.09, 55.19 (2C), 52.04. MS (ESI): *m*/*z* [M + H]^+^ 446.03.

*(E)-4-((4-(3,5-Dimethoxystyryl)phenoxy(methyl)-1-(4-methoxybenzyl)-1H-1,2,3-triazole* (**5c**). Compound **5c** was obtained in 86% yield as a white solid. Melting Point: 145.1–147.2 °C. Purity: 99.9%. ^1^H NMR (400 Hz, DMSO-*d_6_*): δ 8.24 (s, 1H, triazole-H), 7.54–7.52 (m, 2H, Ar-H), 7.31–7.29 (m, 2H, Ar-H), 7.22 (d, 1H, *J* = 16.4 Hz, CH_a_CH_b_), 7.05–7.01 (m, 3H, Ar-H, CH_a_CH_b_), 6.95–6.92 (m, 2H, Ar-H), 6.74 (d, 2H, *J* = 2.4 Hz, Ar-H), 6.40–6.39 (m, 1H, Ar-H), 5.53 (s, 2H, CH_2_Ar), 5.15 (s, 2H, CH_2_), 3.78 (s, 6H, OCH_3_), 3.73 (s, 3H, OCH_3_). ^13^C NMR (100 Hz, DMSO-*d_6_*): δ 160.63, 159.14, 157.79, 142.87, 139.39, 129.87, 129.62 (2C), 128.48 (2C), 127.90, 127.81 (2C), 126.36, 124.37, 114.96 (2C), 114.12 (2C), 104.19 (2C), 99.50, 61.10, 55.17 (2C), 55.12, 52.38. MS (ESI): *m*/*z* [M + H]^+^ 458.05.

*(E)-4-((4-((4-(3,5-Dimethoxystyryl)phenoxy)methyl)-1H-1,2,3-triazol-1-yl(methyl)-benzoic acid* (**5d**). Compound **5d** was obtained in 65% yield as a white solid. Melting Point: 186.3–188.5 °C. Purity: 96.6%. ^1^H NMR (400 Hz, DMSO-*d_6_*): δ 8.34 (s, 1H, triazole-H), 7.96–7.94 (m, 2H, Ar-H), 7.55–7.53 (m, 2H, Ar-H), 7.41–7.39 (m, 2H, Ar-H), 7.22 (d, 1H, *J* = 16 Hz, CH_a_CH_b_), 7.06–7.02 (m, 3H, Ar-H, CH_a_CH_b_), 6.75 (d, 2H, *J* = 2 Hz, Ar-H), 6.40–6.39 (m, 1H, Ar-H), 5.72 (s, 2H, CH_2_Ar), 5.18 (s, 2H, CH_2_), 3.78 (s, 6H, OCH_3_). ^13^C NMR (100 Hz, DMSO-*d_6_*): δ 166.95, 160.65, 157.79, 143.03, 140.76, 139.41, 130.58, 129.92, 129.80 (2C), 128.49 (2C), 127.98 (2C), 127.85 (2C), 126.39, 125.02, 114.99 (2C), 104.20 (2C), 99.51, 61.10, 55.19 (2C), 52.41. MS (ESI): *m*/*z* [M + H]^+^ 472.01.

*(E)-2-(4-((4-(3,5-Dimethoxystyryl)phenoxy)methyl(-1H-1,2,3-triazol-1-yl)-1-phenylethan-1-one* (**6a**). Compound **6a** was obtained in 90% yield as a light yellow solid. Melting Point: 135.9–138.0 °C. Purity: 99.5%. ^1^H NMR (400 Hz, DMSO-*d_6_*): δ 8.22 (s, 1H, triazole-H), 8.10–8.07 (m, 2H, Ar-H), 7.77–7.73 (m, 1H, Ar-H), 7.64–7.60 (m, 2H, Ar-H), 7.57–7.55 (m, 2H, Ar-H), 7.24 (d, 1H, *J* = 16.4 Hz, CH_a_CH_b_), 7.11–7.03 (m, 3H, Ar-H, CH_a_CH_b_), 6.75 (d, 2H, *J* = 2.4 Hz, Ar-H), 6.40–6.39 (m, 1H, Ar-H), 6.23 (s, 2H, CH_2_CO), 5.23 (s, 2H, CH_2_), 3.78 (s, 6H, OCH_3_). ^13^C NMR (100 Hz, DMSO-*d_6_*): δ 192.18, 160.65, 157.86, 142.54, 139.42, 134.27, 134.10 (2C), 129.88, 129.01 (2C), 128.52, 128.20 (2C), 127.87 (2C), 126.35, 114.98 (2C), 104.20 (2C), 99.50, 61.10, 55.92, 55.19 (2C). MS (ESI): *m*/*z* [M + H]^+^ 456.03.

*(E)-2-(4-((4-(3,5-Dimethoxystyryl)phenoxy)methyl(-1H-1,2,3-triazol-1-yl)-1-(4-fluorophenyl)ethan-1-one* (**6b**). Compound **6b** was obtained in 44% yield as a light yellow solid. Melting Point: 164.9–166.5 °C. Purity: 99.9%. ^1^H NMR (400 Hz, DMSO-*d_6_*): δ 8.21 (s, 1H, triazole-H), 8.19–8.15 (m, 2H, Ar-H), 7.57–7.55 (m, 2H, Ar-H), 7.48–7.44 (m, 2H, Ar-H), 7.23 (d, 1H, *J* = 16.4 Hz, CH_a_CH_b_), 7.10–7.03 (m, 3H, Ar-H, CH_a_CH_b_), 6.75 (d, 2H, *J* = 2.4 Hz, Ar-H), 6.40–6.39 (m, 1H, Ar-H), 6.22 (s, 2H, CH_2_CO), 5.23 (s, 2H, CH_2_), 3.78 (s, 6H, OCH_3_). ^13^C NMR (100 Hz, DMSO-*d_6_*): δ 190.88, 165.59 (^1^*J*_CF_ = 251.2 Hz), 160.65, 157.86, 142.57, 139.42, 131.33 (2C, ^3^*J*_CF_ = 9.5 Hz), 130.91 (^4^*J*_CF_ = 3.0 Hz), 129.89, 128.52 (2C), 127.87 (2C), 126.36, 126.33, 116.13 (2C, ^2^*J*_CF_ = 21.7 Hz), 114.98 (2C), 104.20 (2C), 99.50, 61.10, 55.86, 55.19 (2C). MS (ESI): *m*/*z* [M + H]^+^ 474.03.

*(E)-2-(4-((4-(3,5-Dimethoxystyryl)phenoxy)methyl(-1H-1,2,3-triazol-1-yl)-1-(4-methoxyphenyl)ethan-1-one* (**6c**). Compound **6c** was obtained in 50% yield as a white solid. Melting Point: 146.2–148.1 °C. Purity: 99.8%. ^1^H NMR (400 Hz, DMSO-*d_6_*): δ 8.20 (s, 1H, triazole-H), 8.07–8.05 (m, 2H, Ar-H), 7.57–7.55 (m, 2H, Ar-H), 7.23 (d, 1H, *J* = 16.4 Hz, CH_a_CH_b_), 7.14–7.12 (m, 2H, Ar-H), 7.10–7.03 (m, 3H, Ar-H, CH_a_CH_b_), 6.75 (d, 2H, *J* = 2.0 Hz, Ar-H), 6.40–6.39 (m, 1H, Ar-H), 6.15 (s, 2H, CH_2_CO), 5.23 (s, 2H, CH_2_), 3.88 (s, 3H, OCH_3_), 3.78 (s, 6H, OCH_3_). ^13^C NMR (100 Hz, DMSO-*d_6_*): δ 190.40, 163.92, 160.64, 157.87, 142.47, 139.42, 130.61 (2C), 129.87, 128.52 (2C), 127.87 (2C), 126.96, 126.39, 126.36, 114.98 (2C), 114.24 (2C), 104.20 (2C), 99.51, 61.11, 55.71, 55.55, 55.19 (2C). MS (ESI): *m*/*z* [M + H]^+^ 485.98.

*(E)-4-(2-(4-((4-(3,5-Dimethoxystyryl)phenoxy)methyl)-1H-1,2,3-triazol-1-yl(acetyl)benzoic acid* (**6d**). Compound **6d** was obtained in 72% yield as a light purple solid. Melting Point: 251.2–253.0 °C. Purity: 96%. ^1^H NMR (400 Hz, DMSO-*d_6_*): δ 8.25–8.14 (m, 5H, 4H of Ar-H, triazole-H), 7.57–7.53 (m, 2H, Ar-H), 7.23 (d, 1H, *J* = 16.4 Hz, CH_a_CH_b_), 7.10–7.03 (m, 3H, Ar-H, CH_a_CH_b_), 6.75 (d, 2H, *J* = 2.0 Hz, Ar-H), 6.39 (brs, 1H, Ar-H), 6.26 (s, 2H, CH_2_CO), 5.24 (s, 2H, CH_2_), 3.78 (s, 6H, OCH_3_). ^13^C NMR (100 Hz, DMSO-*d_6_*): δ 192.03, 160.62 (2C), 157.83, 142.62, 139.39, 137.18, 129.87 (2C), 128.49 (2C), 127.83 (2C), 126.35 (2C), 126.28, 114.96 (2C), 104.18 (2C), 99.49, 61.08, 56.13, 55.16 (2C). MS (ESI): *m*/*z [M + H]^+^ 500.01.*

*Ethyl (E)-2-(4-((4-(3,5-dimethoxystyryl)phenoxy)methyl(-1H-1,2,3-triazol-1-yl)acetate* (**7a**). Compound **7a** was obtained in 98% yield as a white solid. Melting Point: 163.0–163.7 °C. Purity: 99.8%. ^1^H NMR (400 Hz, DMSO-*d_6_*): δ 8.24 (s, 1H, triazole-H), 7.56–7.54 (m, 2H, Ar-H), 7.22 (d, 1H, *J* = 16.4 Hz, CH_a_CH_b_), 7.07–7.02 (m, 3H, Ar-H, CH_a_CH_b_), 6.75 (d, 2H, *J* = 2.4 Hz, Ar-H), 6.40–6.39 (m, 1H, Ar-H), 5.42 (s, 2H, CH_2_CO), 5.20 (s, 2H, CH_2_), 4.18 (q, 2H, *J* = 7.2 Hz, OCH_2_CH_3_), 3.78 (s, 6H, OCH_3_), 1.22 (t, 3H, *J* = 7.2 Hz, OCH_2_CH_3_). ^13^C NMR (100 Hz, DMSO-*d_6_*): δ 167.23, 160.64, 157.79, 142.67, 139.41, 129.91, 128.49 (2C), 127.84 (2C), 126.38, 126.03, 114.97 (2C), 104.20 (2C), 99.51, 61.52, 61.01, 55.19 (2C), 50.40, 13.97. MS (ESI): *m*/*z* [M + H]^+^ 424.02.

*(E)-2-(4-((4-(3,5-Dimethoxystyryl)phenoxy)methyl(-1H-1,2,3-triazol-1-yl)acetic acid* (**7b**). Compound **7a** was refluxed in 1,4-dioxane with few drops of 6 M sodium hydroxide for 2 h to obtained crude products. The mixture was concentrated in vacuum and then recrystallized in ethanol to yield compound **7b** in 79% as a white solid. Melting Point: 193.4–194.8 °C. Purity: 99.6%. ^1^H NMR (400 Hz, DMSO-*d_6_*): δ 8.23 (s, 1H, triazole-H), 7.56–7.54 (m, 2H, Ar-H), 7.22 (d, 1H, *J* = 16.4 Hz, CH_a_CH_b_), 7.08–7.02 (m, 3H, Ar-H, CH_a_CH_b_), 6.75 (d, 2H, *J* = 2.4 Hz, Ar-H), 6.40–6.39 (m, 1H, Ar-H), 5.30 (s, 2H, CH_2_CO), 5.19 (s, 2H, CH_2_), 3.78 (s, 6H, OCH_3_). ^13^C NMR (100 Hz, DMSO-*d_6_*): δ 168.62, 160.65, 157.84, 142.53, 139.42, 129.89, 128.51 (2C), 127.85 (2C), 126.37, 126.02, 114.96 (2C), 104.21 (2C), 99.51, 61.04, 55.19 (2C), 50.52. MS (ESI): *m*/*z* [M + H]^+^ 396.03.

*(E)-3-(4-((4-(3,5-Dimethoxystyryl)phenoxy)methyl]-1H-1,2,3-triazol-1-yl)propanoic acid* (**7c**). Compound **7c** was obtained in 67% yield as a brown solid. Melting Point: 193.4–194.8 °C. Purity: 99.3%. ^1^H NMR (400 Hz, DMSO-*d_6_*): δ 8.23 (s, 1H, triazole-H), 7.55–7.53 (m, 2H, Ar-H), 7.22 (d, 1H, *J* = 16.4 Hz, CH_a_CH_b_), 7.06–7.02 (m, 3H, Ar-H, CH_a_CH_b_), 6.75 (d, 2H, *J* = 2.4 Hz, Ar-H), 6.40–6.39 (m, 1H, Ar-H), 5.19 (s, 2H, CH_2_), 4.59 (s, 2H, CH_2_CO), 3.78 (s, 6H, OCH_3_), 2.94 (s, 2H, CH_2_CH_2_). ^13^C NMR (100 Hz, DMSO-*d_6_*): δ 160.63, 157.83, 142.47, 139.40, 129.87, 128.49 (2C), 127.83 (2C), 126.35, 124.78, 114.93 (2C), 104.19 (2C), 99.50, 61.09, 55.17 (2C), 45.88, 18.55. MS (ESI): *m*/*z* [M + H]^+^ 410.05.

*(E)-5-(4-((4-(3,5-Dimethoxystyryl)phenoxy)methyl]-1H-1,2,3-triazol-1-yl)pentanoic acid* (**7d**). Compound **7d** was obtained in 90% yield as a brown solid. Melting Point: 152.7–154.3 °C. Purity: 99.3%. ^1^H NMR (400 Hz, DMSO-*d_6_*): δ 8.24 (s, 1H, triazole-H), 7.55–7.53 (m, 2H, Ar-H), 7.22 (d, 1H, *J* = 16.8 Hz, CH_a_CH_b_), 7.06–7.02 (m, 3H, Ar-H, CH_a_CH_b_), 6.75 (d, 2H, *J* = 2.4 Hz, Ar-H), 6.40–6.39 (m, 1H, Ar-H), 5.16 (s, 2H, CH_2_), 4.38 (t, 2H, *J* = 7.2 Hz, CH_2_COOH), 3.78 (s, 6H, OCH_3_), 2.26 (t, 2H, *J* = 7.2 Hz, triazole-CH_2_CH_2_), 1.84 (p, 2H, *J* = 7.2 Hz, CH_2_CH_2_), 1.46 (p, 2H, *J* = 7.2 Hz, CH_2_CH_2_). ^13^C NMR (100 Hz, DMSO-*d_6_*): δ 174.17, 160.64, 157.83, 142.53, 139.40, 129.87, 128.49 (2C), 127.82 (2C), 126.36, 124.45, 114.97 (2C), 104.20 (2C), 99.50, 61.19, 55.17 (2C), 49.08, 32.88, 29.14, 21.42. MS (ESI): *m*/*z* [M + H]^+^ 438.04.

*(E)-6-(4-((4-(3,5-Dimethoxystyryl)phenoxy)methyl]-1H-1,2,3-triazol-1-yl)hexanoic acid* (**7e**). Compound **7e** was obtained in 89% yield as a light yellow solid. Melting Point: 135.2–137.9 °C. Purity: 99.0%. ^1^H NMR (400 Hz, DMSO-*d_6_*): δ 8.24 (s, 1H, triazole-H), 7.55–7.53 (m, 2H, Ar-H), 7.22 (d, 1H, *J* = 16.4 Hz, CH_a_CH_b_), 7.06–7.02 (m, 3H, Ar-H, CH_a_CH_b_), 6.75 (d, 2H, *J* = 2.4 Hz, Ar-H), 6.40–6.39 (m, 1H, Ar-H), 5.16 (s, 2H, CH_2_), 4.36 (t, 2H, *J* = 7.2 Hz, CH_2_COOH), 3.78 (s, 6H, OCH_3_), 2.20 (t, 2H, *J* = 7.2 Hz, triazole-CH_2_CH_2_), 1.82 (p, 2H, *J* = 7.2 Hz, CH_2_CH_2_), 1.52 (p, 2H, *J* = 7.2 Hz, CH_2_CH_2_), 1.28–1.22 (m, 2H, CH_2_CH_2_). ^13^C NMR (100 Hz, DMSO-*d_6_*): δ 174.38, 160.65, 157.83, 142.52, 139.41, 129.88, 128.50 (2C), 127.84 (2C), 126.37, 124.44, 114.99 (2C), 104.21 (2C), 99.51, 61.20, 55.19 (2C), 49.25, 33.46, 29.47, 25.42, 23.87. MS (ESI): *m*/*z* [M + H]^+^ 452.07.

### 4.2. Biological Activity

#### 4.2.1. Bacterial Strains and Culture Conditions

The clinical vancomycin-intermediate *S. aureus* isolates (VISA, KV-1) was provided by Kaohsiung Medical University Hospital and used as received. Methicillin-resistant *S. aureus* (MRSA, ATCC33591) was purchased from American Type Culture Collection (ATCC, Manassas, VA, USA). All bacterium strains were grown in tryptic soy broth (TSB) medium at 37 °C and 150 rpm.

#### 4.2.2. Agar Diffusion Assay

The assay was performed by inoculated MRSA or VISA (OD_600_ = 0.7) in a 0.75% agar medium. Mixtures of 5 mL bacteria–agar medium were poured into 6 cm culture dishes at room temperature for 15 min, and the compounds (5, 2.5, 1.25, 0.625, and 0.312 mg/mL) with a volume of 10 μL were pipetted on the agar. The plates were incubated at 37 °C for 12 h. Subsequently, the diameters of the inhibition zones were measured [28].

#### 4.2.3. Minimum Inhibitory Concentration (MIC) and Minimal Bactericidal Concentration (MBC) Determination

A broth twofold serial dilution method was used to determine the MIC of cationic surfactant micelles. Overnight cultures of the *S. aureus* were diluted in TSB to achieve optical density at 600 nm (OD_600_) = 0.01 (~2 × 10^6^ cfu/mL). The bacterial cell populations were then exposed to several dilutions of different compounds ranging from 0.61 to 1250 μg/mL with TSB in 96-well culture plates and incubated at 37 °C for 20 h. The Epoch^2®^ ELISA reader (BioTek^®^, Winooski, VT, USA) was used to detect OD600 nm absorbance value. MIC was defined as the lowest concentration of the compound being able to inhibit visible growth of the bacteria (OD600 < 0.09). Wells containing broth only and bacteria only were used as controls. 

For MBC assays, the bacterial suspensions were diluted in PBS solution and plated on TSB plates. The plates were incubated for 20 h at 37 °C and the CFU were counted. MBC was defined as the lowest concentration of drug that killed ≥99.9% initial inoculums after 20 h of incubation at 37 °C. Broth medium alone and bacterial cultures were used as negative and positive controls, respectively [29,30].

#### 4.2.4. Cell Viability Assay

Cell viability was evaluated using a TOOLS Cell Counting (CCK-8) kit (Tools, Taipei, Taiwan). HaCaT cells (2 × 10^4^) were plated in 96 well culture plates and treated with compounds for 24 h viability measurement. Experimental procedures were performed according to the manufacturer’s protocol.

#### 4.2.5. Bacterial Viability Determined by Fluorescence Microscopy

Overnight cultures of the MRSA were diluted in TSB to achieve optical density at 600 nm (OD_600_) = 0.1. A 1000 μL aliquot of each tested sample was transferred into a sterile 1.5 mL Eppendorf and the pellet was harvested by centrifugation for 3 min at 12,000 rpm. The pellet was re-suspended in 1000 μL culture medium with 10 μg/mL of the compound at 37 °C with 150 rpm for 4 h. The samples were stained with a Live/Dead BacLight^®^ kit (Thermo Fisher Scientific, Waltham, MA, USA) for 15 min. The samples were monitored by Leica DMi8 fluorescence microscopy [31].

#### 4.2.6. Anti-Taq DNA Polymerase Activity

Polymerase chain reaction (PCR) was performed with 50 μL volume of the following concentrations: 100 ng MRSA genomic DNA as template, 0.4 μM primer 1 (isaB-F: 5′-atgaataaaaccagtaaagtttg-3′), 0.4 μM primer 2 (isaB-R: 5′-ttatttacttgttttgtatgg-3′), 1 x YEA Taq PCR reaction buffer, 2.5 mM each dNTP, 2.5 U YEA Taq DNA polymerase, and the different compounds (0.1 to 2 μg/μL). The PCR reaction was performed in the instrument at 95 °C for 1 min, 30 cycles of 10 sec at 95 °C, 30 s at 56 °C, and 72 °C for 1 min to generate 528 bp amplicons; results were analyzed by electrophoresis on a 1.2% agarose gel [32].

### 4.3. Molecular Docking Study

The crystal structures of DNA polymerase (PDB ID: 4B9T) were downloaded from the RCSB Protein Data Bank. The 3D conformation of hit compound 4d was produced by ChemBio 3D Ultra 14.0 (PerkinElmer, Waltham, MA, USA) Calculations were carried out with Blind Docking Server (UCAM, Guadalupe, Murcia, Spain), available at: http://bio-hpc.eu/software/blind-docking-server/. The “blind docking” approach was used for the docking of the small molecule to the targets, which was done without a priori knowledge of the location of the binding site by the system [33]. Visual representation of molecules was created with 3Dmol by Nicholas Rego and David Koes [34].

## Figures and Tables

**Table 1:** Zone of inhibition of methicillin-resistant *Staphylococcus aureus* (MRSA) and vancomycin-intermediate *S. aureus* (VISA) measured by agar diffusion assay.

Compounds	Strains	Inhibition Zone (mm)				
		500 (μg/mL)	2500 (μg/mL)	1250 (μg/mL)	625 (μg/mL)	312 (μg/mL)
**4d**	MRSA	12.18 ± 1.36	10.15 ± 0.26	10.59 ± 0.60	11.24 ± 0.55	11.27 ± 0.34
	VISA	11.64 ± 0.68	10.23 ± 0.13	11.83 ± 1.25	14.28 ± 0.14	12.98 ± 0.50
**5d**	MRSA	10.46 ± 0.97	9.79 ± 0.28	10.91 ± 0.47	10.41 ± 0.33	8.29 ± 0.18
	VISA	13.55 ± 1.20	13.68 ± 3.37	16.68 ± 1.06	15.10 ± 0.30	11.91 ± 0.24
**7c**	MRSA	13.06 ± 1.28	8.78 ± 1.82	7.90 ± 1.43	6.11 ± 0.47	0.57 ± 0.98
	VISA	15.75 ± 0.78	10.85 ± 1.13	8.36 ± 0.06	2.76 ± 4.79	0
**7d**	MRSA	21.43 ± 0.16	19.79 ± 0.11	15.295 ± 0.16	11.16 ± 0.17	9.06 ± 0.01
	VISA	16.94 ± 0.94	14.04 ± 0.70	11.78 ± 2.39	6.25 ± 5.55	0
**7e**	MRSA	20.88 ± 0.04	19.59 ± 0.34	14.96 ± 0.17	10.11 ± 0.04	8.72 ± 0.08
	VISA	17.19 ± 0.99	14.89 ± 0.75	11.75 ± 1.51	6.93 ± 6.46	0

**Table 2:** The minimum inhibitory concentration (MIC) and minimum bactericidal concentration (MBC) values of MRSA and VISA after treatment with different compounds.

Compounds	MIC (μg/mL)		MBC (μg/mL)	
	MRSA	VISA	MRSA	VISA
**4d**	1.2–2.4	N/D	19.5–39	>1250
**5d**	312.5–625	N/D	625	>1250
**7c**	625	156	625	1250
**7d**	78.1	156	>1250	1250
**7e**	78.1	156	>1250	1250
Pterostilbene	41–161.5	20	41–161.5	20–40

N/D: Non-detection able.

## Supplementary Materials

The following are available online at https://www.mdpi.com/1422-0067/20/18/4564/s1.
