# Supplementary material for: Design, Synthesis, and Anti-Bacterial Evaluation of Triazolyl-Pterostilbene Derivatives
Source: Int J Mol Sci. 2019 Sep 14;20(18):4564. doi: 10.3390/ijms20184564 (PMC6769857; doi:10.3390/ijms20184564)

NMR spectrum of compound **4-7** series

TKW-5919s

Pulse Sequence: s2pul
UNITYplus-400 "unity400"
Date: Jan 12 2018
Solvent: DMSO
Ambient temperature
Total 32 repetitions

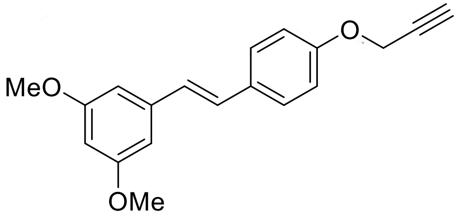

3

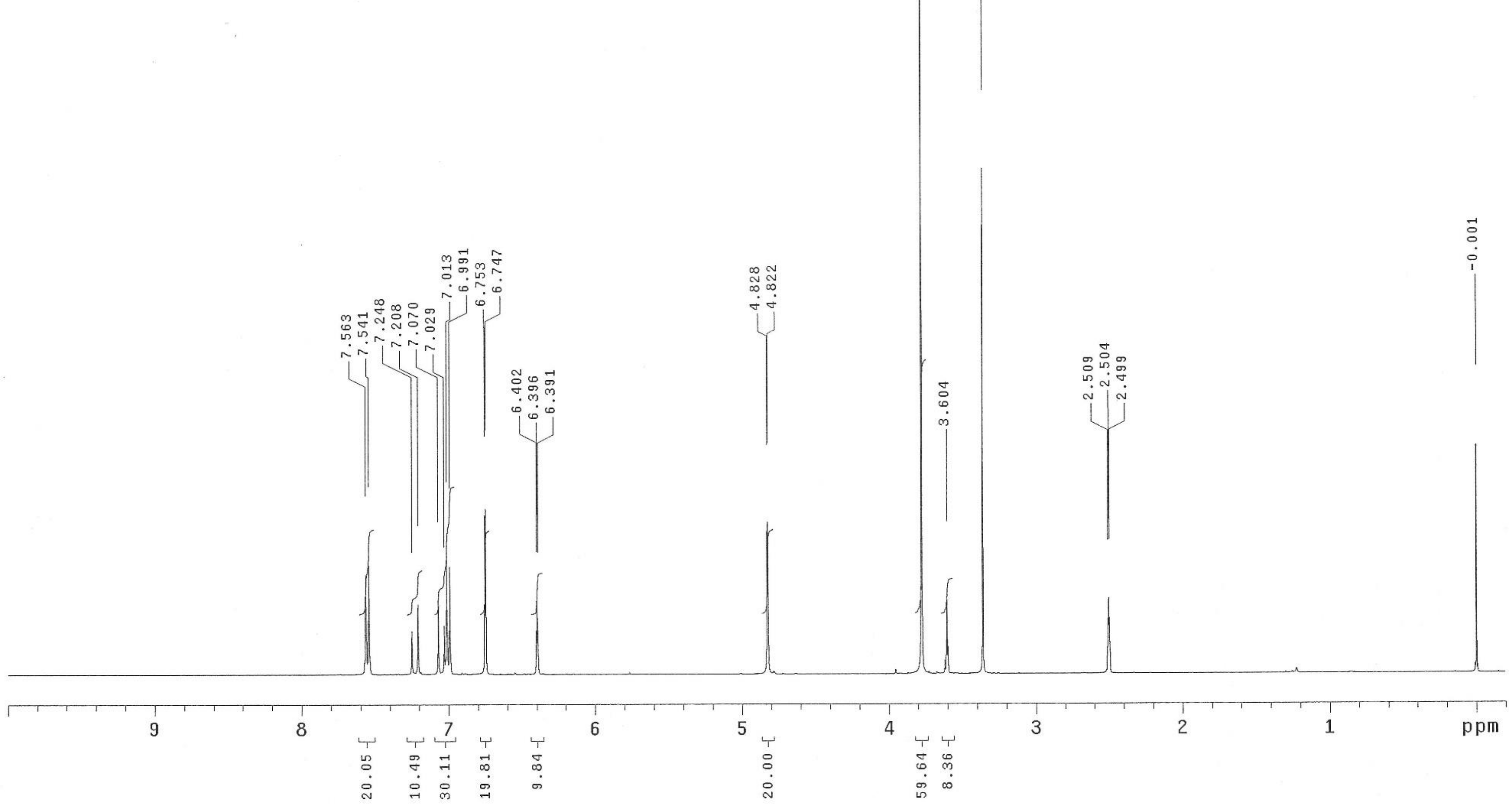

TKW-5919s

Pulse Sequence: s2pul
UNITYplus-400 "unity400"
Date: Jan 12 2018
Solvent: DMSO
Ambient temperature
Total 2288 repetitions

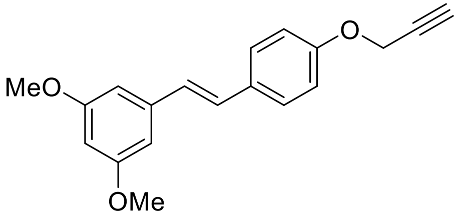

3

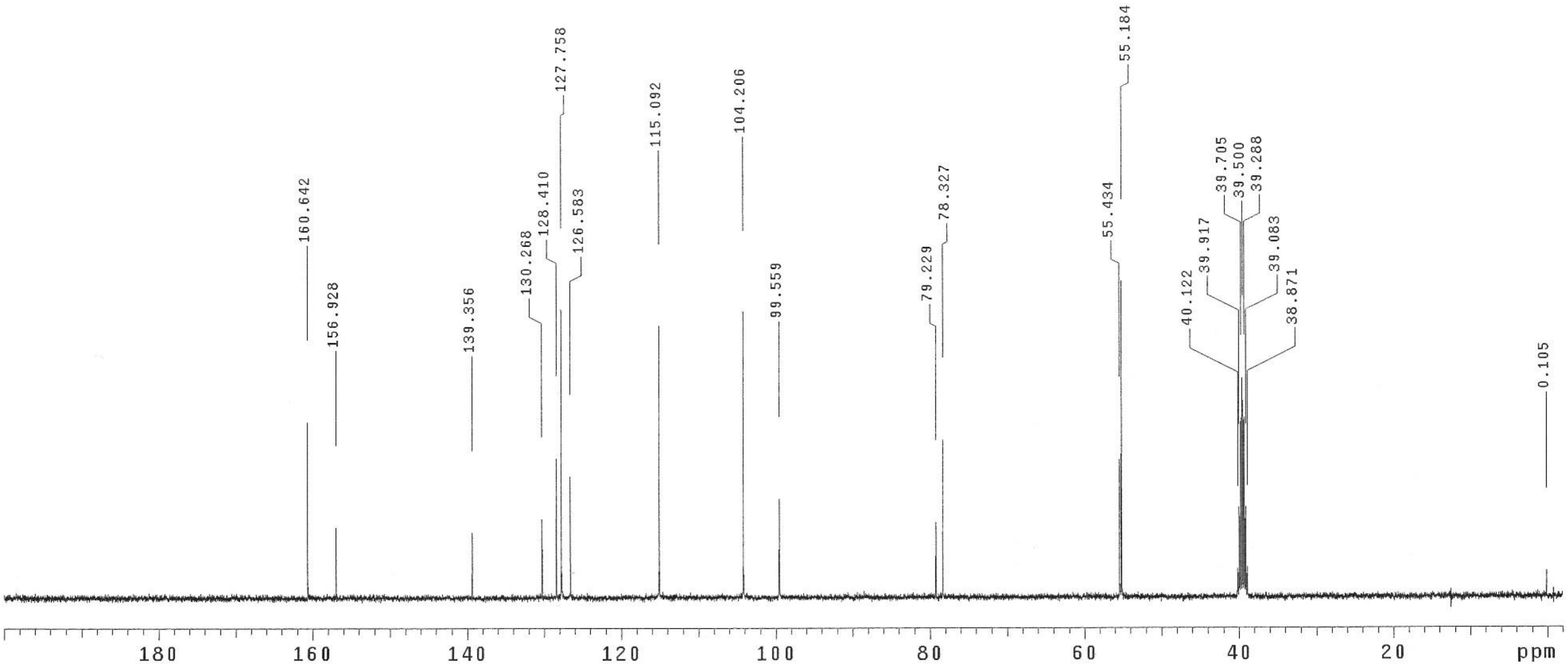

TKW-5920t

Pulse Sequence: s2pul
UNITYplus-400 "unity400"
Date: Jan 16 2018
Solvent: DMSO
Ambient temperature
Total 32 repetitions

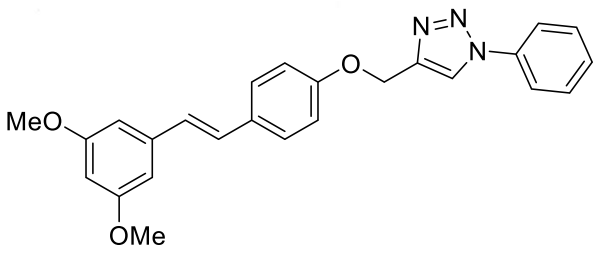

4a

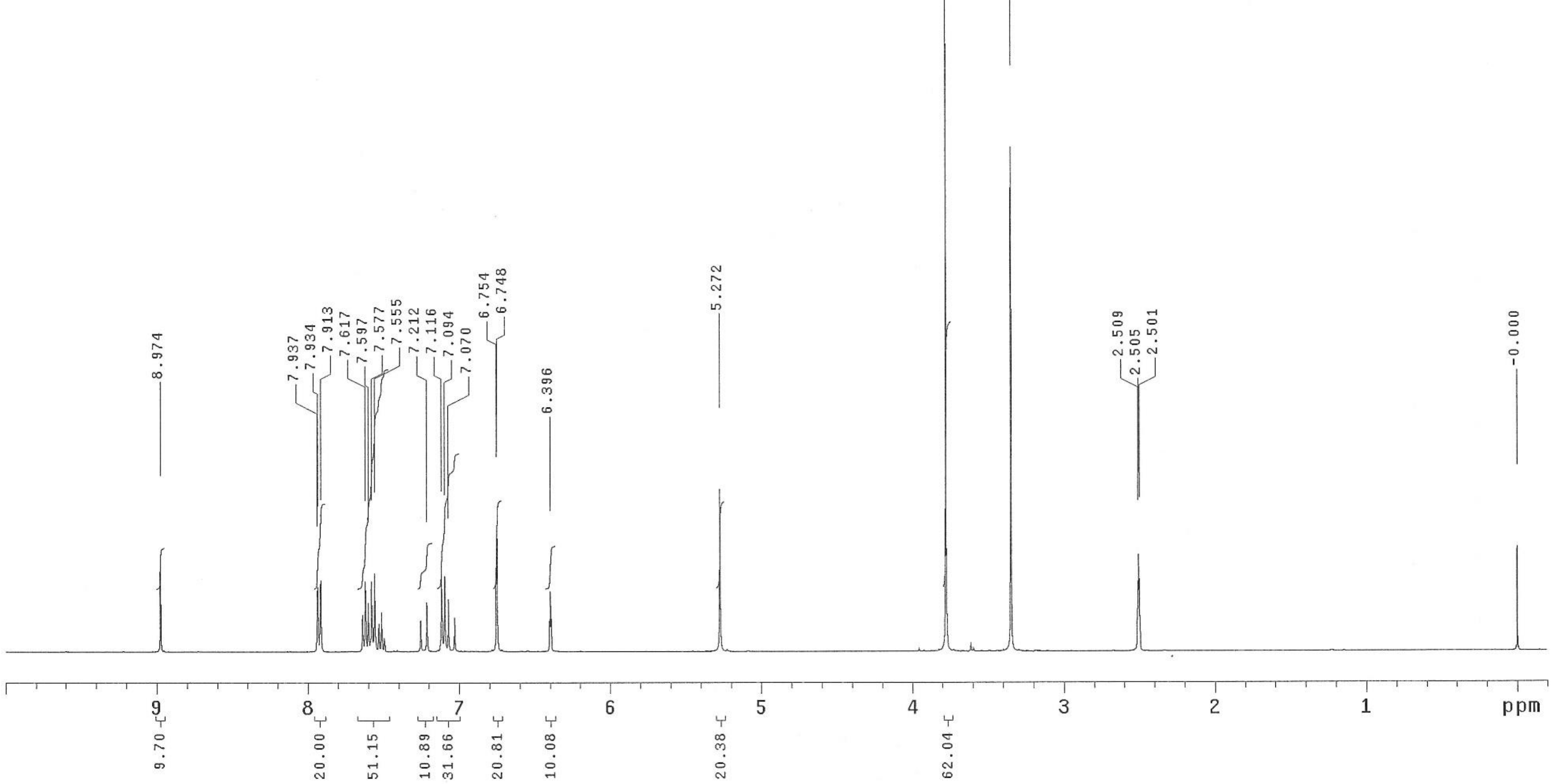

TKW-5920t

Pulse Sequence: s2pul
UNITYplus-400 "unity400"
Date: Jan 16 2018
Solvent: DMSO
Ambient temperature
Total 2080 repetitions

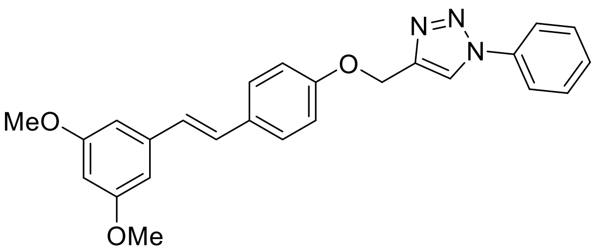

4a

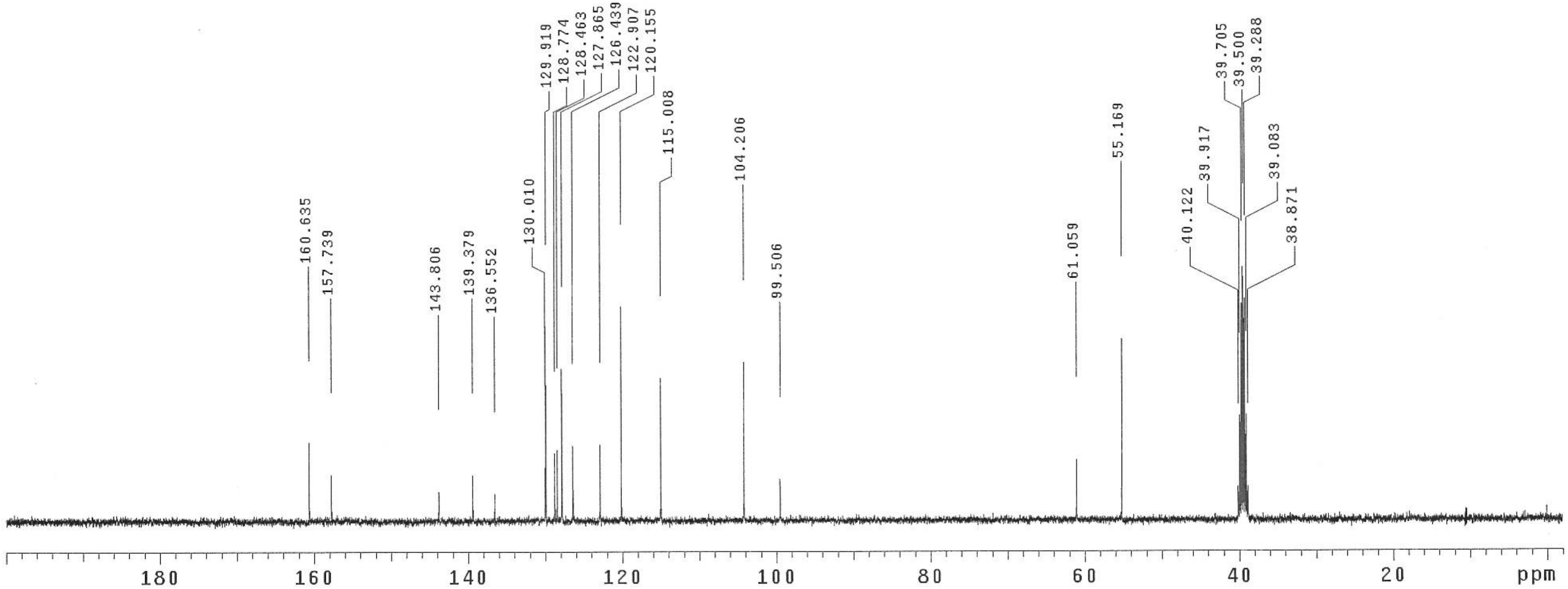

TKW-5921u

Pulse Sequence: s2pul
UNITYplus-400 "unity400"
Date: Jan 16 2018
Solvent: DMSO
Ambient temperature
Total 32 repetitions

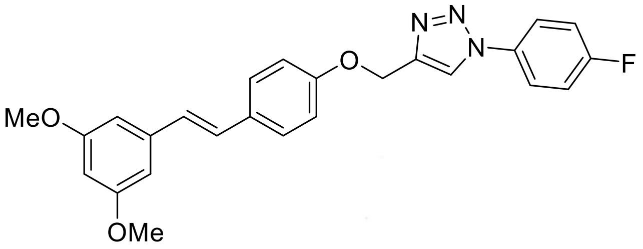

4b

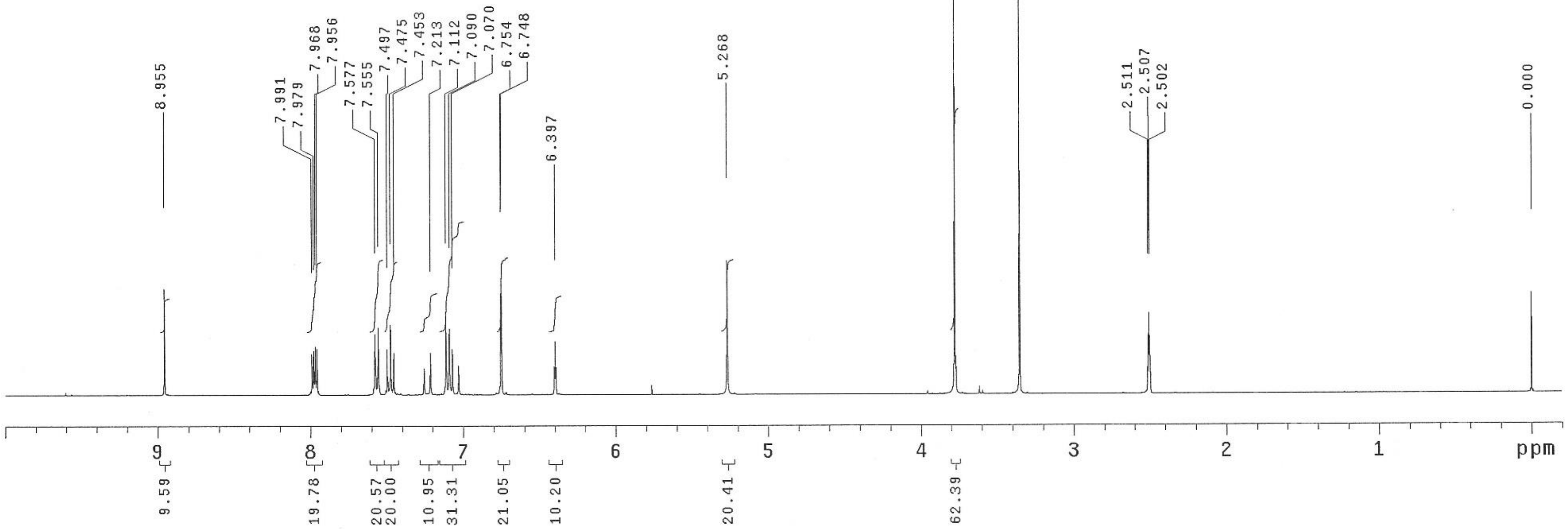

TKW-5921u

Pulse Sequence: s2pul
UNITYplus-400 "unity400"
Date: Jan 16 2018
Solvent: DMSO
Ambient temperature
Total 3472 repetitions

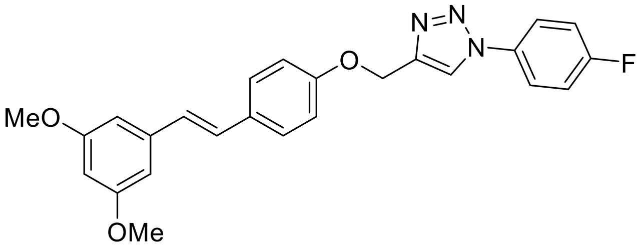

4b

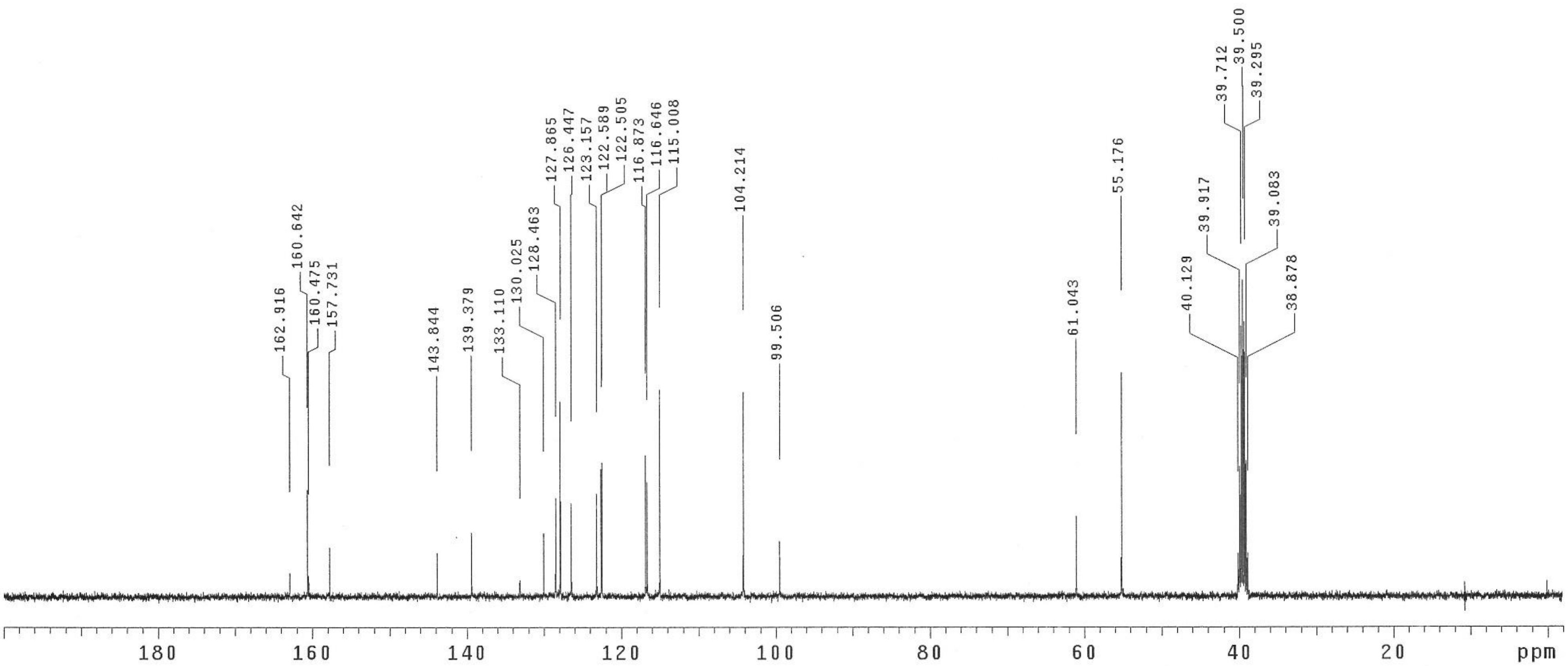

TKW-5922v

Pulse Sequence: s2pul
Mercury-400BB "MerPlus400"
Date: Jan 19 2018
Solvent: dmso
Ambient temperature
Total 32 repetitions

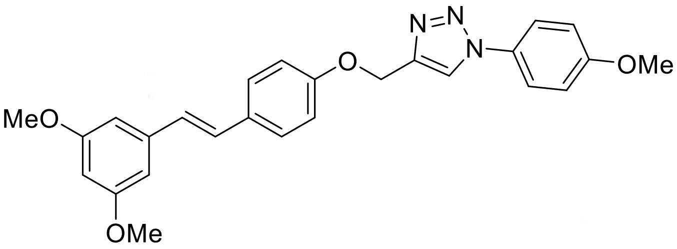

4c

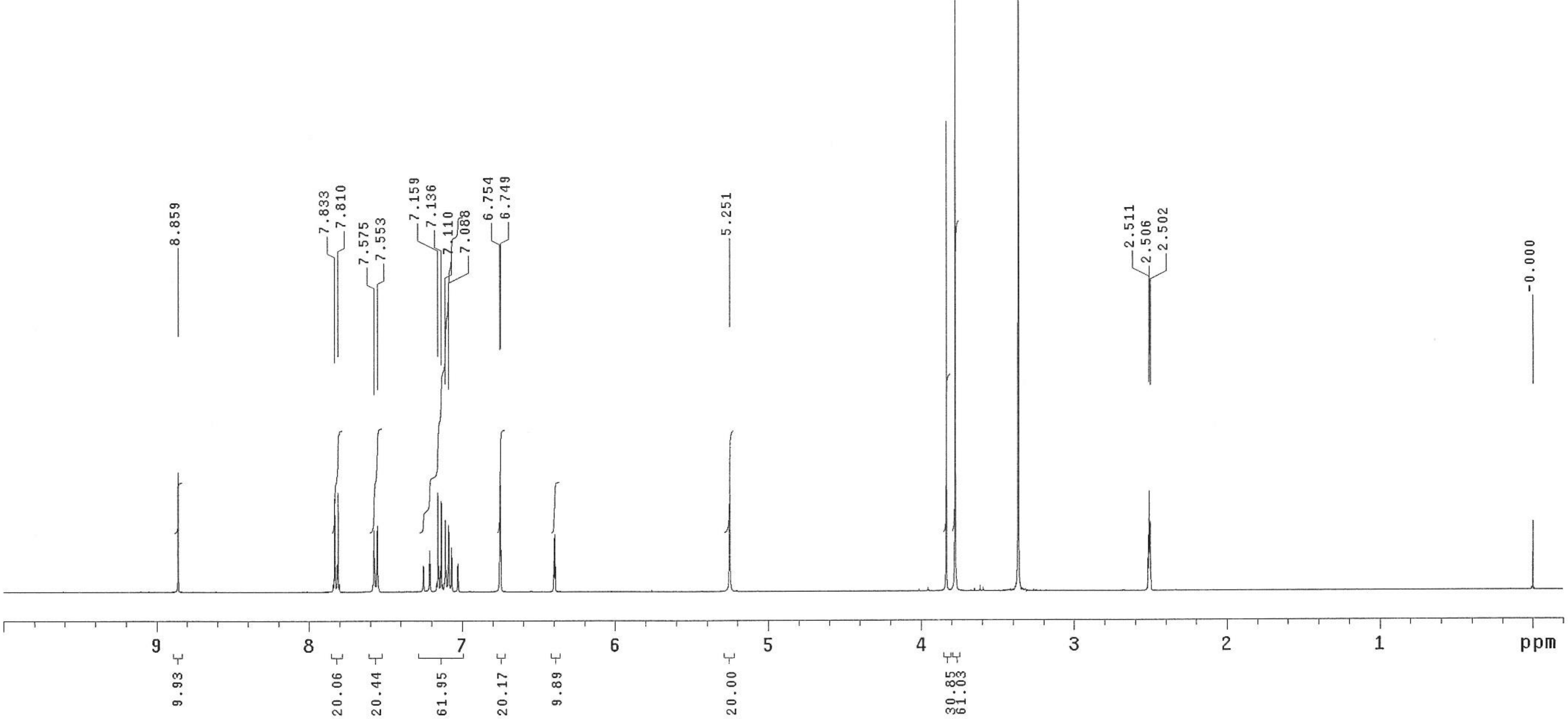

TKW-5922v

Pulse Sequence: s2pul
Mercury-400BB "MerPlus400"
Date: Jan 19 2018
Solvent: dmso
Ambient temperature
Total 5264 repetitions

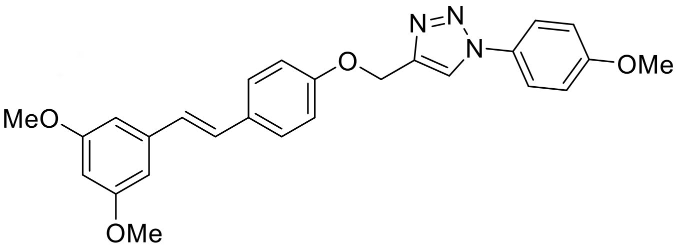

4c

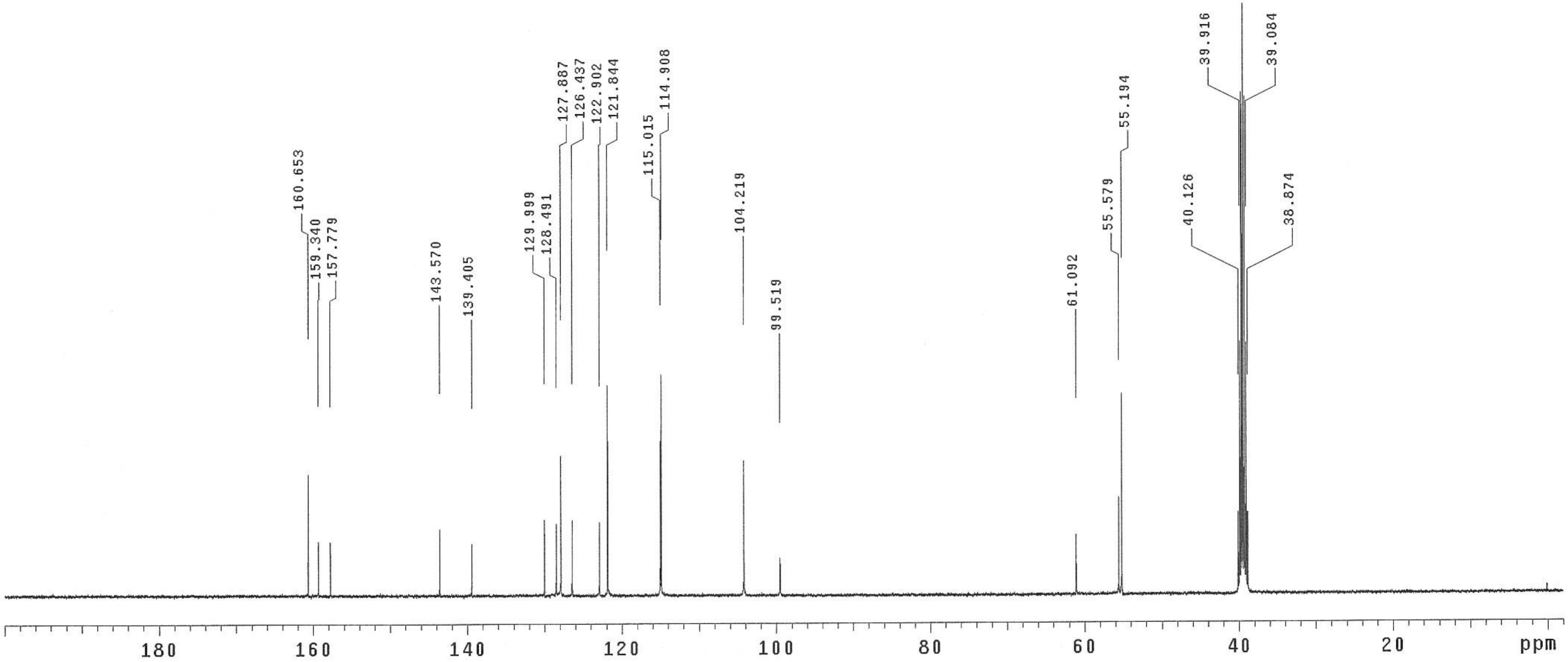

TKW-5923w

Pulse Sequence: s2pul
UNITYplus-400 "unity400"
Date: Jan 26 2018
Solvent: DMSO
Ambient temperature
Total 32 repetitions

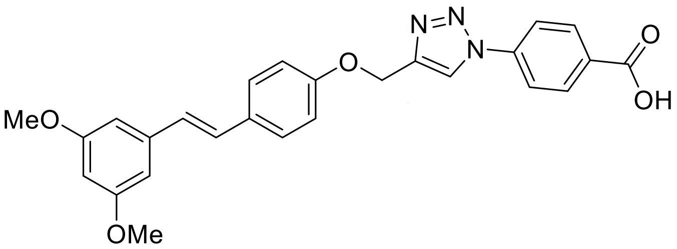

4d

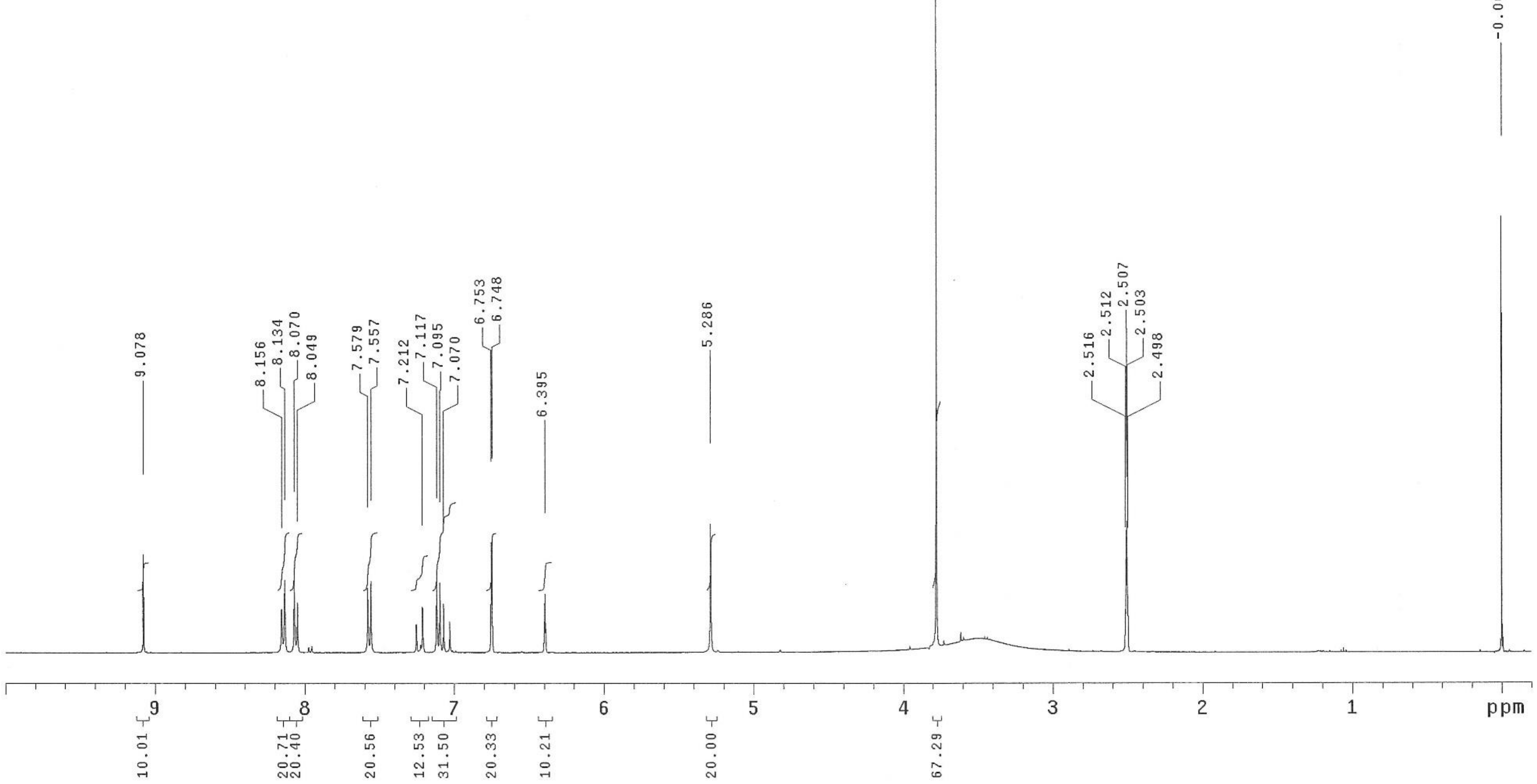

TKW-5923w

Pulse Sequence: s2pul

UNITYplus-400 "unity400"
Date: Jan 26 2018
Solvent: DMSO
Ambient temperature
Total 7152 repetitions

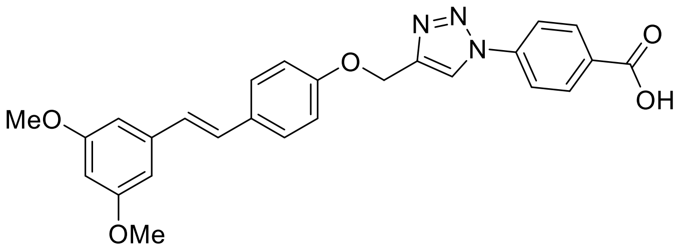

4d

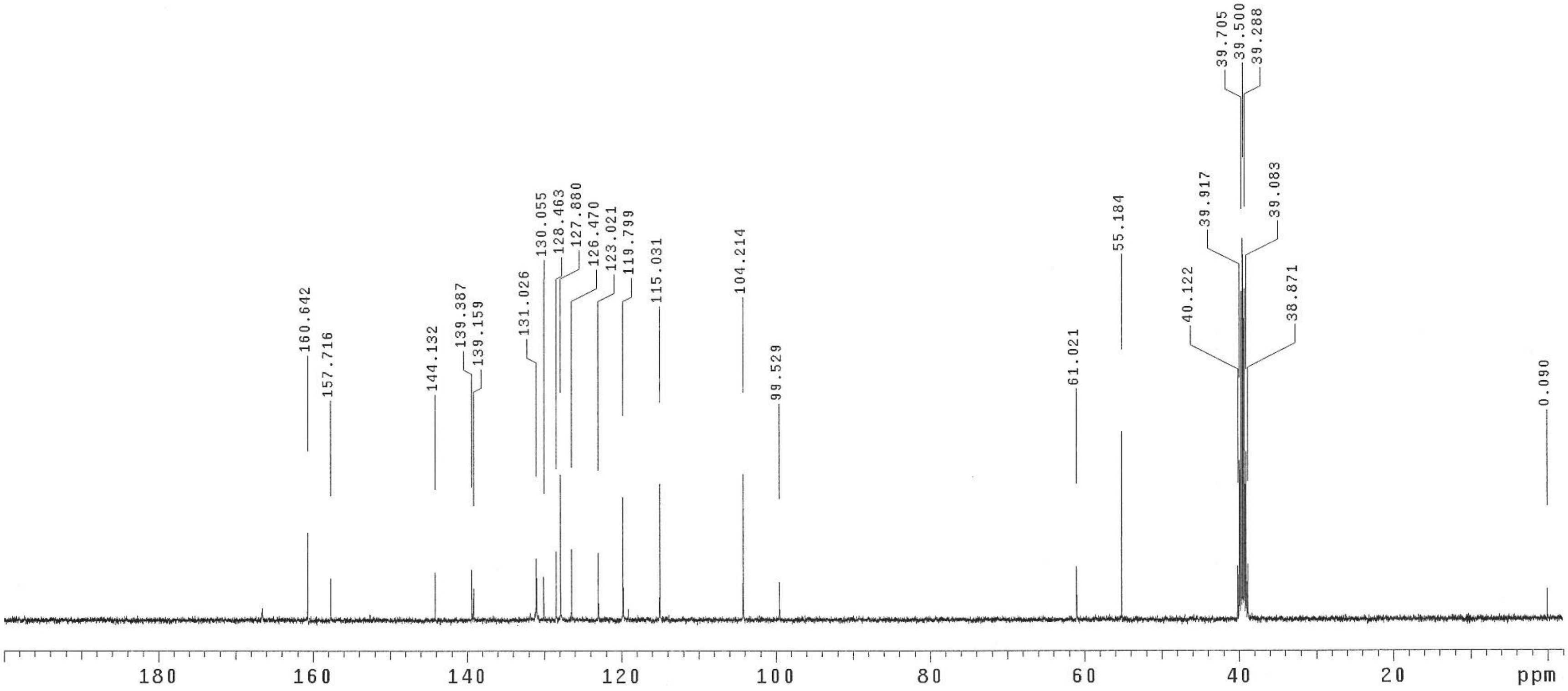

TKW-6107

Pulse Sequence: s2pul
UNITYplus-400 "unity400"
Date: May 31 2018
Solvent: DMSO
Ambient temperature
Total 32 repetitions

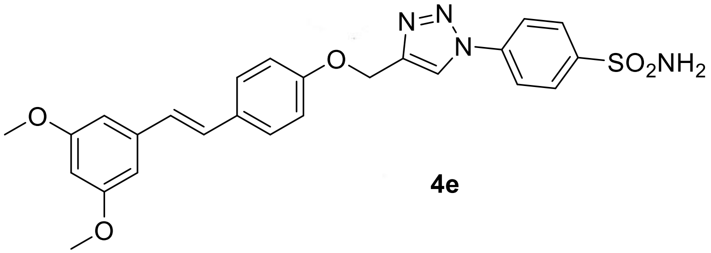

4e

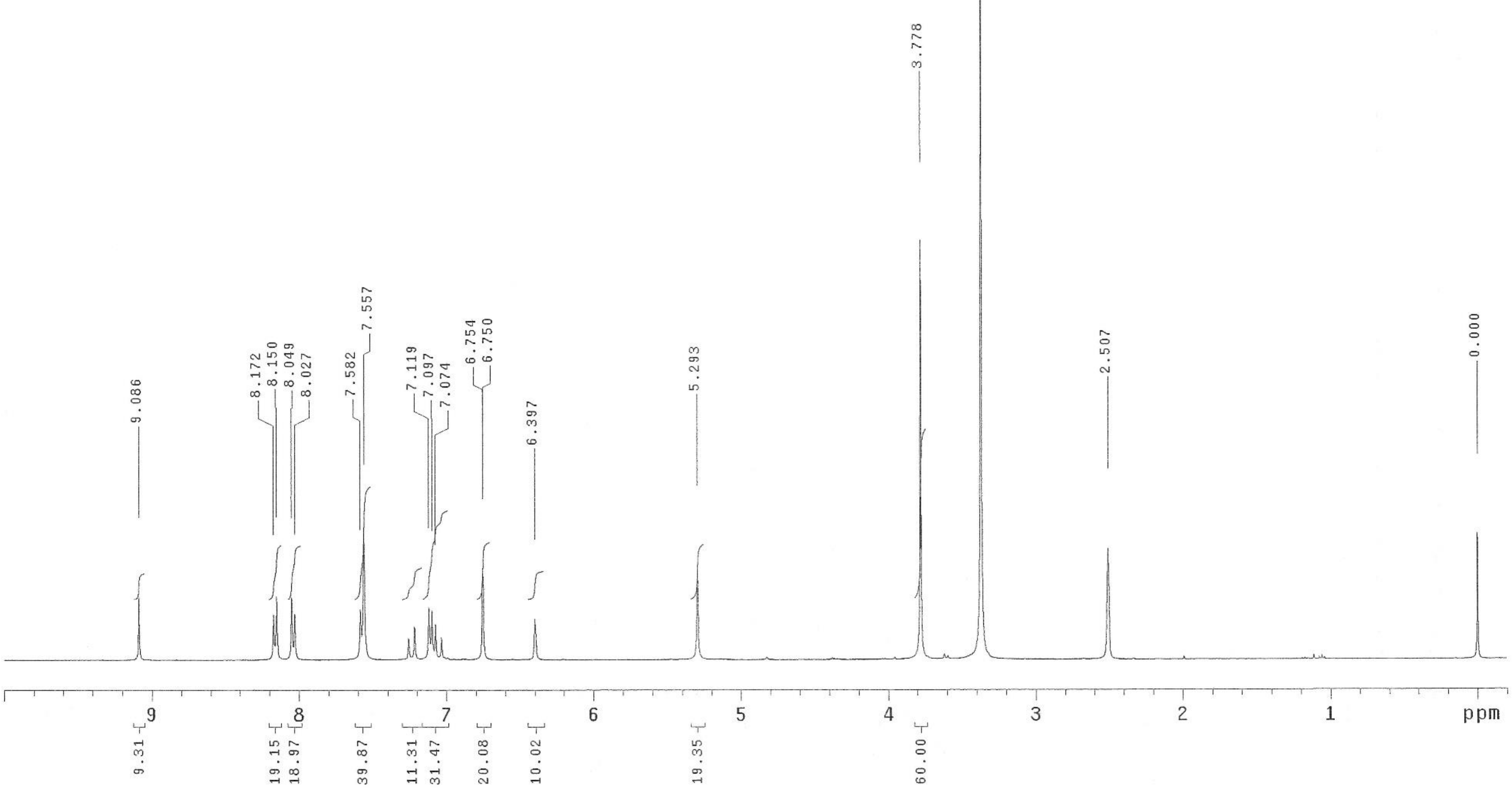

TKW-6107

Pulse Sequence: s2pul

UNITYplus-400 "unity400"
Date: May 31 2018
Solvent: DMSO
Ambient temperature
Total 1616 repetitions

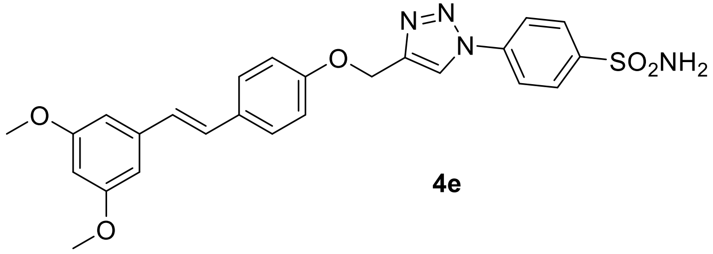

4e

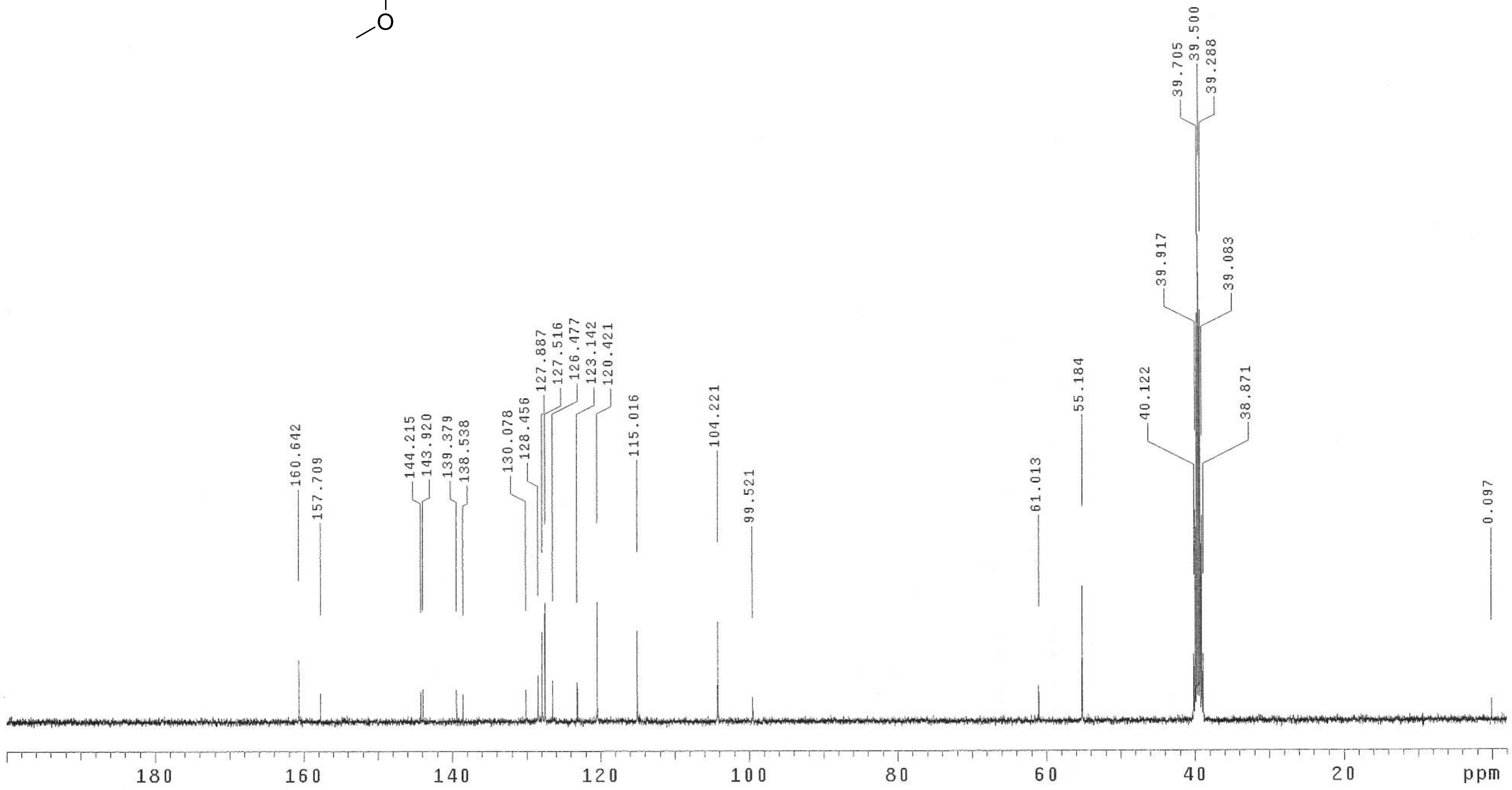

TKW-61121

Pulse Sequence: s2pul
Mercury-400BB "MerPlus400"
Date: Jun 29 2018
Solvent: dmso
Ambient temperature
Total 32 repetitions

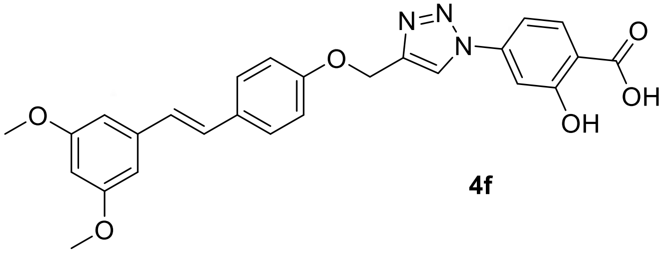

4f

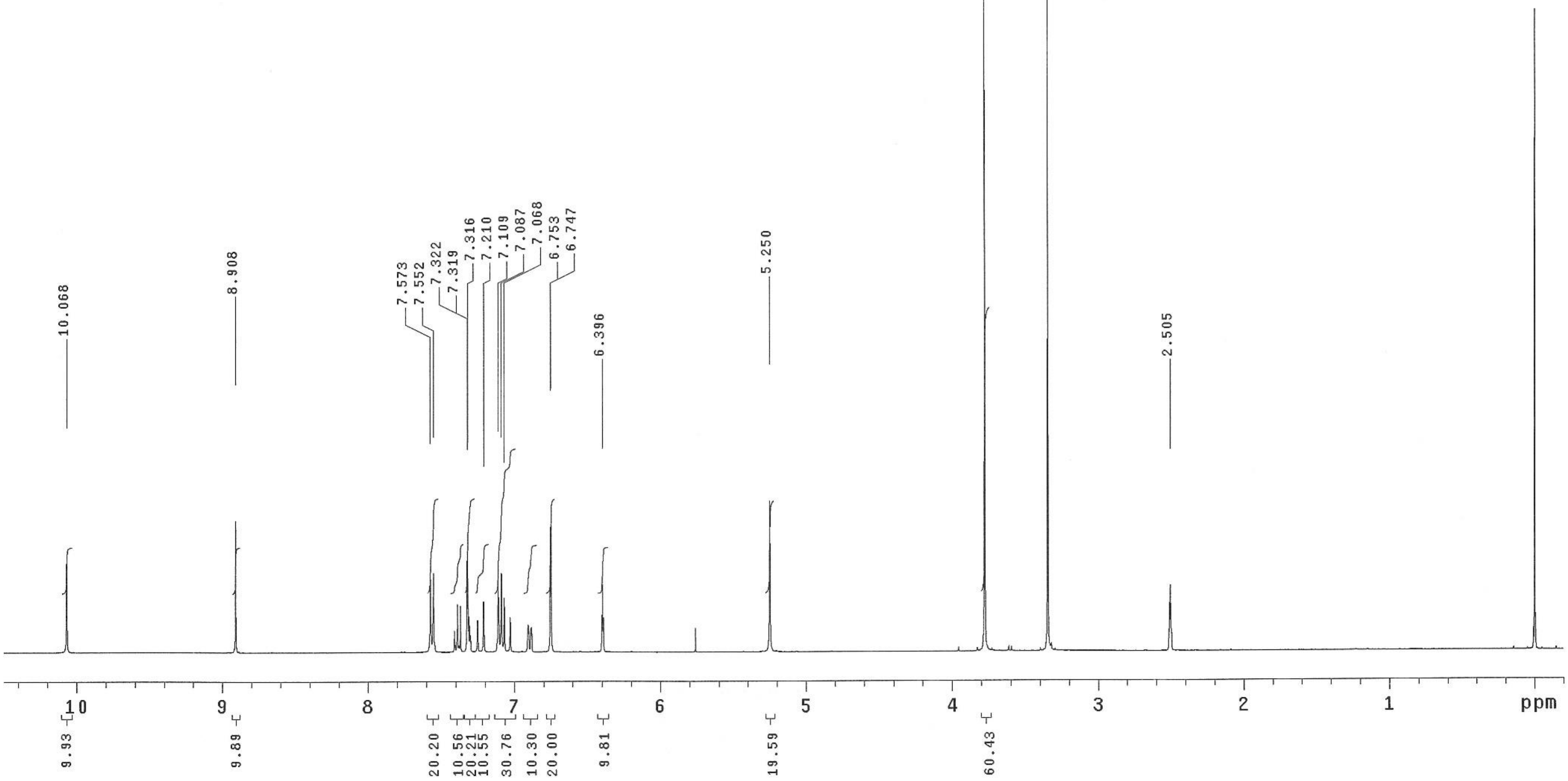

TKW-61121

Pulse Sequence: s2pul
Mercury-400BB "MerPlus400"
Date: Jun 29 2018
Solvent: dmso
Ambient temperature
Total 1040 repetitions

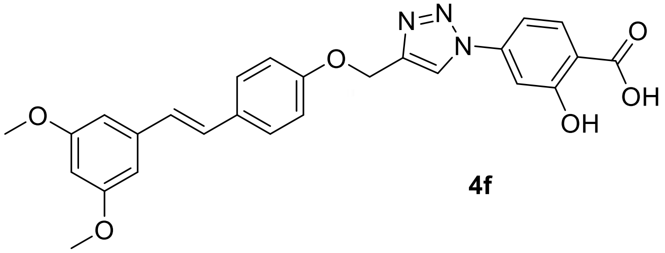

4f

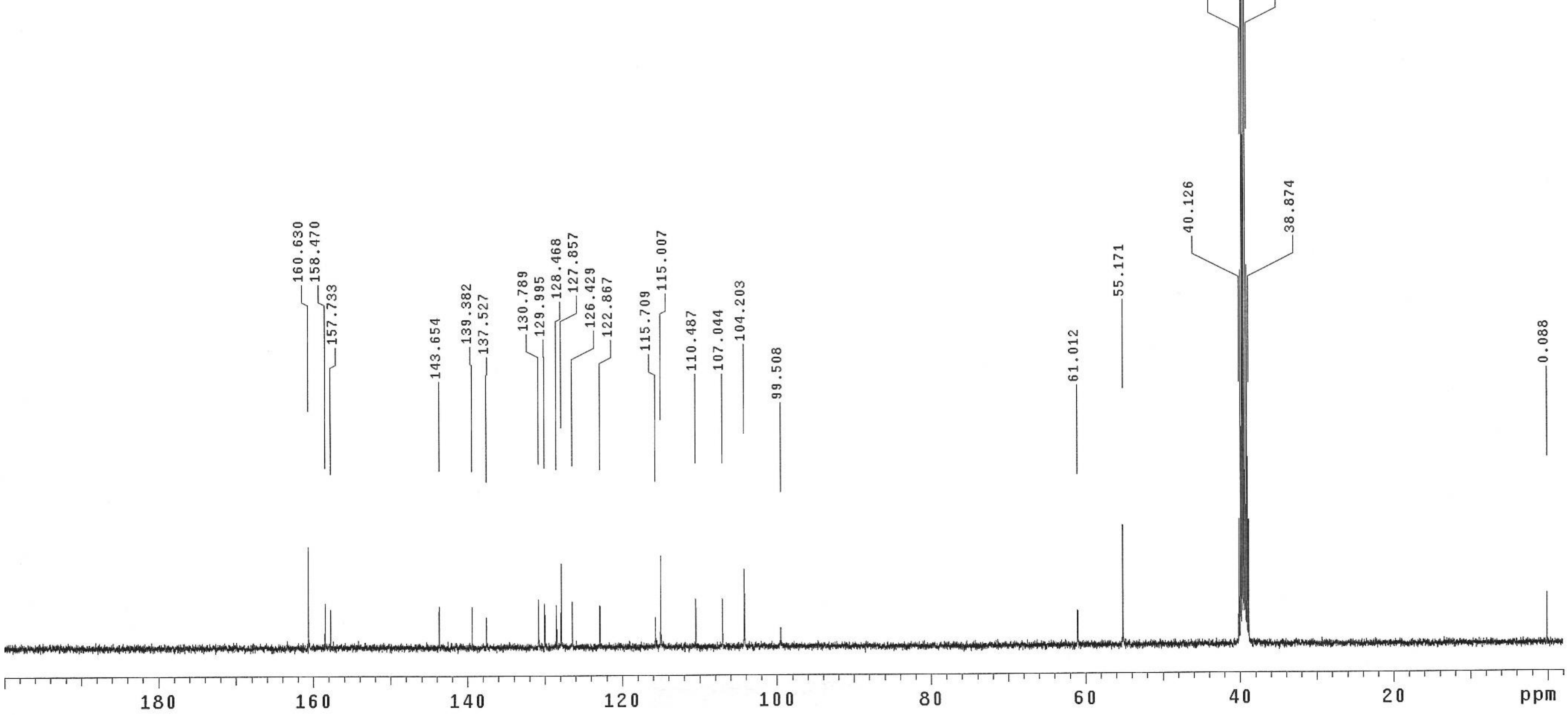

TKW-6111K

Pulse Sequence: s2pul
Mercury-400BB "MerPlus400"
Date: Jun 29 2018
Solvent: dmso
Ambient temperature
Total 32 repetitions

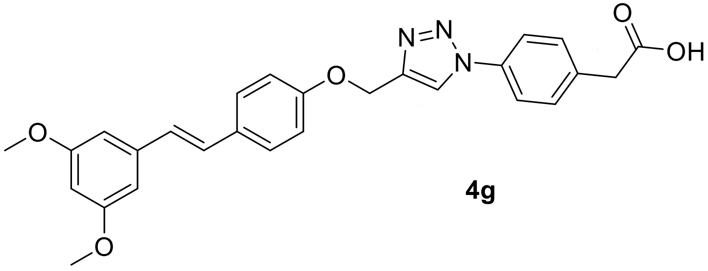

4g

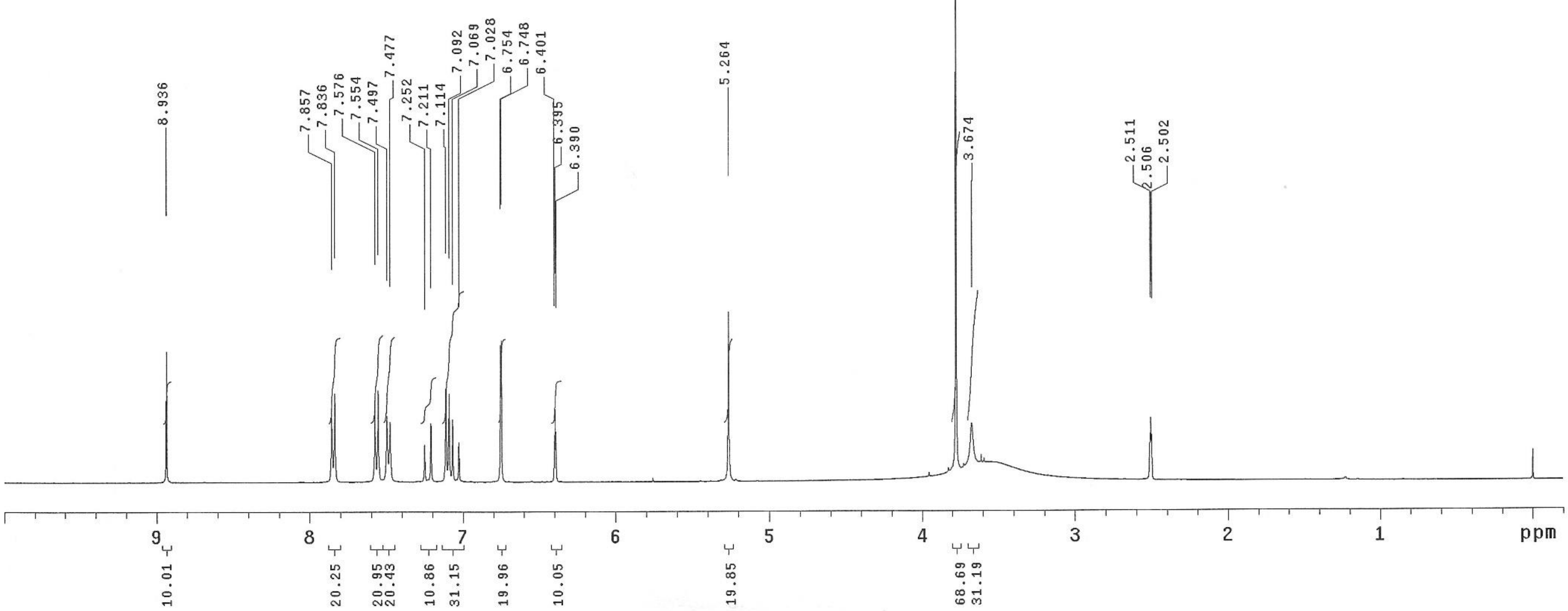

TKW-6111K

Pulse Sequence: s2pul
Mercury-400BB "MerPlus400"
Date: Jun 29 2018
Solvent: dmso
Ambient temperature
Total 1600 repetitions

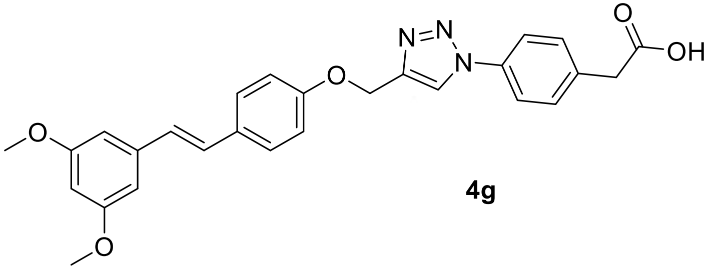

4g

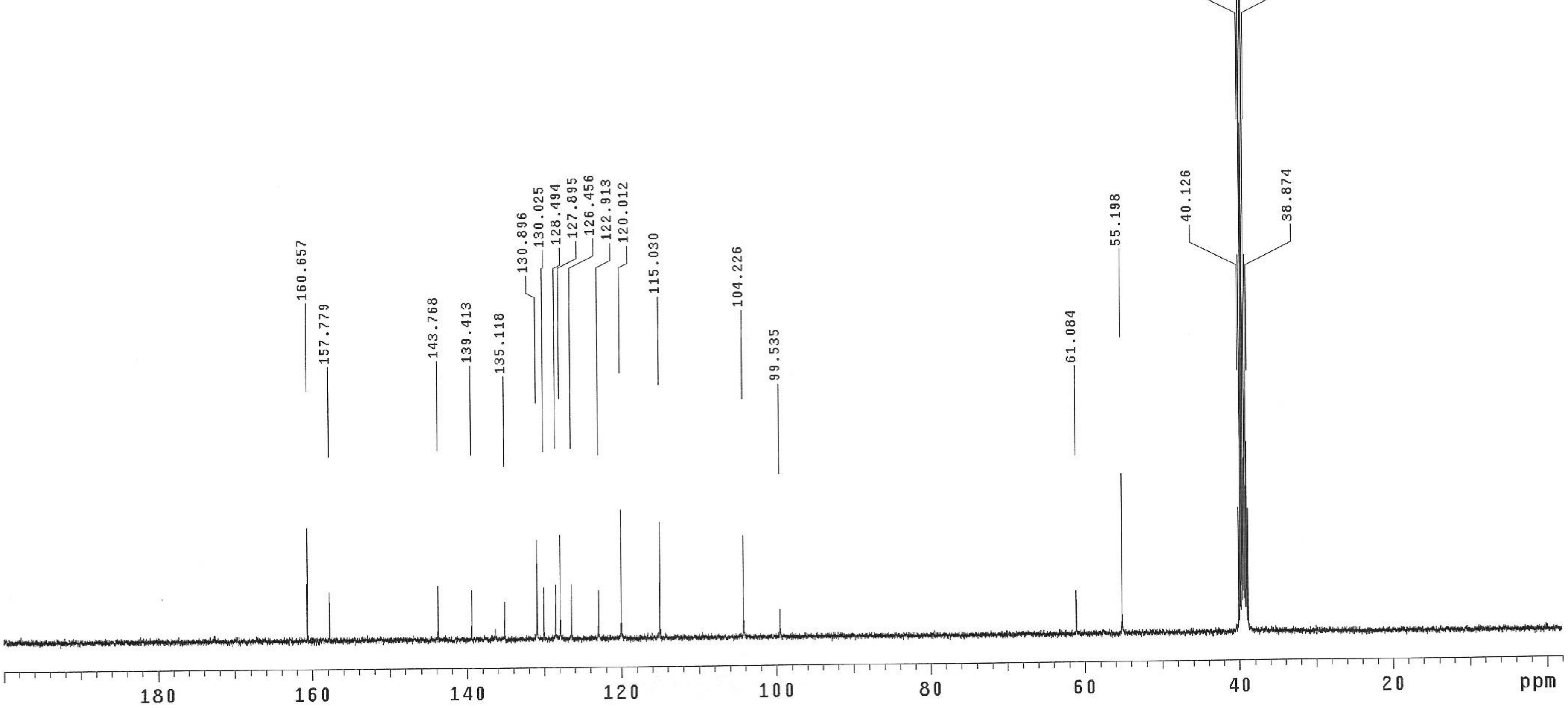

TKW-5925y

Pulse Sequence: s2pul
Mercury-400BB "MerPlus400"
Date: Feb 6 2018
Solvent: dmso
Ambient temperature
Total 28 repetitions

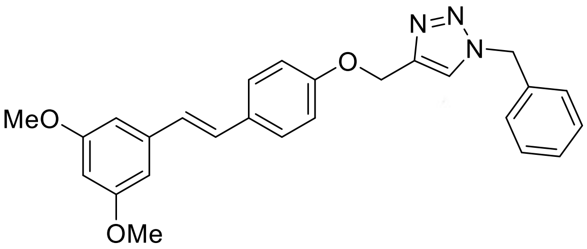

5a

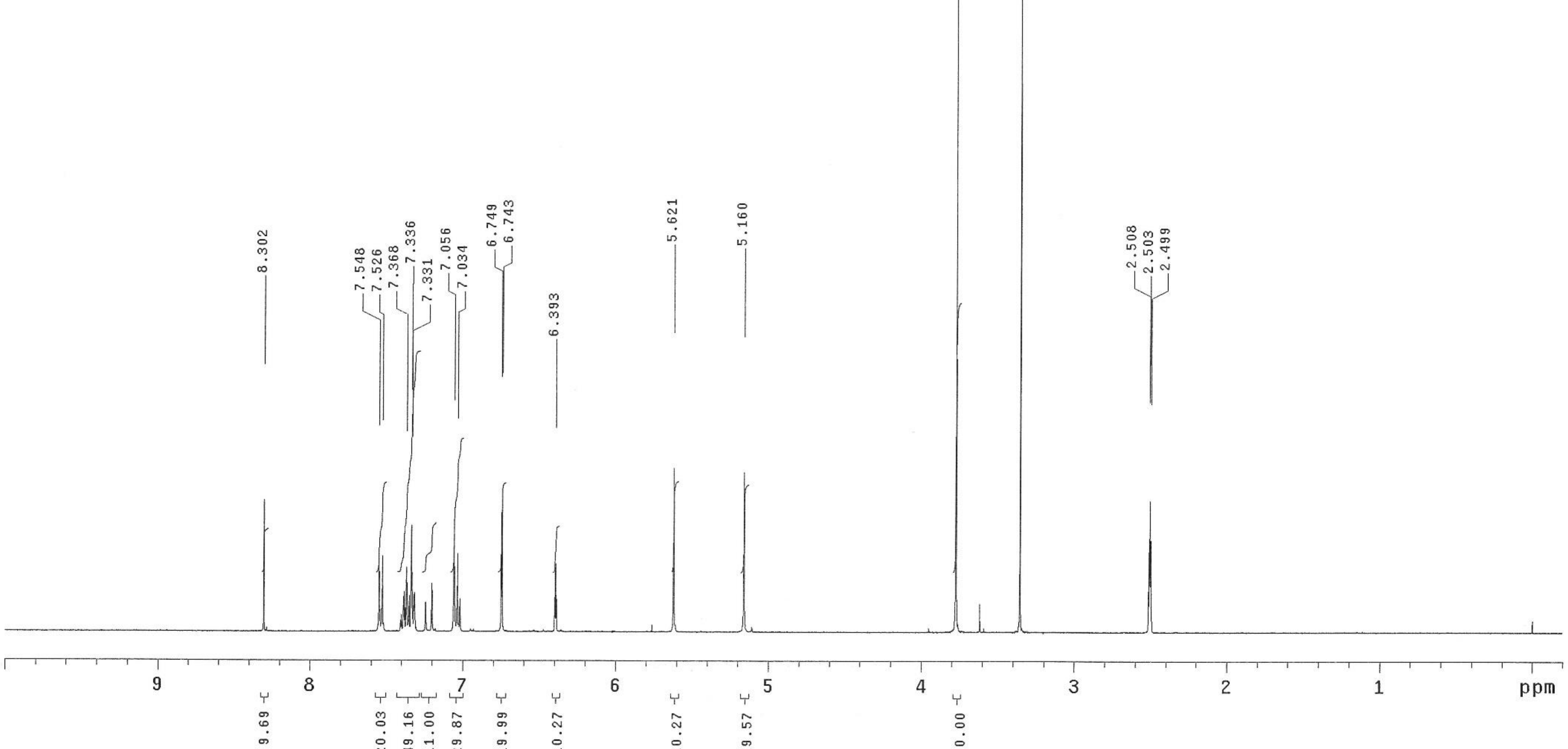

TKW-5925y

Pulse Sequence: s2pul
Mercury-400BB "MerPlus400"
Date: Feb 6 2018
Solvent: dmso
Ambient temperature
Total 3968 repetitions

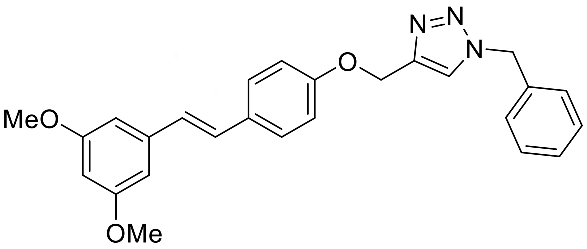

5a

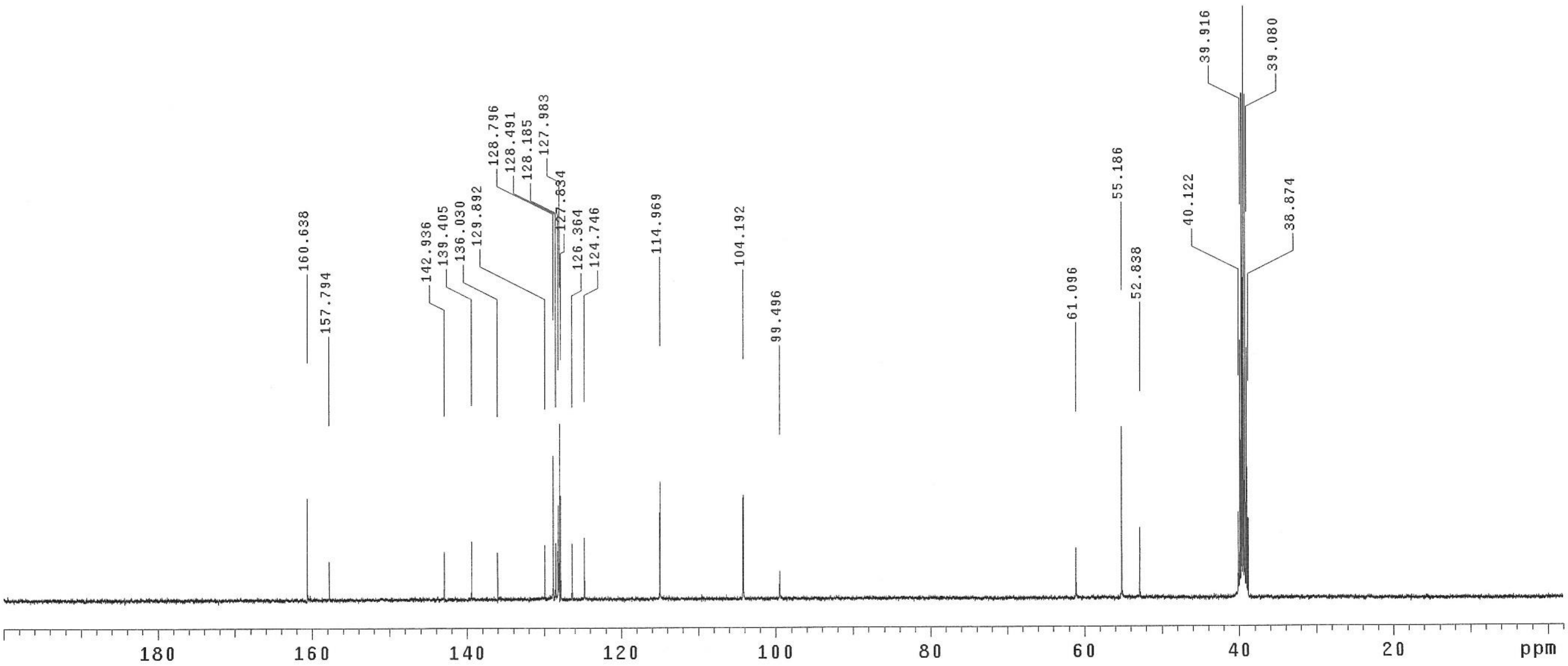

TKW-5926z

Pulse Sequence: s2pul
Mercury-400BB "MerPlus400"
Date: Feb 6 2018
Solvent: dmso
Ambient temperature
Total 32 repetitions

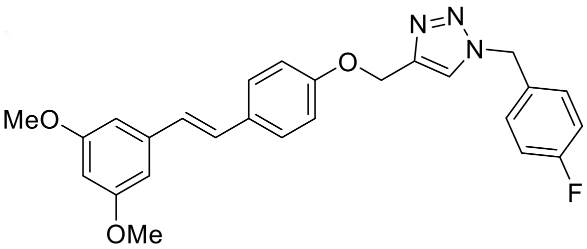

5b

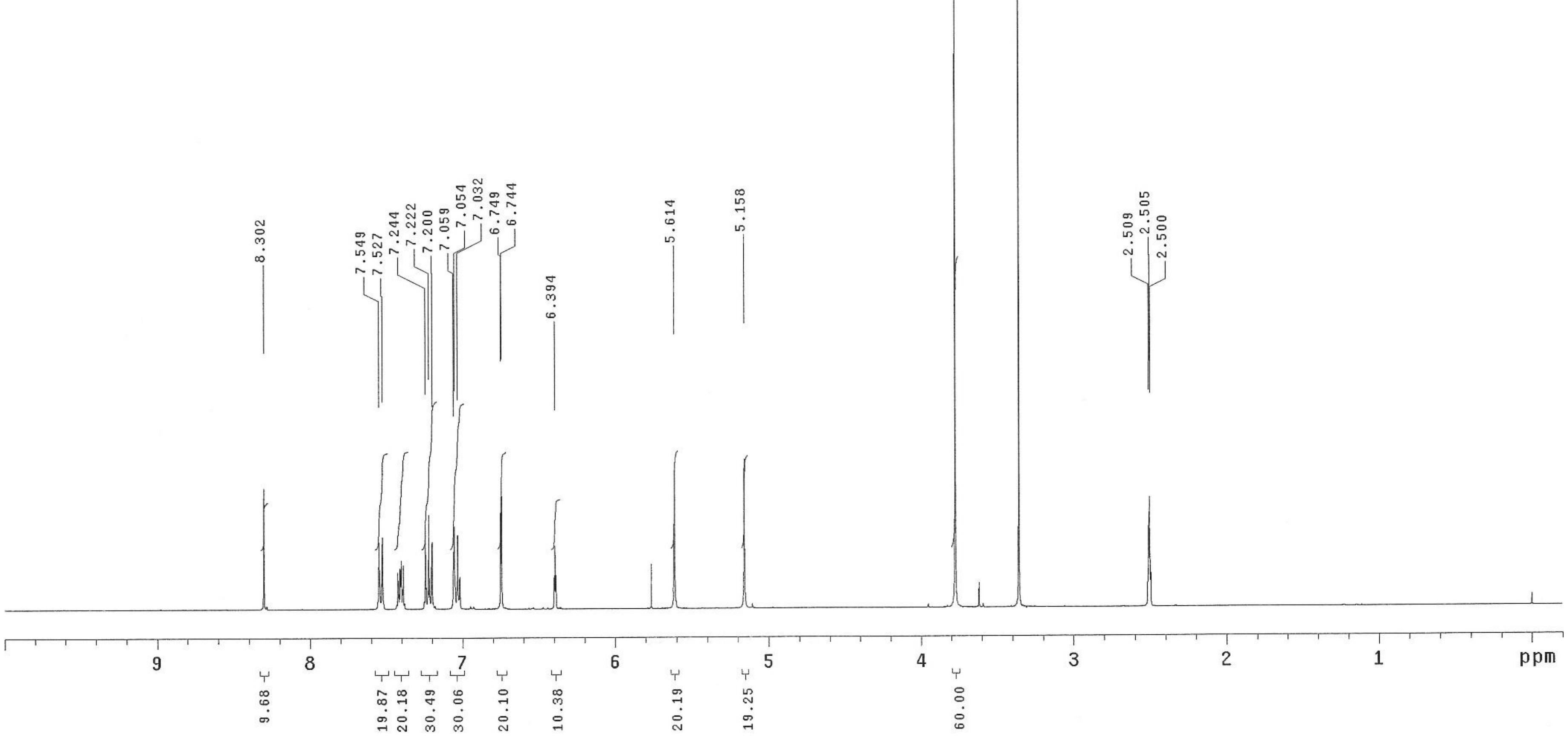

TKW-5926z

Pulse Sequence: s2pul
Mercury-400BB "MerPlus400"
Date: Feb 6 2018
Solvent: dmso
Ambient temperature
Total 1824 repetitions

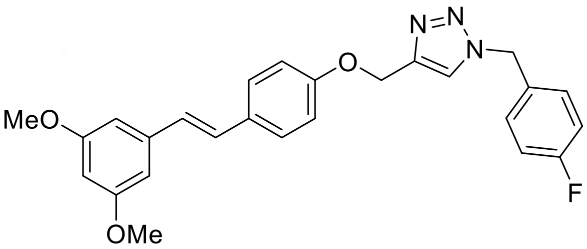

5b

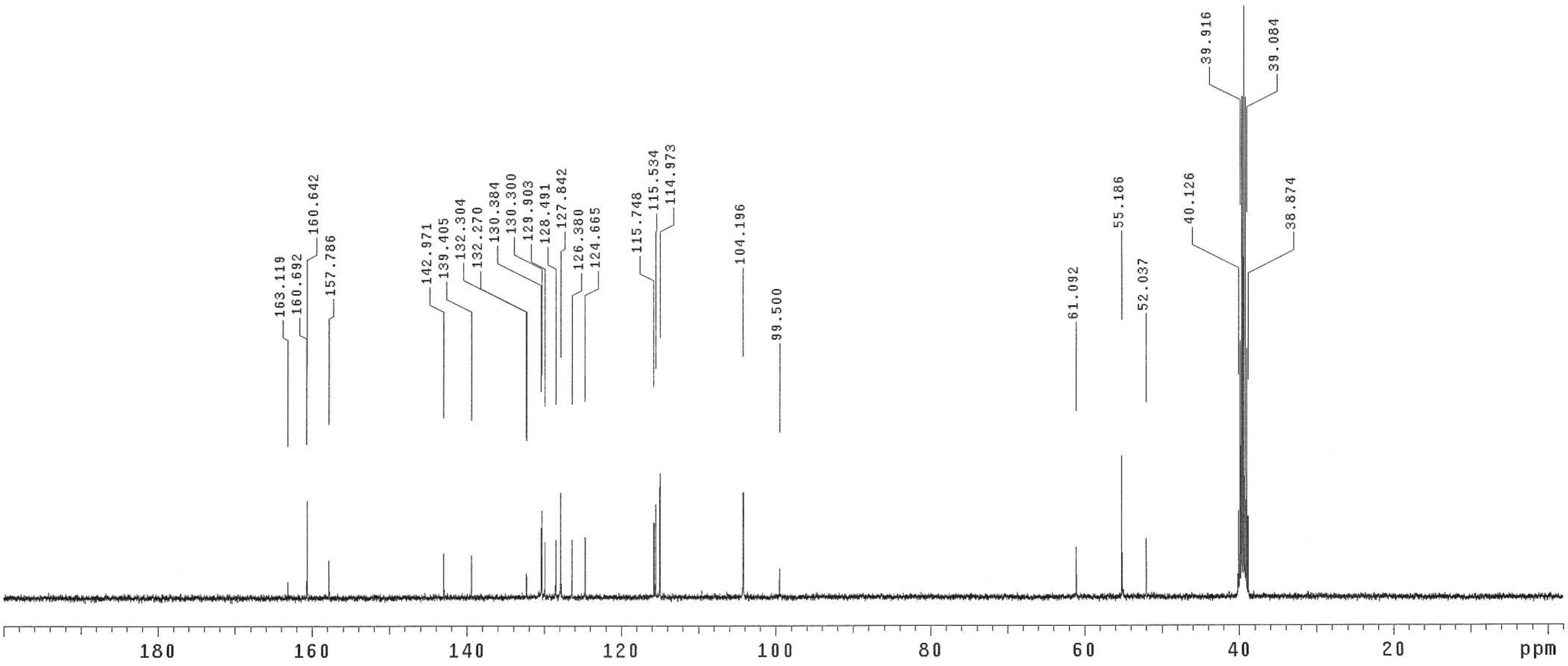

TKW-5927a

Pulse Sequence: s2pul
Mercury-400BB "MerPlus400"
Date: Mar 5 2018
Solvent: dmso
Ambient temperature
Total 32 repetitions

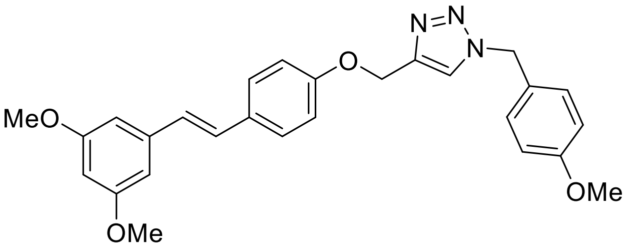

5c

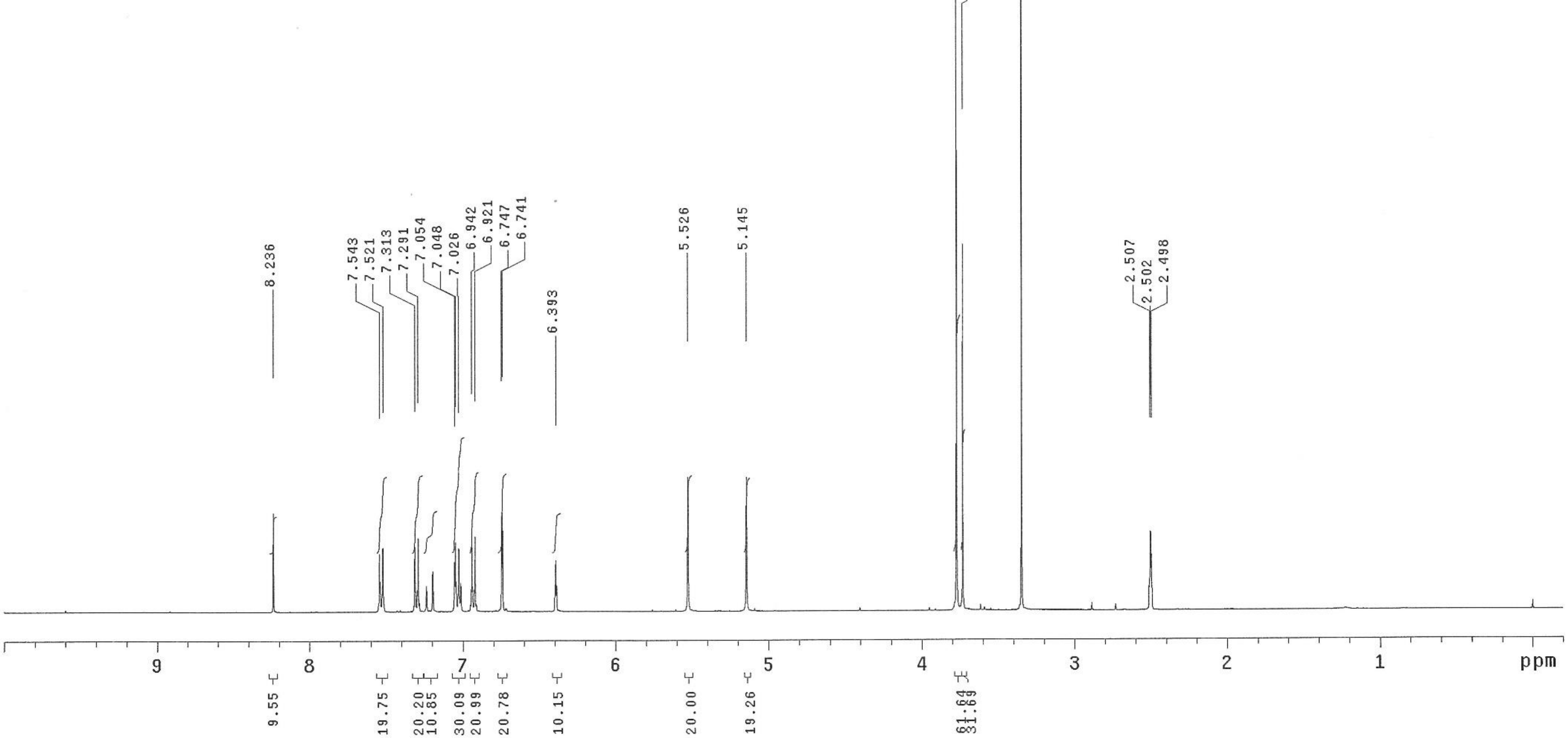

TKW-5927a

Pulse Sequence: s2pul
Mercury-400BB "MerPlus400"
Date: Mar 5 2018
Solvent: dmso
Ambient temperature
Total 1840 repetitions

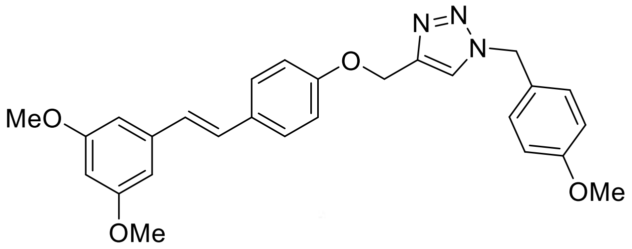

5c

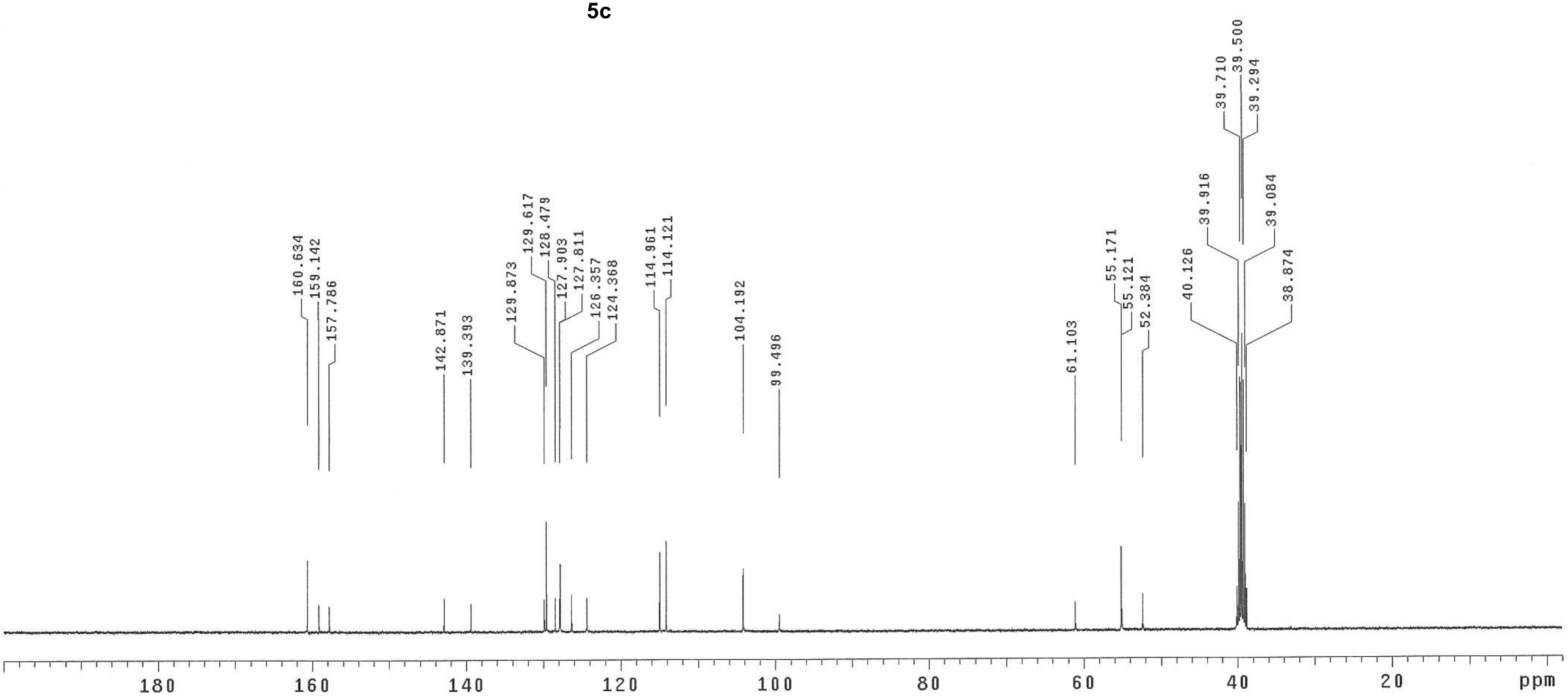

TKW-5928b

Pulse Sequence: s2pul
Mercury-400BB "MerPlus400"
Date: Feb 6 2018
Solvent: dmso
Ambient temperature
Total 32 repetitions

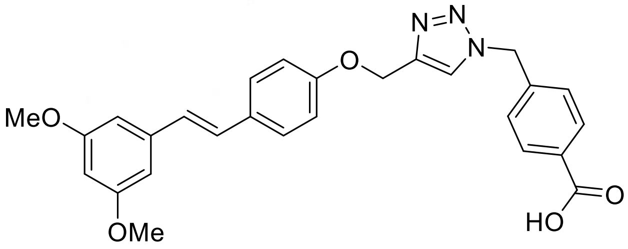

5d

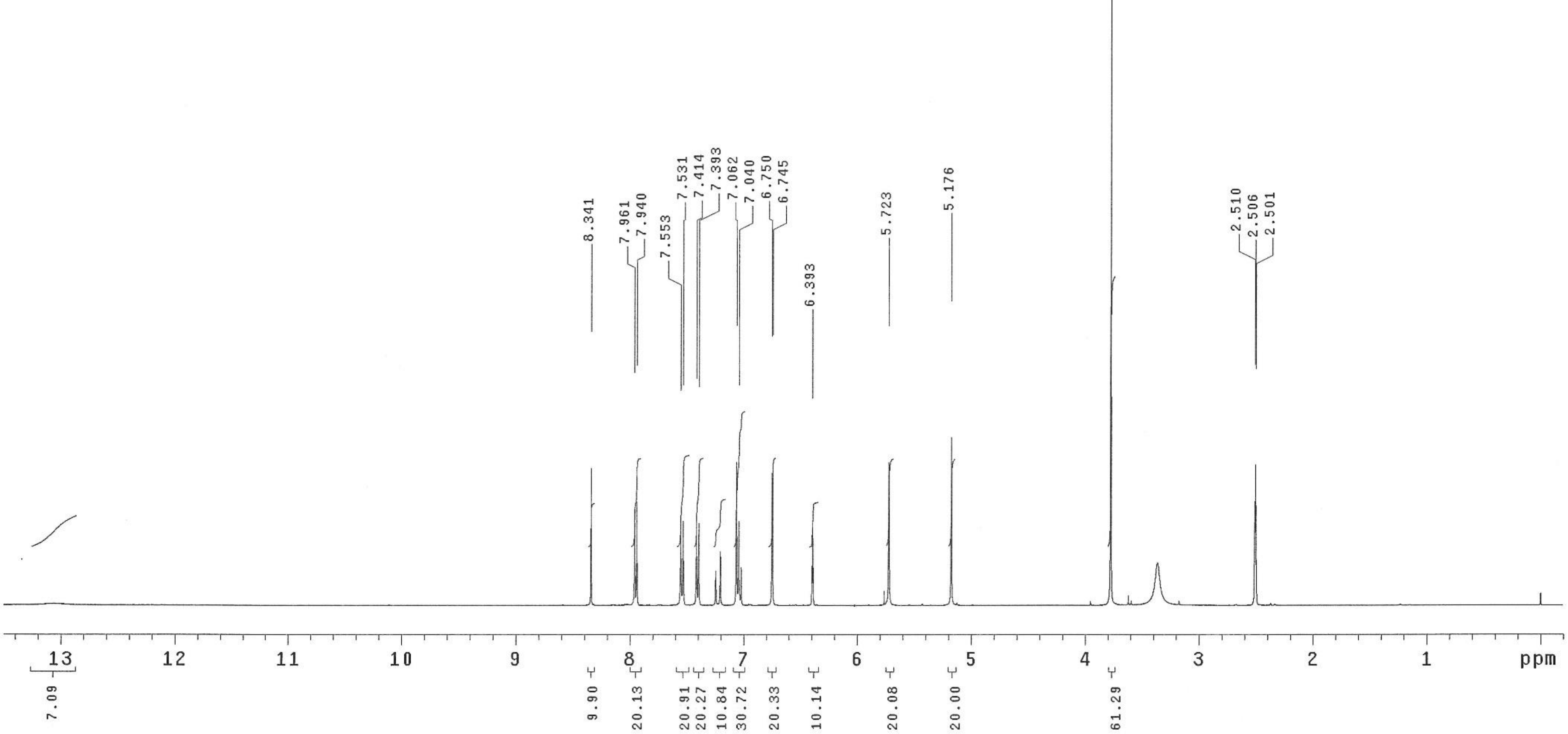

TKW-5928b

Pulse Sequence: s2pul
Mercury-400BB "MerPlus400"
Date: Feb 6 2018
Solvent: dmso
Ambient temperature
Total 64000 repetitions

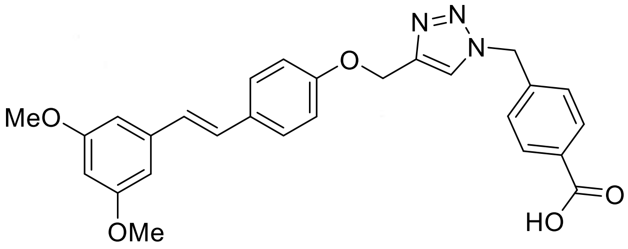

5d

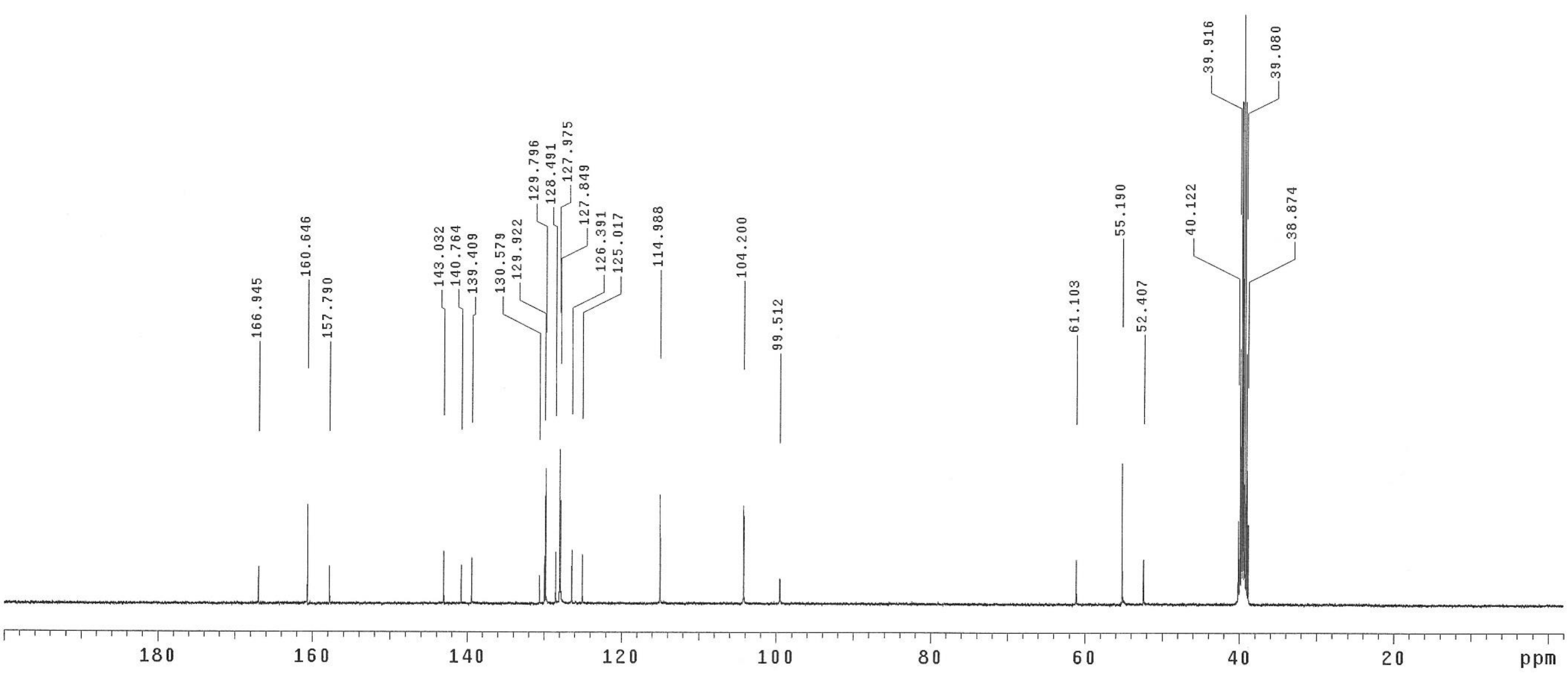

TKW-5929c

Pulse Sequence: s2pul
Mercury-400BB "MerPlus400"
Date: Feb 6 2018
Solvent: dmso
Ambient temperature
Total 32 repetitions

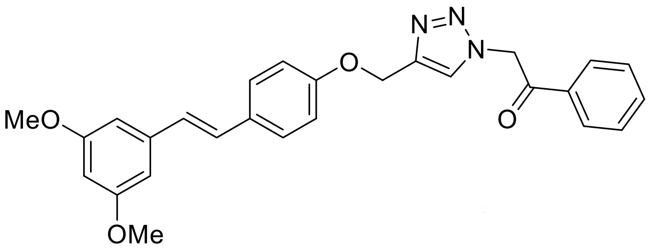

6a

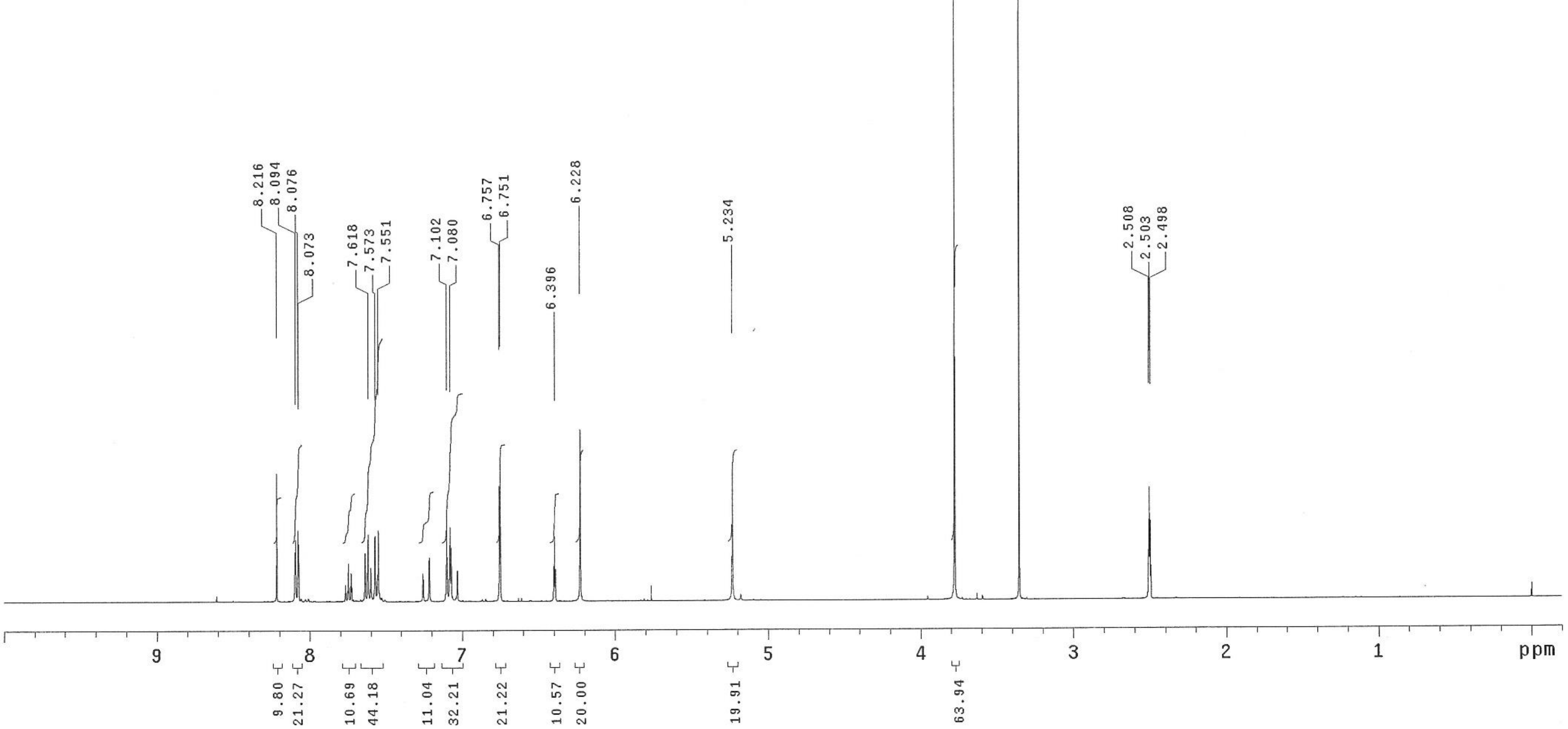

TKW-5929c

Pulse Sequence: s2pul
Mercury-400BB "MerPlus400"
Date: Feb 6 2018
Solvent: dmso
Ambient temperature
Total 1280 repetitions

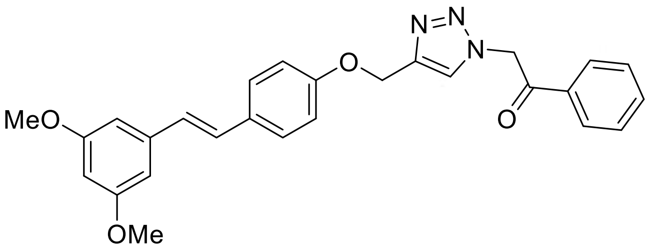

6a

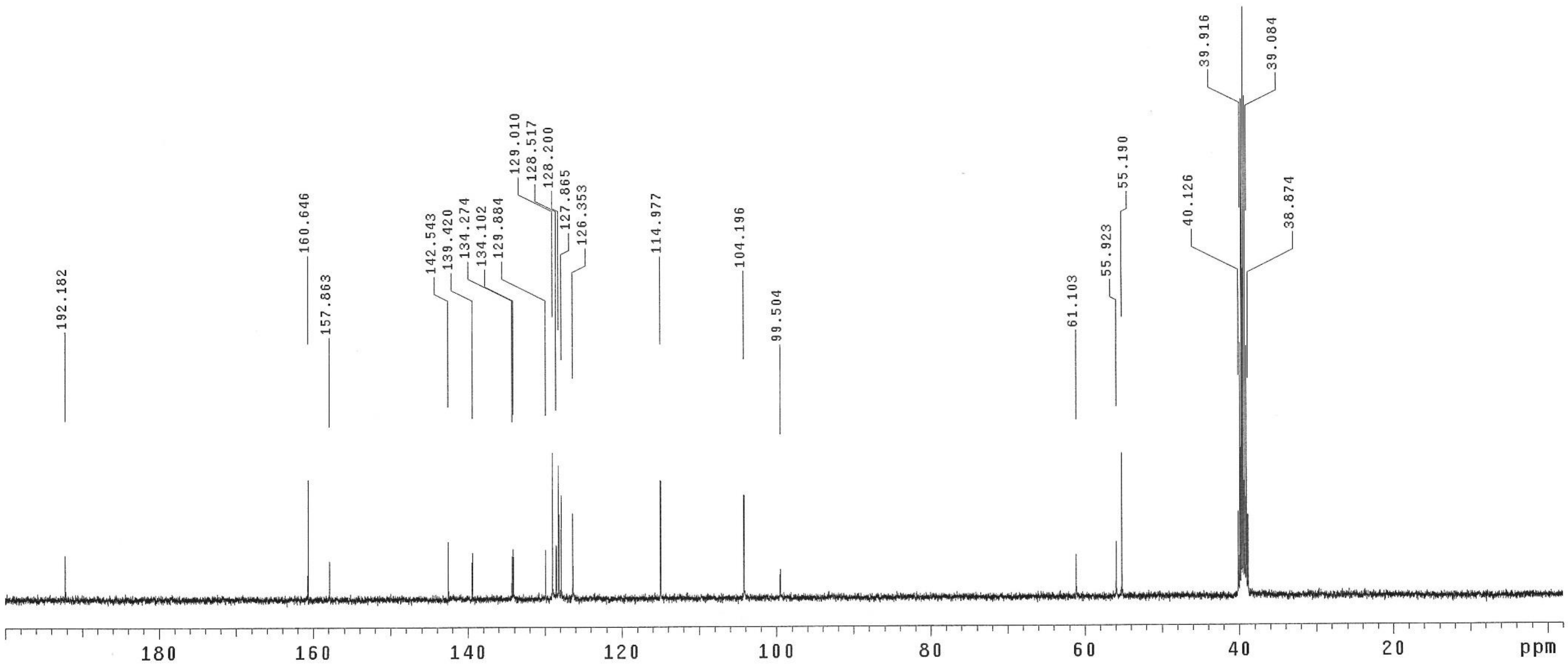

TKW-5930d

Pulse Sequence: s2pul
Mercury-400BB "MerPlus400"
Date: Feb 6 2018
Solvent: dmso
Ambient temperature
Total 32 repetitions

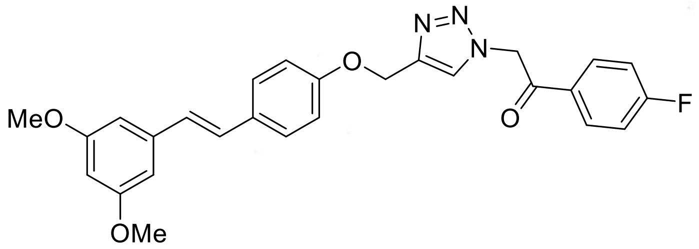

6b

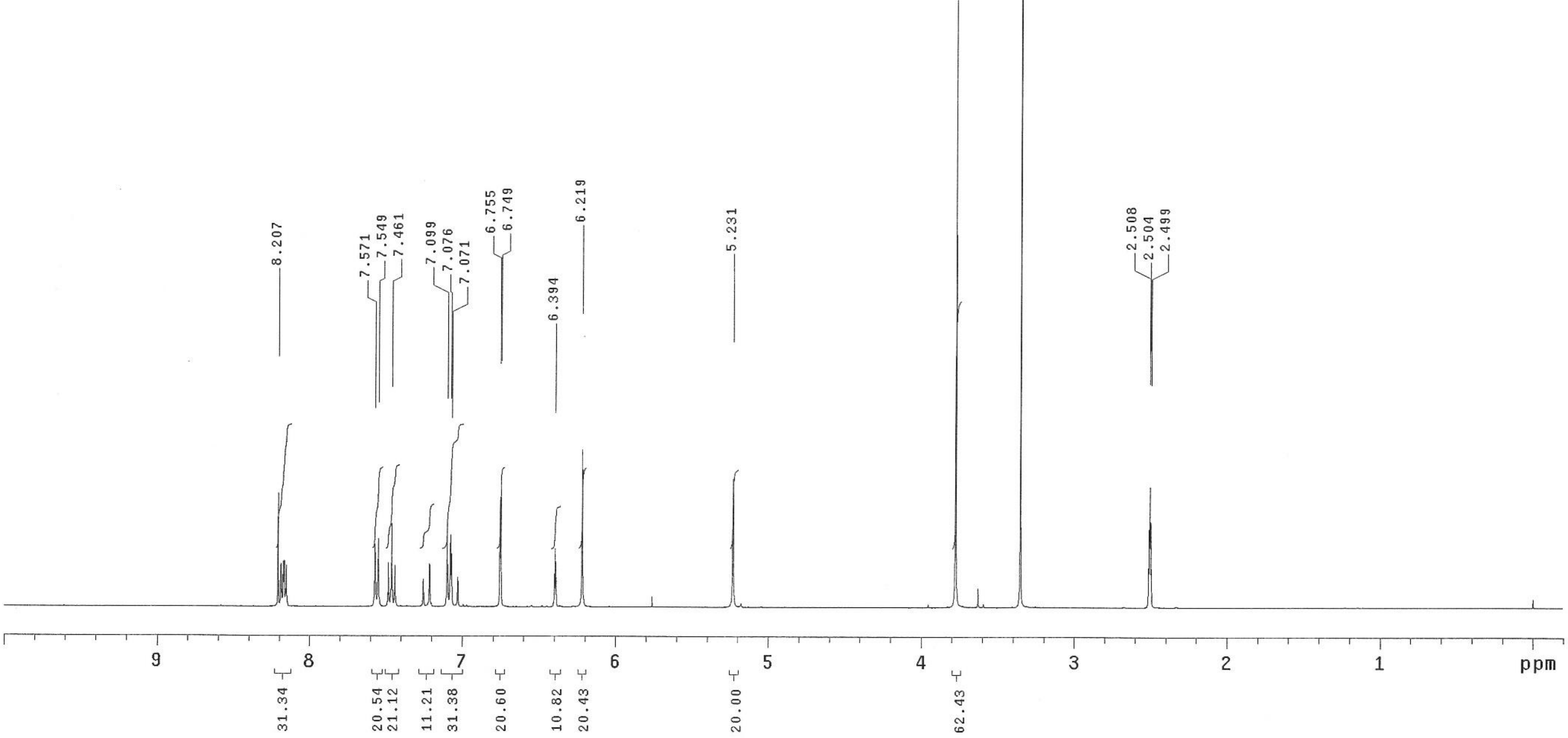

TKW-5930d

Pulse Sequence: s2pul
Mercury-400BB "MerPlus400"
Date: Feb 6 2018
Solvent: dmso
Ambient temperature
Total 4432 repetitions

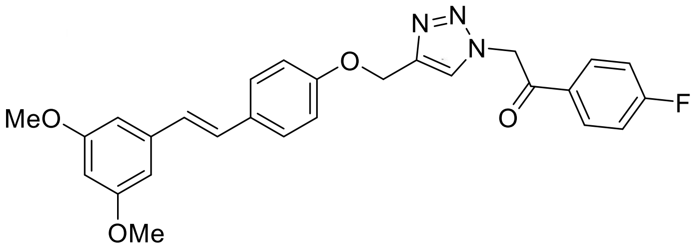

6b

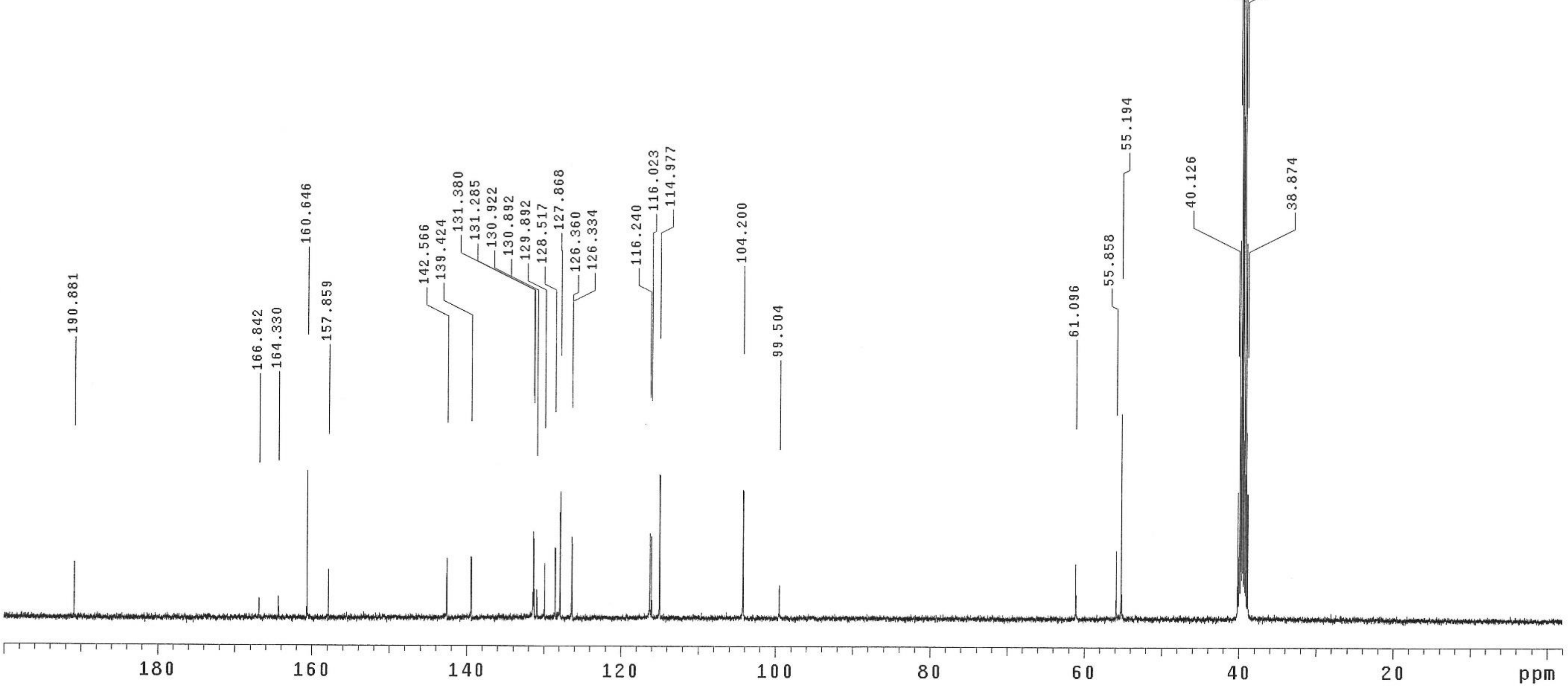

TKW-5931e

Pulse Sequence: s2pul
Mercury-400BB "MerPlus400"
Date: Feb 6 2018
Solvent: dmso
Ambient temperature
Total 32 repetitions

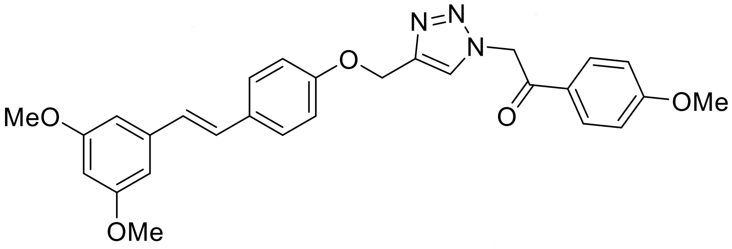

6c

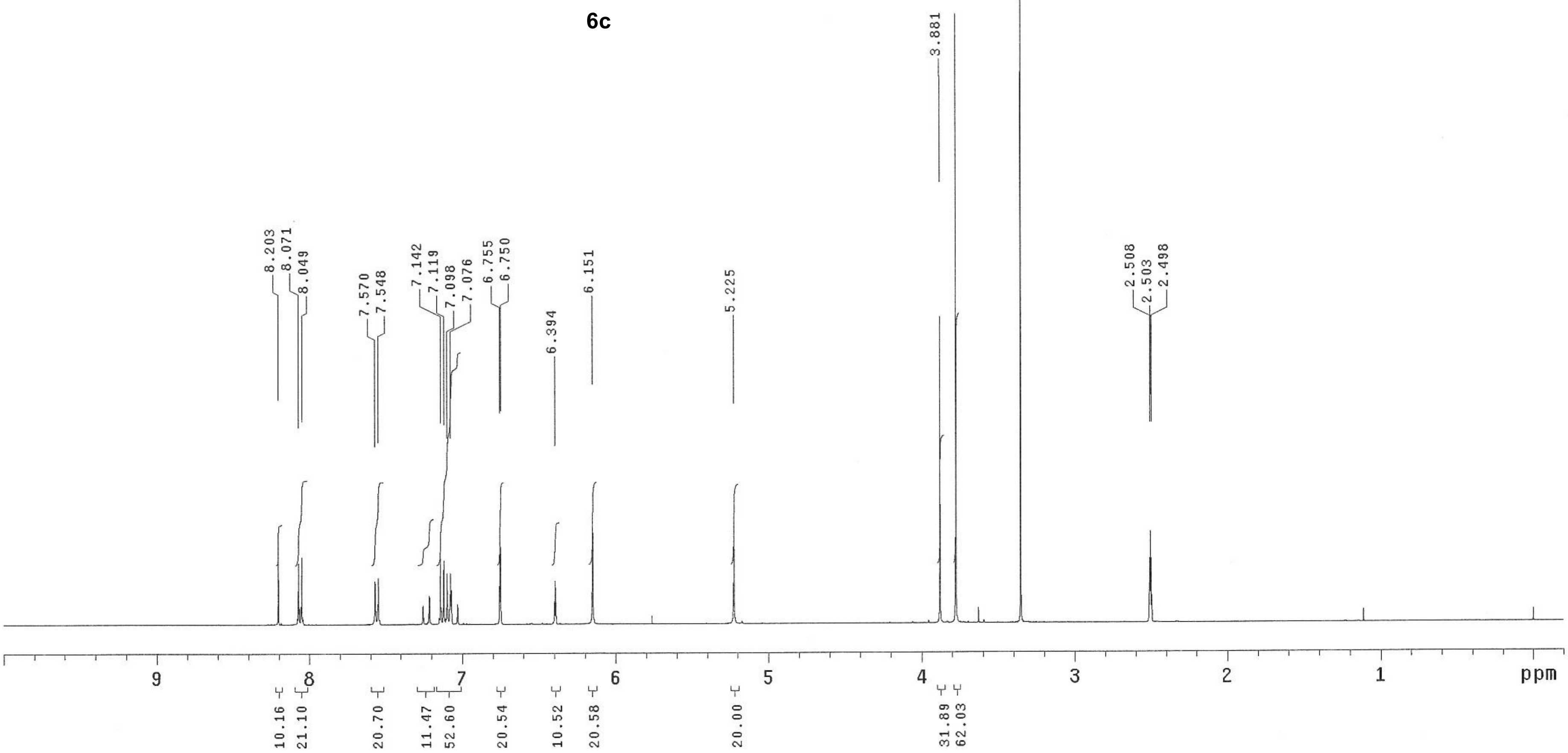

TKW-5931e

Pulse Sequence: s2pul
Mercury-400BB "MerPlus400"
Date: Feb 6 2018
Solvent: dmso
Ambient temperature
Total 2944 repetitions

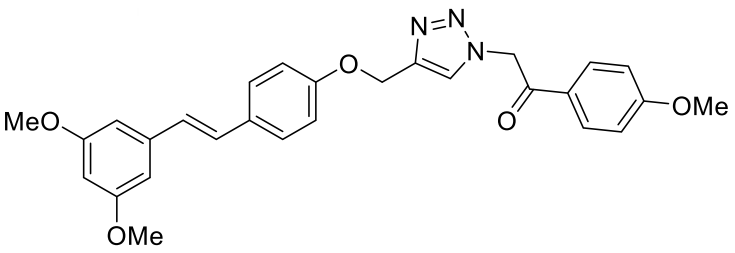

6c

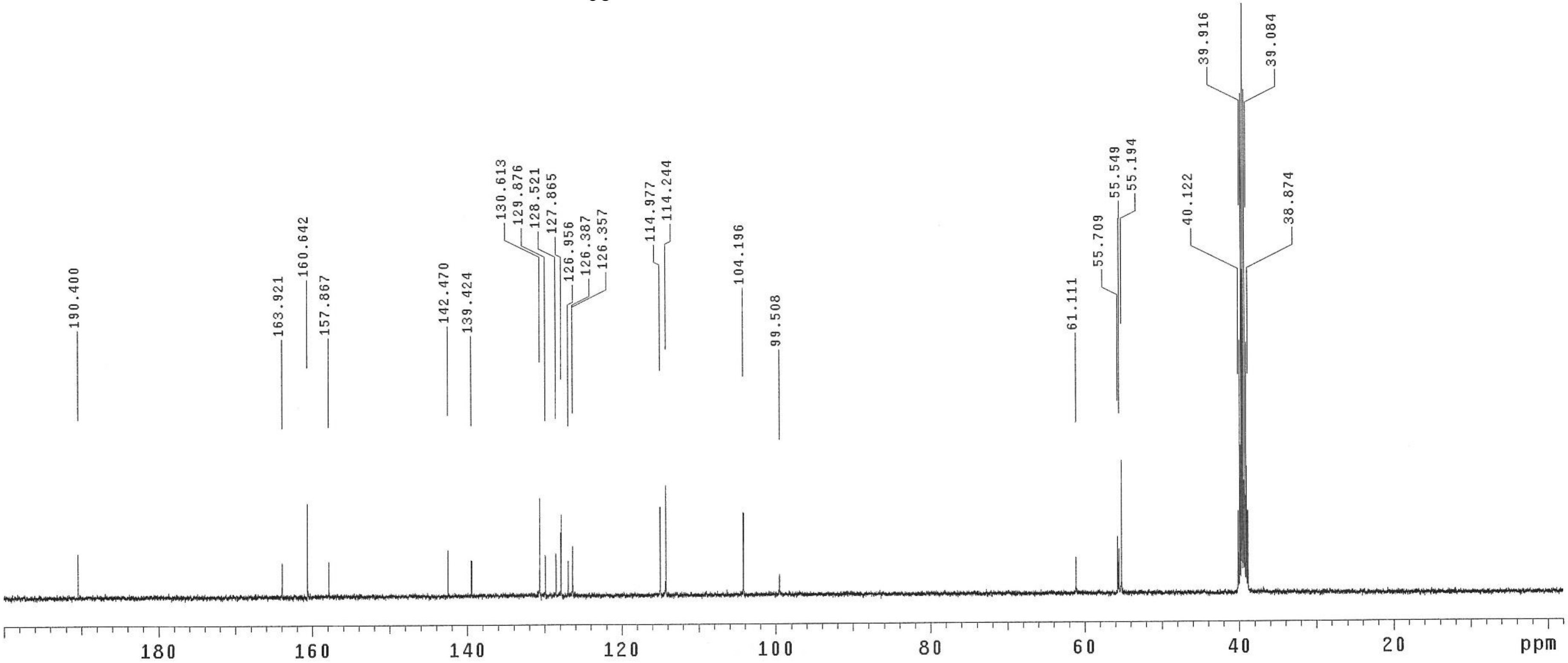

TKW-6108h

Pulse Sequence: s2pul
Mercury-400BB "MerPlus400"
Date: Jun 29 2018
Solvent: dmso
Ambient temperature
Total 64 repetitions

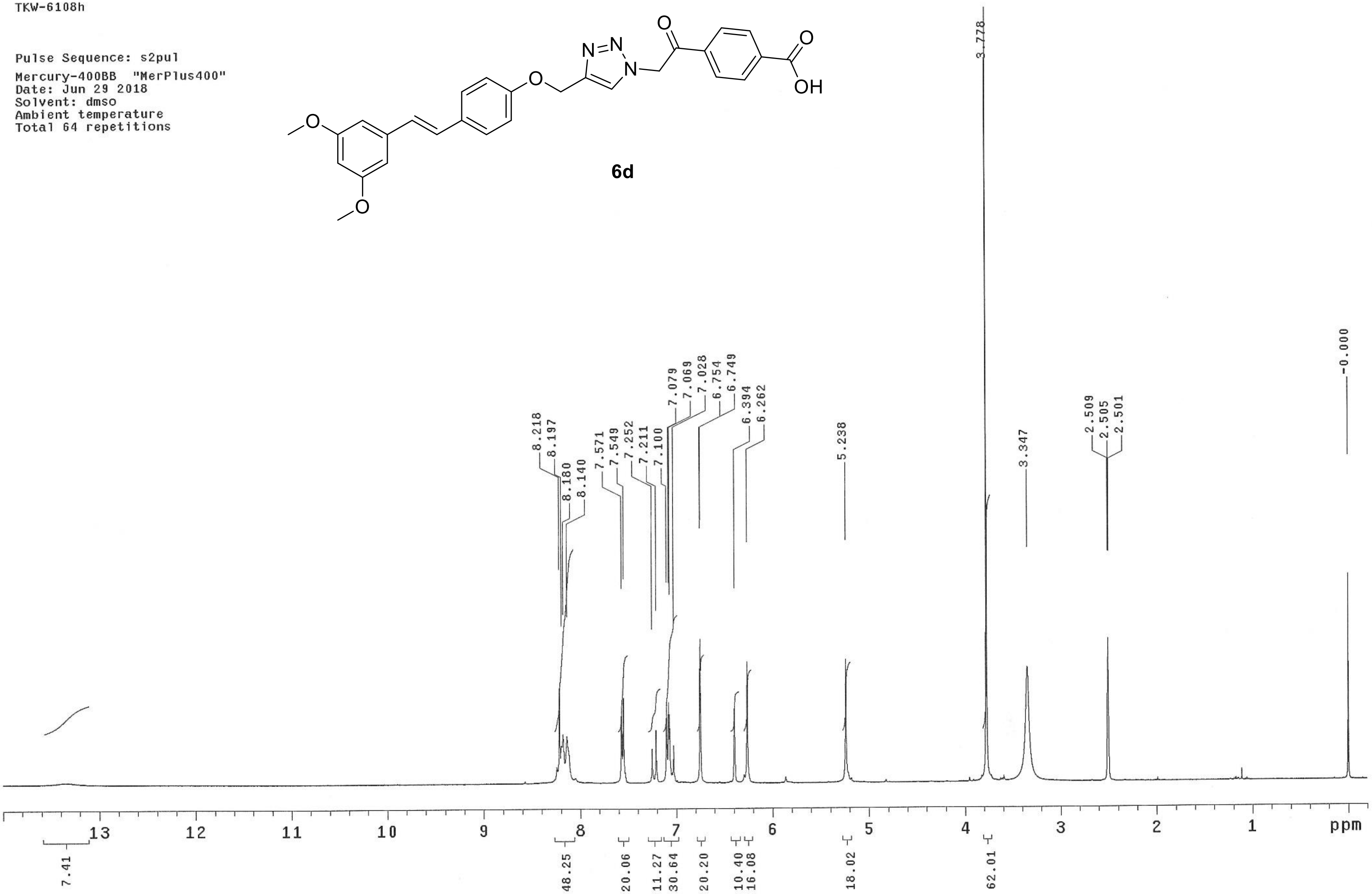

TKW-6108h

Pulse Sequence: s2pul
Mercury-400BB "MerPlus400"
Date: Jun 29 2018
Solvent: dmso
Ambient temperature
Total 8064 repetitions

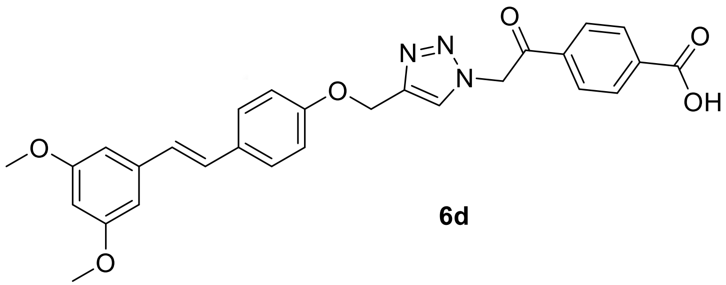

6d

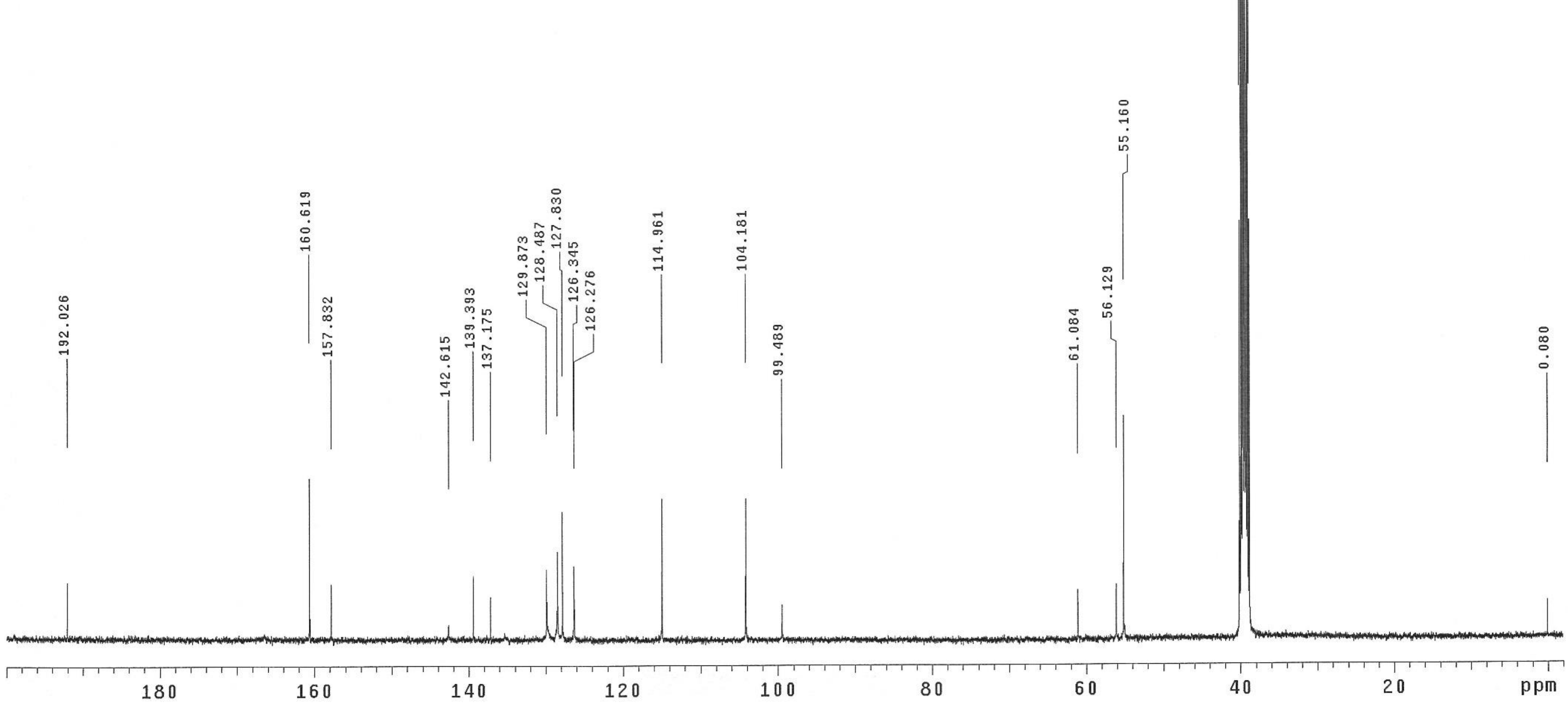

TKW-5932f

Pulse Sequence: s2pul
Mercury-400BB "MerPlus400"
Date: Mar 5 2018
Solvent: dmso
Ambient temperature
Total 32 repetitions

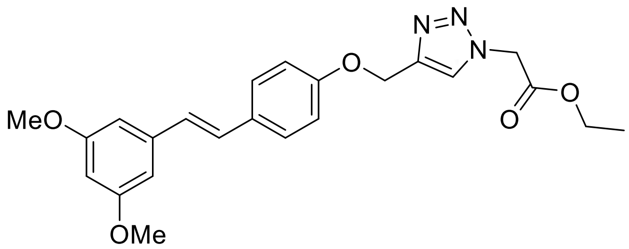

7a

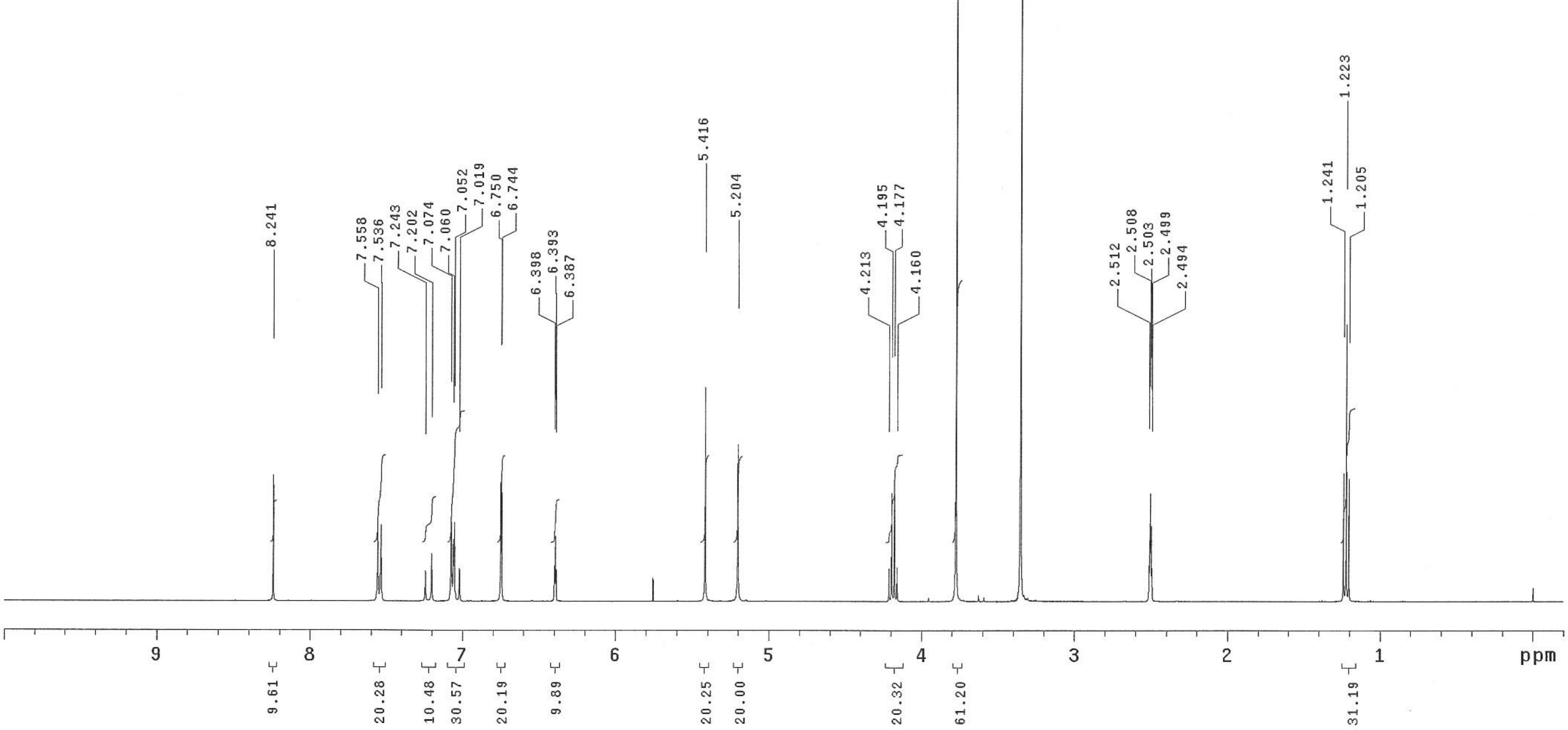

TKW-5932f

Pulse Sequence: s2pul
Mercury-400BB "MerPlus400"
Date: Mar 5 2018
Solvent: dmso
Ambient temperature
Total 64000 repetitions

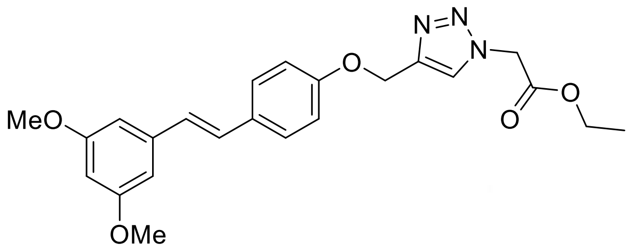

7a

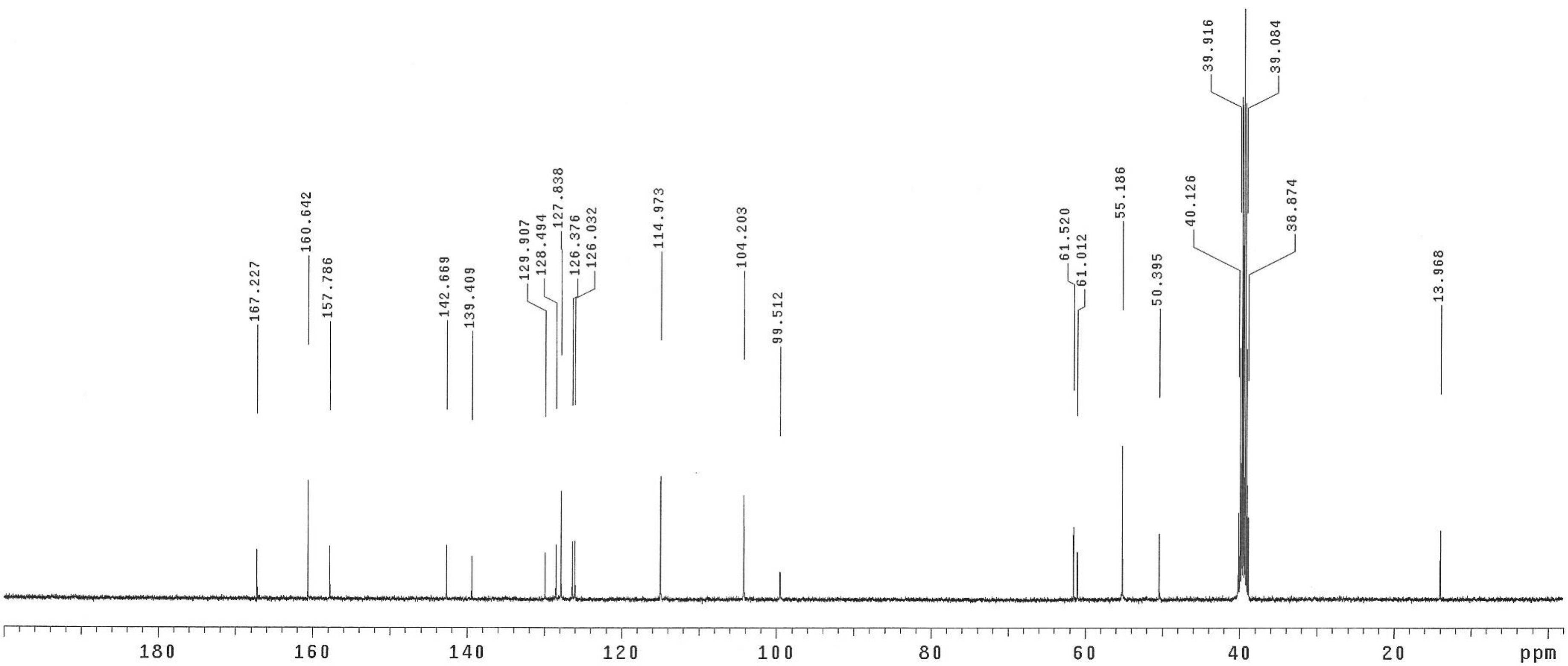

TKW-5933g

Pulse Sequence: s2pul
UNITYplus-400 "unity400"
Date: Mar 9 2018
Solvent: DMSO
Ambient temperature
Total 64 repetitions

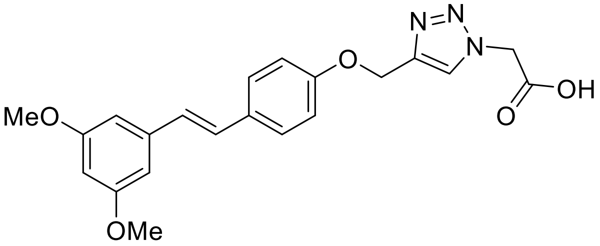

7b

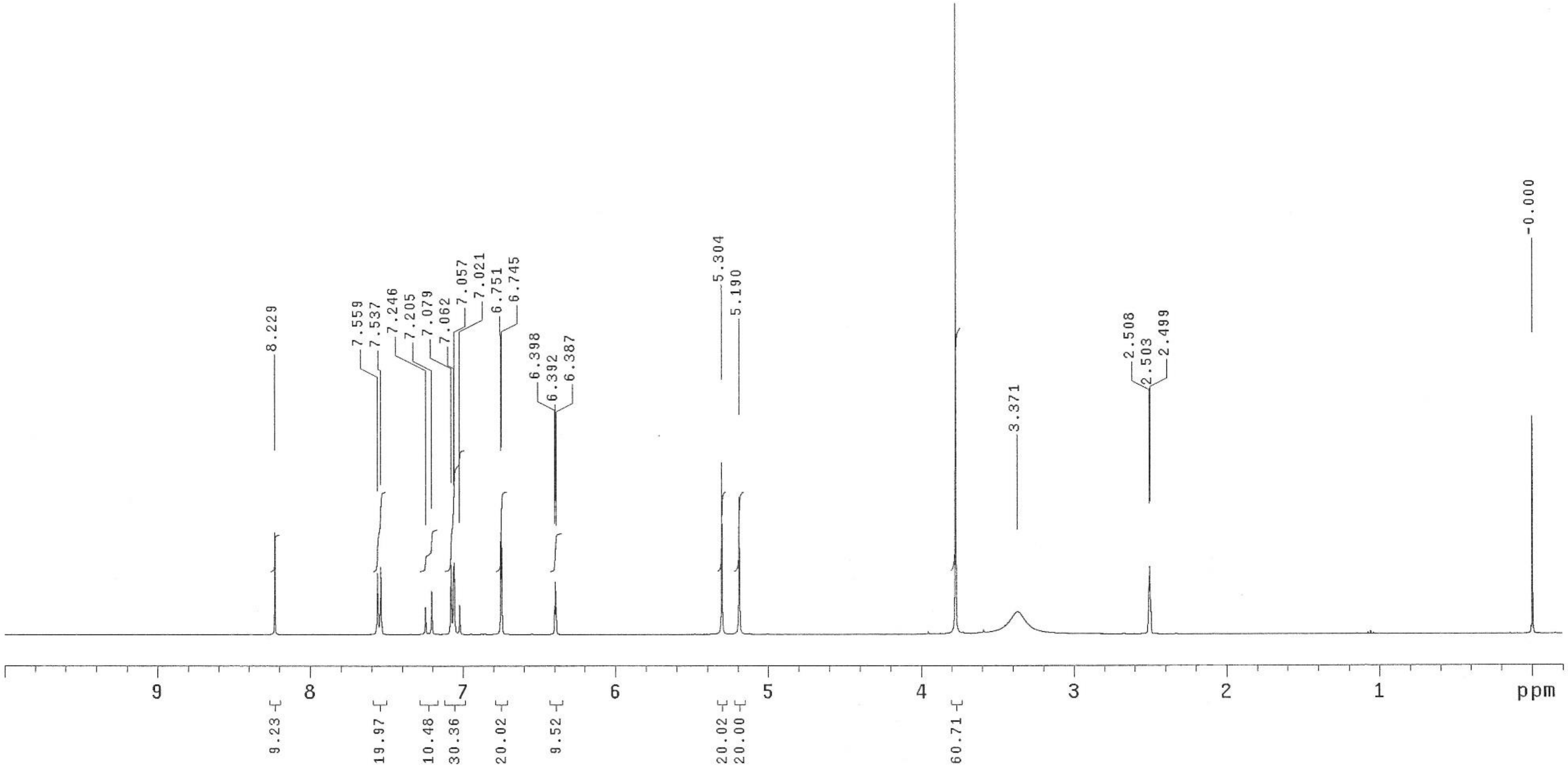

TKW-5933g

Pulse Sequence: s2pul

UNITYplus-400 "unity400"
Date: Mar 9 2018
Solvent: DMSO
Ambient temperature
Total 2160 repetitions

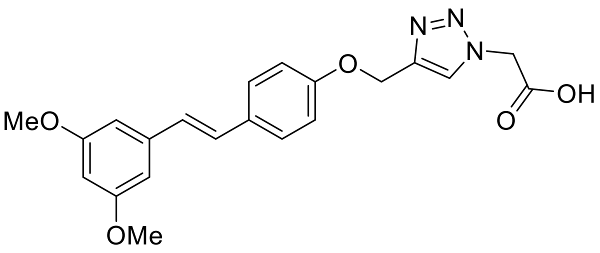

7b

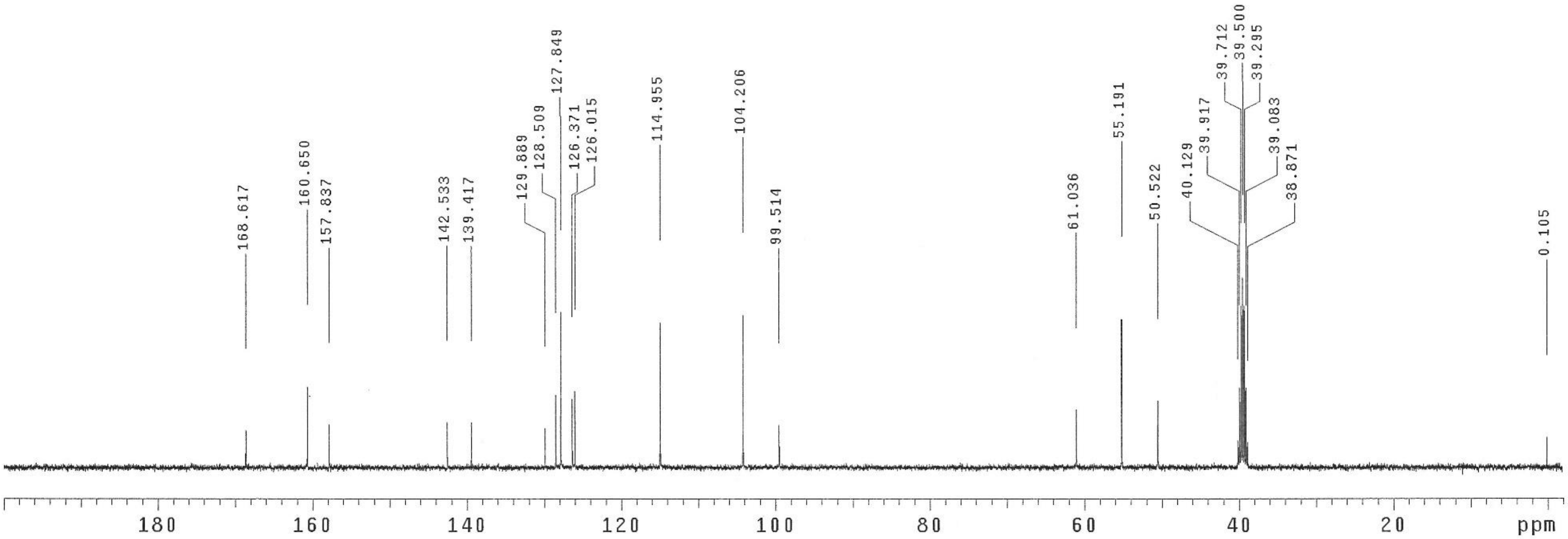

TKW-5934h

Pulse Sequence: s2pul
Mercury-400BB "MerPlus400"
Date: Mar 5 2018
Solvent: dmso
Ambient temperature
Total 32 repetitions

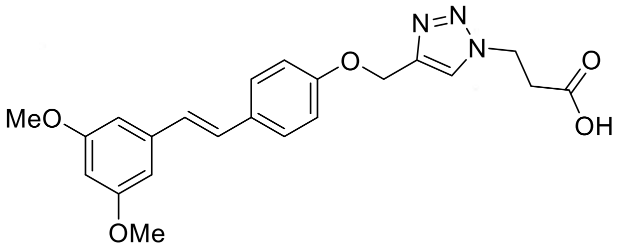

7c

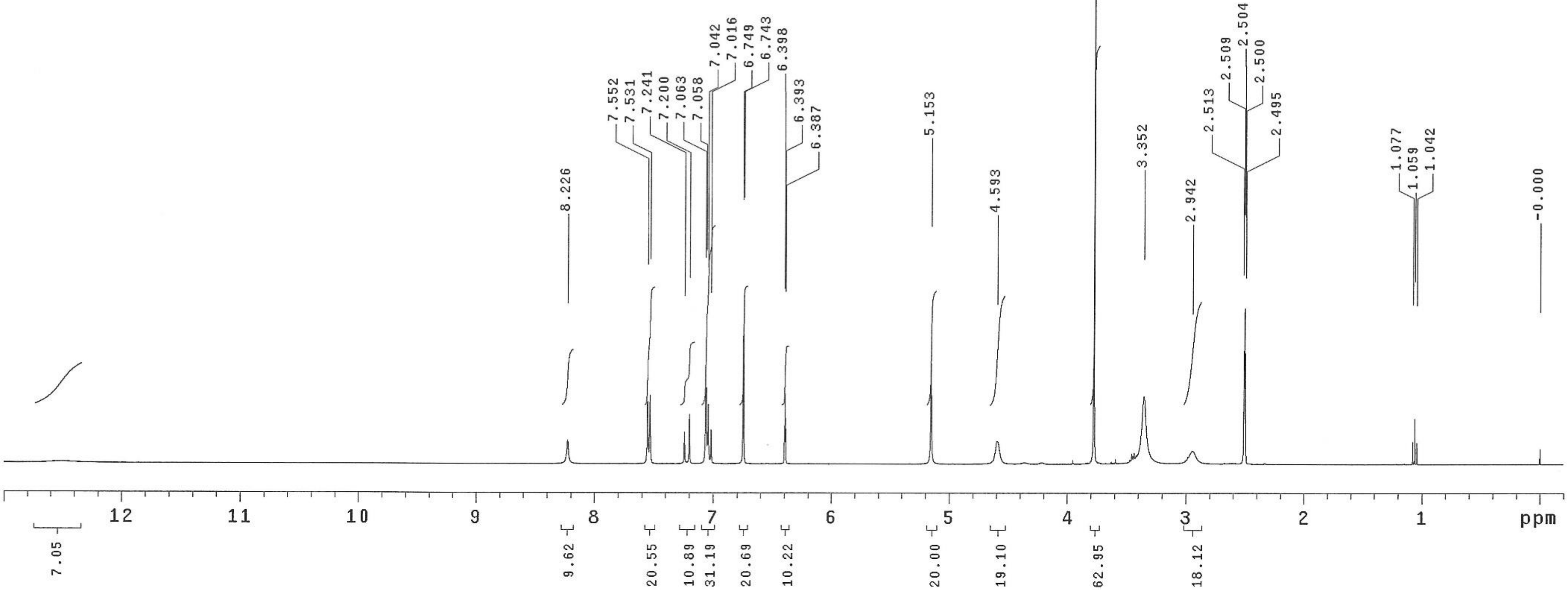

TKW-5934h

Pulse Sequence: s2pul
Mercury-400BB "MerPlus400"
Date: Mar 5 2018
Solvent: dmso
Ambient temperature
Total 4976 repetitions

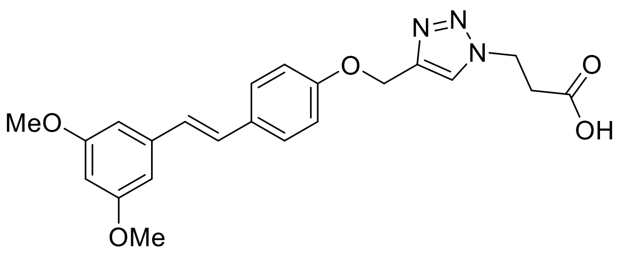

7c

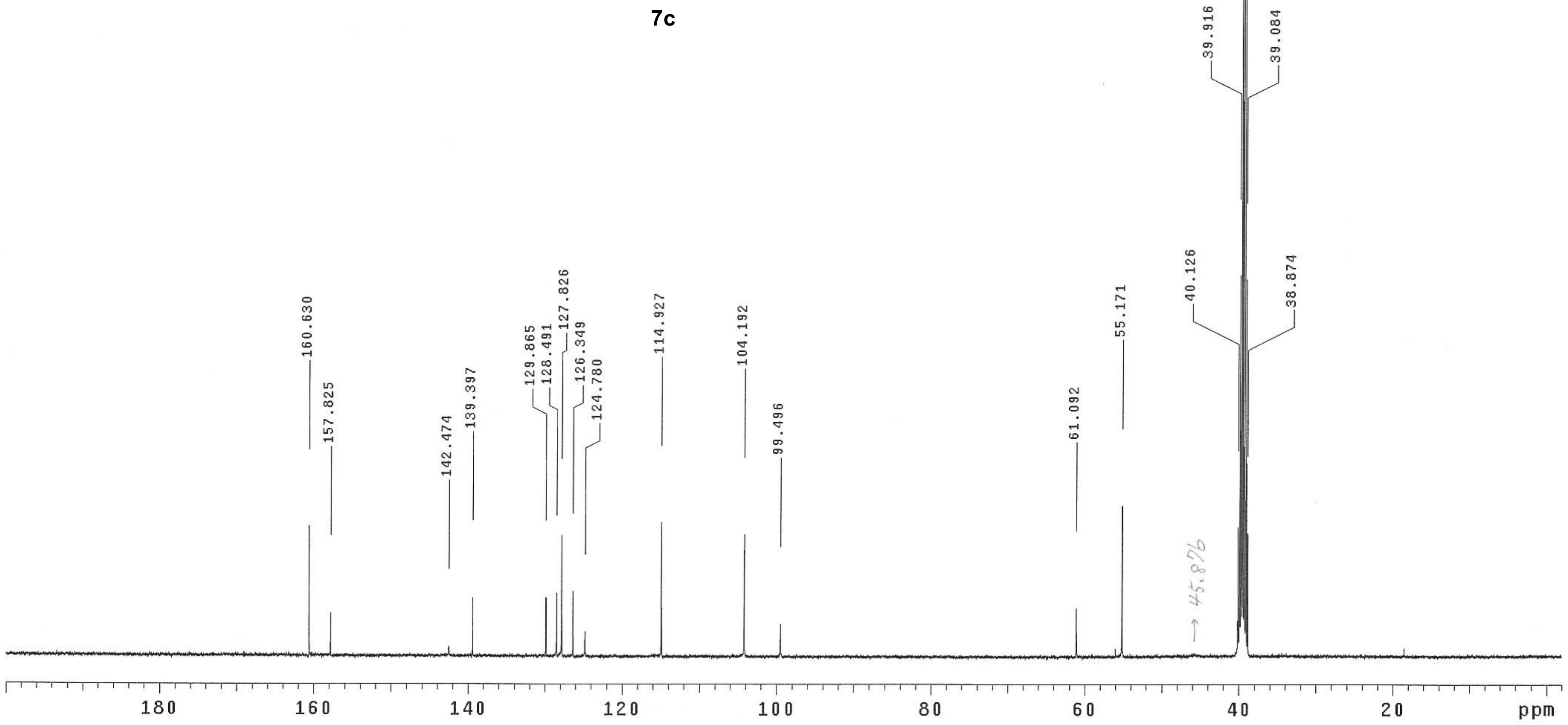

TKW-6109i

Pulse Sequence: s2pul
UNITYplus-400 "unity400"
Date: Jul 11 2018
Solvent: DMSO
Ambient temperature
Total 64 repetitions

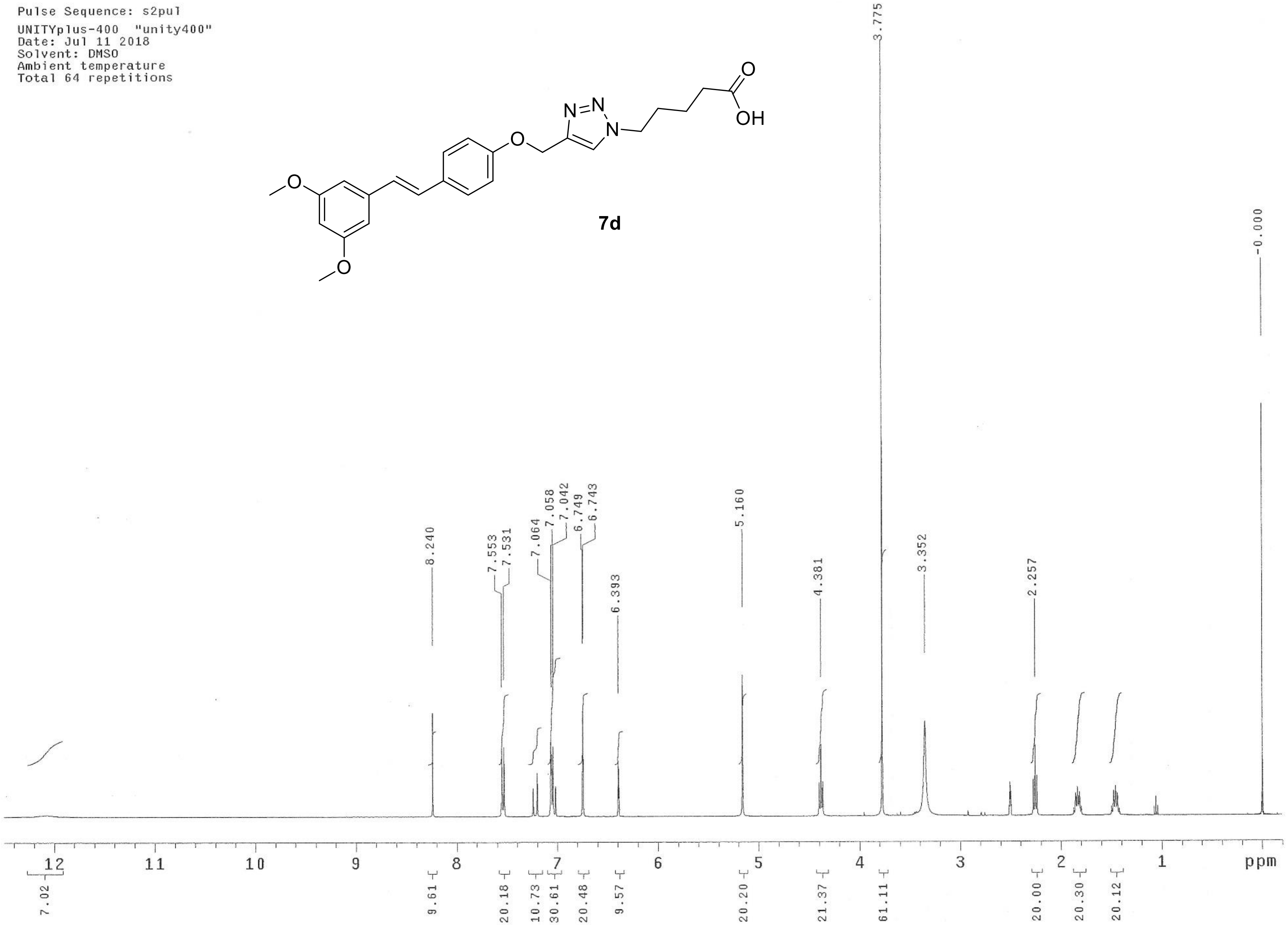

TKW-6109i

Pulse Sequence: s2pul

UNITYplus-400 "unity400"
Date: Jul 11 2018
Solvent: DMSO
Ambient temperature
Total 16000 repetitions

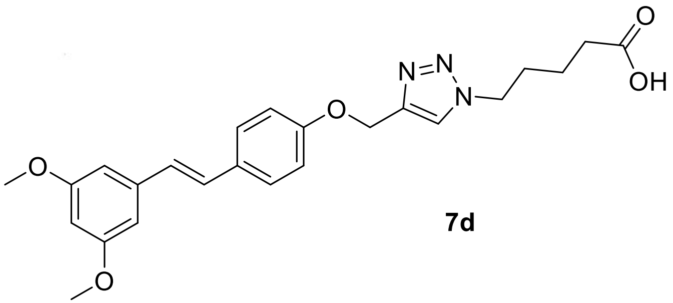

7d

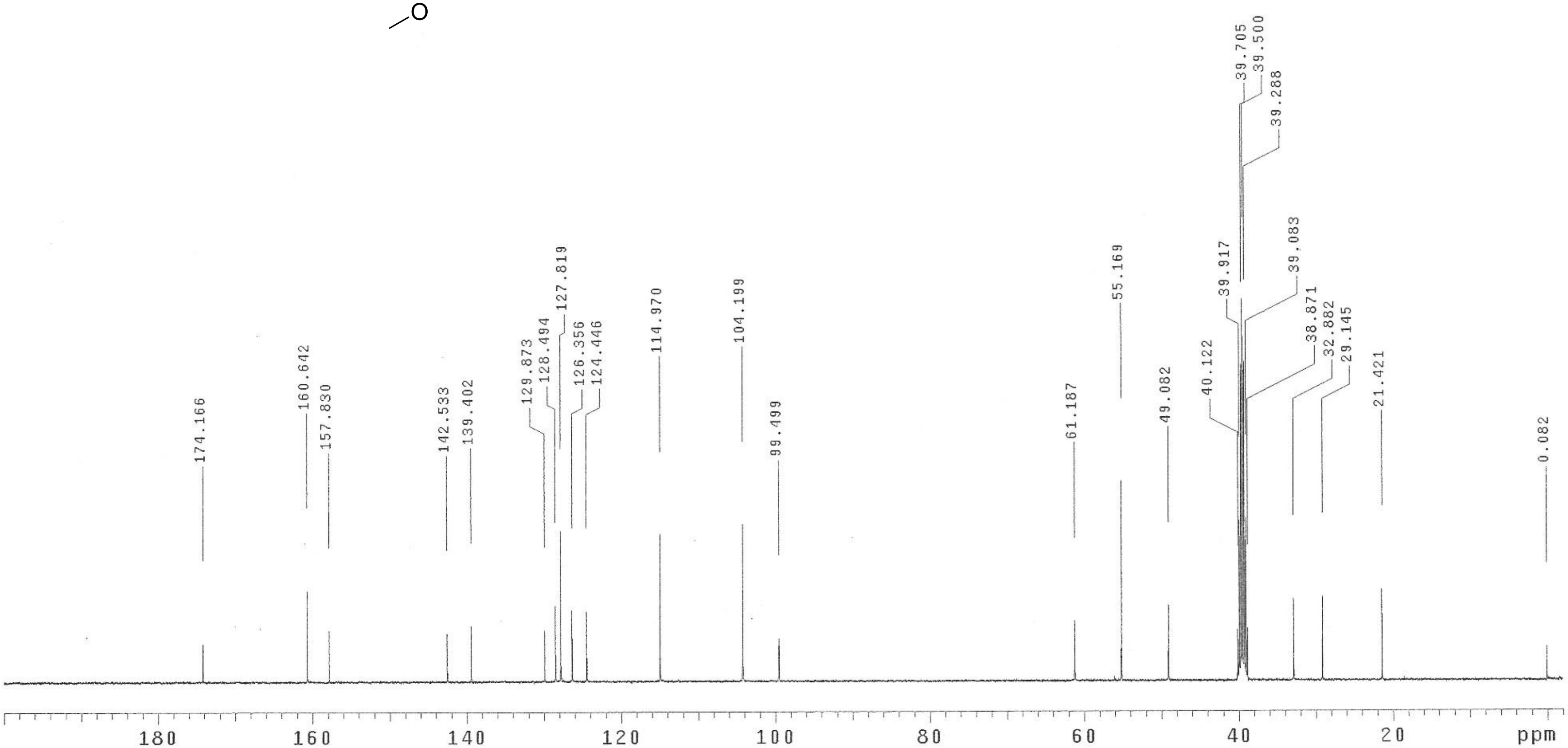

TKW-6110j

Pulse Sequence: s2pul
Mercury-400BB "MerPlus400"
Date: Jun 29 2018
Solvent: dmso
Ambient temperature
Total 32 repetitions

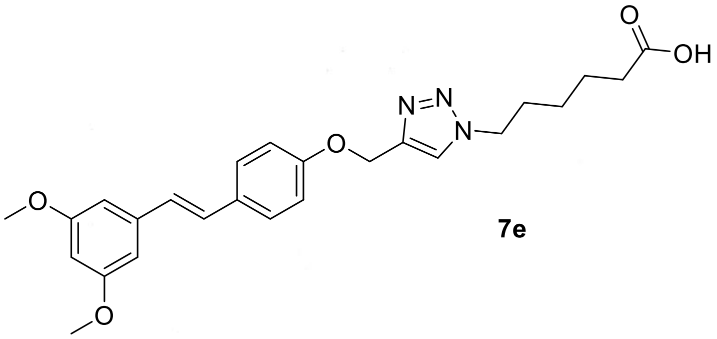

7e

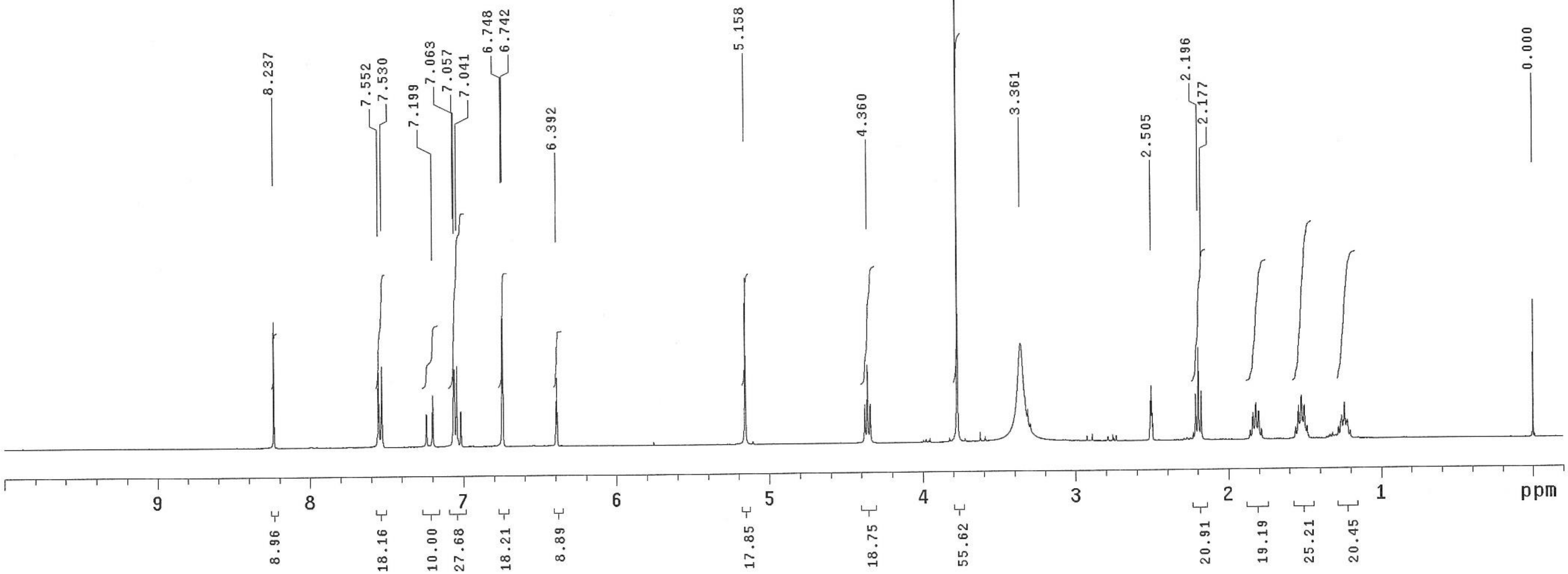

TKW-6110j

Pulse Sequence: s2pul

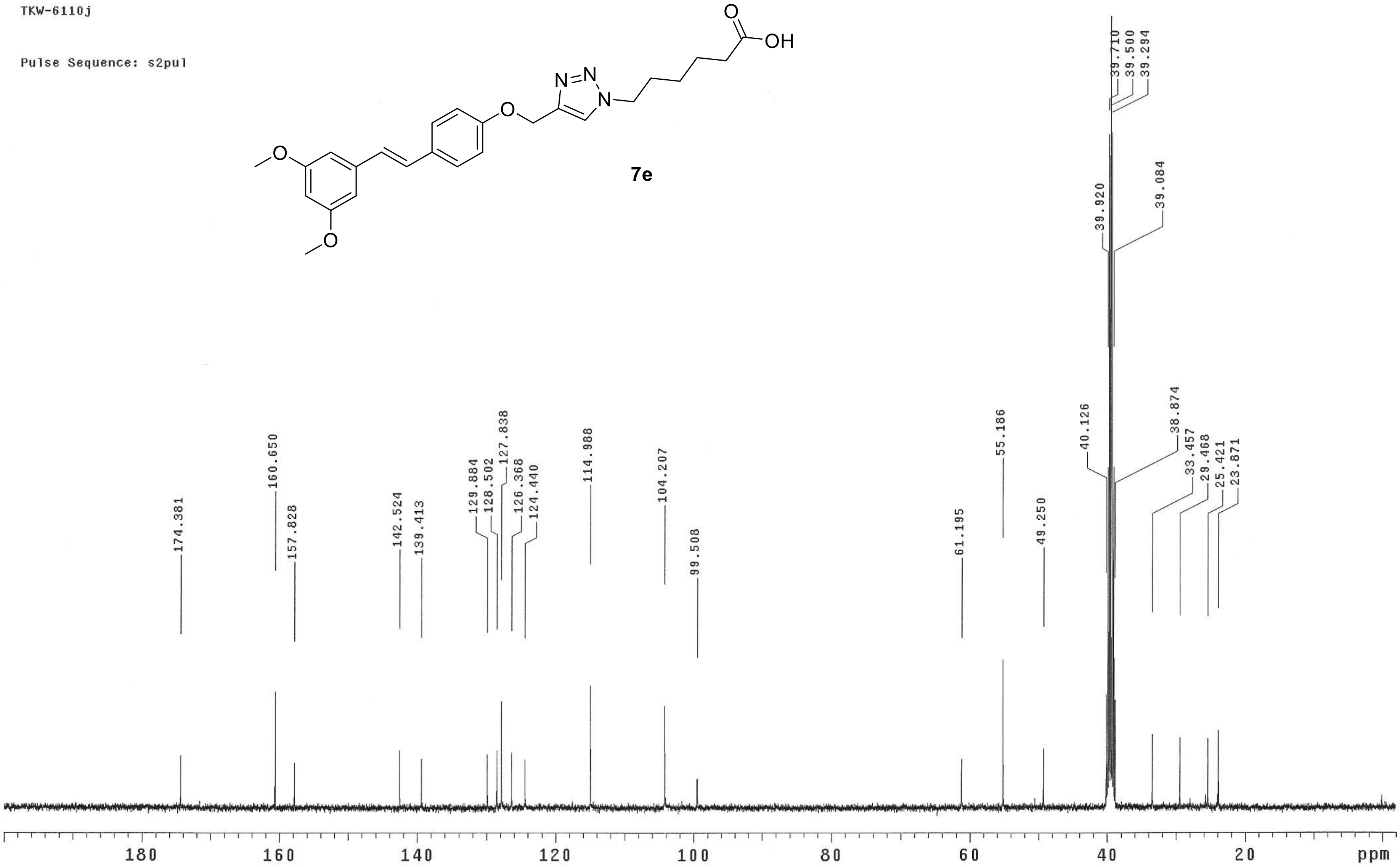

Supplement: Supplementary file 1 [file ijms-20-04564-s001.pdf]
